# The Plant Communities of the Class *Isoëto-Nanojuncetea* in Sardinia

**DOI:** 10.3390/plants14142187

**Published:** 2025-07-15

**Authors:** Salvatore Brullo, Gianluigi Bacchetta, Salvatore Cambria, Valeria Tomaselli, Gianpietro Giusso del Galdo, Pietro Minissale, Giovanni Rivieccio, Maria Carmela Caria, Simonetta Bagella

**Affiliations:** 1 Department of Biological, Geological and Environmental Sciences, University of Catania, Via A. Longo 19, 95125 Catania, Italy; cambria_salvatore@yahoo.it (S.C.); g.giusso@unict.it (G.G.d.G.); p.minissale@unict.it (P.M.); 2 Department of Life and Environmental Sciences, University of Cagliari, Viale Sant’Ignazio da Laconi 11/13, 09123 Cagliari, Italy; bacchet@unica.it; 3 Department of Biology, University of Bari “Aldo Moro”, Via Orabona 4, 70125 Bari, Italy; valeria.tomaselli@uniba.it; 4 Department of Chemistry, Physics, Mathematics and Natural Sciences, University of Sassari, Via Vienna 2, 07100 Sassari, Italy; grivieccio@uniss.it (G.R.); mccaria@uniss.it (M.C.C.); sbagella@uniss.it (S.B.)

**Keywords:** *Isoëto-Nanojuncetea* class, Sardinia, syntaxonomy, temporary ponds

## Abstract

A syntaxonomical revision of the plant communities of the *Isoëto-Nanojuncetea* class occurring in Sardinia is provided. Within this class, the ephemeral herbaceous hygrophilous associations linked to temporarily submerged surfaces occur, which are widespread in the European, Mediterranean, and Macaronesian countries. It groups plant communities floristically characterized by a rich set of annual hygrophytes or more rarely hemicryptophytes and geophytes, which are also physiognomically, ecologically, and structurally well differentiated. Within this class, two orders are recognized in Sardinia, such as *Isoëtetalia* and *Nanocyperetalia*, which are represented by several alliances. In particular, four alliances can be referred to as *Isoëtetalia* (*Isoëtion*, *Menthion cervinae*, *Cicendio-Solenopsion laurentiae,* and *Agrostion pourretii*), while a single alliance (*Verbenion supinae*) belonging to *Nanocyperetalia* has been identified. Within these alliances, several associations already described have been surveyed, while several other unpublished ones, are here proposed as new to science. Overall, 35 associations are recognized, 18 of which are described for the first time. Each higher-rank syntaxa and related associations are examined from a nomenclatural, floristic, ecological, and chorological point of view. In particular, the more significant phytosociological relevés regarding the examined associations were processed using cluster analysis, DCA ordination, optimclass diagram in order to highlight the correlations between them. As regards the floristic aspects, a checklist of the species occurring in the phytosociological relevés is provided.

## 1. Introduction

The ephemeral vegetation growing in the Euro-Mediterranean wetlands temporarily affected by more or less long periods of flooding by rainwater, is well differentiated from the floristic, ecological, and structural point of view compared to the other investigated plant communities. Previously, this vegetation was the object of several studies from which it can be deduced its complexity and diversification in relation to the edaphic, climatic, geographical, and environmental conditions [1,2,3,4,5,6,7,8,9,10,11,12,13,14].

These wet and very specialized habitats host a peculiar vegetation dominated mainly by therophytes, usually of small size, often growing together with geophytes and hemicryptophytes. The plant communities linked to these environments show a high floristic diversity for the occurrence of uncommon and ecologically very exigent hygrophilous species. On the whole, they can be considered as stenoecious species finding here their optimal growth conditions.

As concerns its distribution, this vegetation is widespread in the Euro-Mediterranean countries and Macaronesian area from sea level up to the high mountain belt, diversifying in relation to microclimatic and edaphic conditions. From the phytosociological viewpoint [5,7,9,10,12,14], the plant communities occurring in these temporary wetlands were referred to as the *Isoëto-Nanojuncetea* Br.-Bl. & R. Tx. ex Westhoff, Dijk & Passchier 1946, class well characterized in floristic, structural and ecological terms, although currently its syntaxonomic arrangement is still quite controversial, due to the various nomenclatural changes and updates made over time by several authors [15].

The remarkable variability of the floristic pool featuring the plant communities of this class must be sought in the substrate kind, bioclimatic conditions, elevation, period of submersion and extension of the surfaces relatively to the stands where they settle.

These wetlands, due to their geobotanical relevance and naturalistic value, are recognized by the Habitat Directive (43/92 EEC) as an environment of significant floristic and ecological importance, identifying them as a priority conservation habitat with the cod. 3170* (Mediterranean temporary ponds) [16]. Nevertheless, the habitat 3170* is a sub-type of another one, i.e., 3120-oligotrophic waters containing very few minerals, generally on sandy soils of the West Mediterranean, which is also referred to as the class *Isoëto-Nanojuncetea* [17,18,19].

Regarding the history of the investigations related to the syntaxa concerning the class *Isoëto-Nanojuncetea* it has been widely treated by Brullo et al. [15], a paper to which reference is made for further information.

In the frame of investigations on this class carried out in Italy, the results of a study concerning the plant communities currently occurring in Sardinia are presented here. They are based both on the literature data previously recorded from this island [20,21,22,23,24,25,26,27], but above all on unpublished phytosociological relevès carried out in the last decades by the authors.

As far as the rest of the Italian territory (Peninsula and Sicily), there is already a relevant literature which provides a fairly complete and extensive overview on this type of vegetation [28,29,30,31,32,33,34,35,36,37,38,39,40,41,42,43,44,45,46,47,48,49], to which two significant contributions regarding to Apulia [50] and Sicily [15] should be added.

Based on this data, the *Isoëto-Nanojuncetea* class is represented in Sardinia by two orders, such as *Isoëtalia* Br.-Bl. 1936 and *Nanocyperetalia* Klika 1935. The alliances belonging to the first order are *Isoëtion* Br.-Bl. 1936, *Menthion cervinae* Br.-Bl. ex Moor 1937 nom. mut., *Cicendio-Solenopsion laurentiae* Brullo & Minissale 1998, and *Agrostion pourretii* Rivas Goday 1958 nom. mut., while the second one is represented exclusively by *Verbenion supinae* Slavnić 1951.

In particular, all the syntaxa detected in this territory are examined under the floristic, ecological, physiognomic-structural, chorological, and nomenclatural viewpoints. Furthermore, in order to highlight the correlations between the plant communities identified, a statistical elaboration was carried out, which provided valid support for their syntaxonomic classification both at the level of association and of higher ranks.

## 2. Results and Discussion

### 2.1. Vegetation Analysis

**Figure 1:**
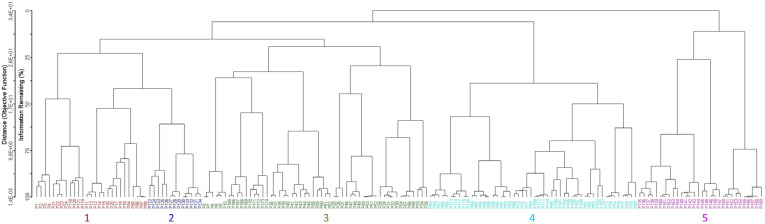
Dendrogram resulting from the cluster analysis of the data set; different colours correspond to different orders and alliances: A: *Isoëtetalia;* B: *Nanocyperetalia*; 1: *Isoëtion*; 2: *Agrostion pourretii*; 3: *Menthion cervinae*; 4: *Cicendio-Solenopsion laurentiae*; 5: *Verbenion supinae*.

Several clustering methods (e.g., flexible beta, Euclidean distance, Bray-Curtis, UPGMA) were used; according to Optimclass, the best partition is obtained through flexible beta Bray-Curtis [51]. The dendrogram was pruned at the level of 35 clusters of relevés. The groups thus identified correspond to the surveyed associations, wholly autonomous from a floristic and ecological point of view and well differentiated from each other. Overall, 35 associations, five alliances, and two orders were recognized, since arranged in distinct clusters. Indeed, as shown in Figure 1, two main clusters can be detected (A and B), which are separated into ecological groups.

**Figure 2:**
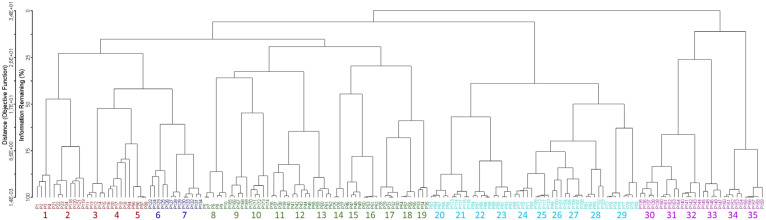
Dendrogram resulting from the cluster analysis of the data set, with the numbered list of associations examined A *Isoetetalia*; B *Nanocyperetalia*; (1) *Isoeto histricis-Montietum amporitanae*; (2) *Lythro hyssopifoliae-Silenetum laetae*; (3) *Buillardio vaillantii-Elatinetum campylospermae*; (4) *Lythro hyssopifoliae-Crassuletum vaillantii*; (5) *Romuleo requienii-Isoetetum histricis*; (6) *Anthoxantho aristati-Agrostietum pourretii*; (7) *Junco tingitani-Agrostietum pourretii*; (8) *Montio arvensis-Ranunculetum revelieri*; (9) *Callitriche stagnalis-Isoetetum longissimae*; (10) *Isoeto longissimae-Apietum crassipedis*; (11) *Middendorfio borysthenicae-Ranunculetum revelieri*; (12) *Isoeto tigulianae-Callitrichetum brutiae*; (13) *Loto conimbricensis-Ranunculetum revelieri*; (14) *Exaculo pusilli-Lythretum portulae*; (15) *Apio crassipedis-Isoetetum tigulianae*; (16) *Apio crassipedis-Elatinetum macropodae*; (17) *Ranunculo revelieri-Antinorietum insularis*; (18) *Apio crassipedis-Antinorietum insularis*; (19) *Isoeto tigulianae-Ranunculo lateriflori*; (20) *Junco capitati-Isoetetum histricis*; (21) *Solenopsio laurentiae-Lythretum tribracteati*; (22) *Archidio alternifolii-Isoetetum tigulianae*; (23) *Illecebro verticillati-Corrigioletum litoralis*; (24) *Solenopsio laurentiae-Isolepidetum cernuae*; (25) *Kickxio cirrhosae-Exaculetum pusilli*; (26) *Romuleo requieni-Bellidetum bellidioidis*; (27) *Romuleo requienii-Kickxietum cirrhosae*; (28) *Anagallido parviflorae-Molinerielletum minutae*; (29) *Cynosuro polybracteati-Antoxanthetum aristati*; (30) *Glino lotoides-Verbenetum supinae*; (31) *Sporobolo aculeati-Eryngietum pusilli*; (32) *Veronico beccabungae-Cyperetum fusci*; (33) *Pulicario vulgaris-Menthetum pulegium*; (34) *Sporobolo aculeatae-Pulicarietum siculae*; (35) *Cresso creticae-Sporoboletum aculeatae*.

The first to disjoin is cluster A, including the associations referable to *Isoëtetalia*, with winter-spring cycle, linked to periodically flooded surfaces drying up from late spring to early autumn. Within this order, the associations belonging to the alliances of *Isoëtion*, *Agrostion pourretii*, *Menthion cervinae,* and *Cicendio-Solenopsion laurentiae* are included (Figure 2). In particular, those belonging to *Isoëtion* are represented by plant communities subject to shorter periods of flooding, occurring on small surfaces, those referable to *Agrostion pourretii* occur on soils submerged for longer periods, often until late spring, and localized on wide surfaces, while the associations of the *Menthion cervinae* are linked to pools with deep waters persistent for a long time, finally, ones of *Cicendio-Solenopsion laurentiae* are localized on shallow sandy soils submerged in the winter-spring period. As regards cluster B, it is related to the plant communities of *Nanocyperetalia* with a summer-autumn cycle, with more or less nitrified soils, all belonging to *Verbenion supinae*.

### 2.2. Syntaxonomical Scheme of Isoëto-Nanojuncetea in Sardinia

Based on our investigations and the multivariate analysis of the phytosociological releves used for this study, and considering the updates regarding the arrangements of the *Isoëto-Nanojuncetea* class, the plant communities occurring in the Sardinian territory, are listed in the following syntaxonomical scheme:
 ISOËTO-NANOJUNCETEA Br.-Bl. & Tx. ex Westhoff, Dijk & Passchier 1946  ISOËTETALIA Br.-Bl. 1936 nom. conserv. propos.     *ISOËTION* Br.-Bl. 1936       (1) *Isoëto histricis-Montietum amporitanae* Biondi & Bagella 2005       (2) *Lythro hyssopifoliae-Silenetum laetae* Pisanu, Farris, Caria, Filigheddu, Urbani & Bagella 2014        (3) *Bulliardo vaillantii-Elatinetum campylospermae* Sciand., Miniss., Cambria, Ilardi & Giusso 2022       (4) *Lythro hyssopifoliae-Crassuletum vaillantii* Bagella, Caria, Farris & Filigheddu 2009       (5) *Romuleo requienii-Isoëtetum histricis* Bagella, Caria, Farris & Filigheddu 2009     *AGROSTION POURRETII* Rivas Goday 1958 nom. mut.       (6) *Antoxantho aristati-Agrostietum pourretii* Biondi & Bagella 2005 corr.        (7) *Junco tingitani Agrostietum pourretii* Brullo, Bacch., Giusso & Miniss. ass. nov.      *MENTHION CERVINAE* Br.-Bl. ex Moor 1937 nom. mut.     *APIENION CRASSIPEDIS* Bagella, Caria, Farris & Filigheddu 2009       (8) *Montio arvensis-Ranunculetum revelieri* Brullo, Bacch., Giusso & Miniss. ass. nov.        (9) *Callitricho stagnalis-Isoëtetum longissimae* Bagella, Rivieccio & Caria ass. nov.        (10) *Isoëto longissimae-Apietum crassipedis* Bagella, Rivieccio & Caria ass. nov.       (11) *Middendorfio borysthenicae-Ranunculetum revelieri* Barbero 1965, nom. mut. nov.       (12) *Isoëto tigulianae-Callitrichetum brutiae* Bagella, Caria, Farris & Filigheddu 2009       (13) *Loto conimbricensis-Ranunculetum revelieri* Brullo, Bacch., Giusso & Miniss. ass nov.       (14) *Exaculo pusilli-Lythretum portulae* Biondi & Bagella 2005       (15) *Apio crassipedis-Isoëtetum tigulianae* Biondi & Bagella 2005        (16) *Apio crassipedis-Elatinetum macropodae* Bagella, Caria, Farris & Filigheddu 2009       (17) *Ranunculo revelieri-Antinorietum insularis* Brullo, Bacch., Giusso & Miniss. ass. nov.        (18) *Apio crassipedis-Antinorietum insularis* Brullo, Bacch., Giusso & Miniss. ass.nov.        (19) *Isoëto tigulianae-Ranunculo lateriflori* Brullo, Bacch., Giusso & Miniss. ass.nov.      *CICENDIO-SOLENOPSION LAURENTIAE* Brullo & Minissale 1998       (20) *Junco capitati- Isoëtetum histricis* Br.-Bl. 1936        (21) *Solenopsio laurentiae-Lythretum tribracteati* Brullo, Bacch., Giusso & Miniss. ass. nov.        (22) *Archidio alternifolii-Isoëtetum tigulianae* Brullo, Bacch., Giusso & Miniss. ass. nov.        (23) *Illecebro verticillati-Corrigioletum litoralis* Brullo, Bacch., Giusso & Miniss. ass. nov.        (24) *Solenopsio laurentiae-Isolepidetum cernuae* Gehu, Kaabache & Gharzuoli 1994 *corr.*       (25) *Kickxio cirrhosae-Exaculetum pusilli* Brullo, Bacch., Giusso & Miniss. ass. nov.        (26) *Romuleo requieni-Bellidetum bellidioidis* Biondi & Bagella 2005        (27) *Romuleo requieni-Kickxietum cirrhosae* Brullo, Bacch., Giusso & Miniss. ass.nov.        (28) *Anagallido parviflorae-Molinerielletum minutae* Brullo, Scelsi, Siracusa & Tomaselli 1998        (29) *Cynosuro polybracteati-Antoxanthetum aristati* Brullo, Bacch., Giusso & Miniss. ass. nov.   NANOCYPERETALIA Klika 1935 nom. cons. propos.     *VERBENION SUPINAE* Slavnić 1951       (30) *Glino lotoidis-Verbenetum supinae* Rivas Goday 1964        (31) *Sporobolo aculeati-Eriyngietum pusilli* Brullo, Bacch., Giusso & Miniss. ass. nov.        (32) *Veronico beccabungae-Cyperetum fusci* Brullo, Bacch., Giusso & Miniss. ass. nov.        (33) *Pulicario vulgaris-Menthetum pulegium* Slavnić 1951        (34) *Sporobolo aculeati-Pulicarietum siculae* Brullo, Bacch., Giusso & Miniss. ass. nov.        (35) *Cresso creticae-Sporoboletum aculeati* Brullo, Bacch., Giusso & Miniss. ass. nov. 

For each of these syntaxa, the nomenclatural, floristic, ecological, and chorological characteristics are analyzed, as can be deduced from the literature data and unpublished field observations.

### 2.3. Description of the Vegetation

*ISOËTO-NANOJUNCETEA* Br.-Bl. & Tx. ex Westhoff, Dijk & Passchier 1946, Overz. Plantegem. Neder. 2.: 39.

Syn.: *Isoëto-Nanojuncetea* Br.-Bl. & Tx. 1943, Comm. S.I.G.M.A. 84: 7, nom. inval. (art. 2b, 8); *Isoëto-Nanojuncetea* Br.-Bl. & Tx. in Br.-Bl. et al., 1952, Group. Vég. Fr. Médit.: 80, nom. illeg. (art. 31); *Isoëtetea velatae* de Foucault 1988, Dissert. Bot. 121: 73; *Juncetea bufonii* de Foucault 1988, Dissert. Bot. 121: 78.

Lectotypus: *Isoëtetalia* Br.-Bl. 1936 nom. cons. propos.

Characteristic species: *Agathyron bufonium*, *A. hybridum*, *Damasonium bourgaei*, *Eryngium pusillum*, *Gaudinia fragilis*, *Juncinella capitata*, *Lythrum hyssopifolia*, *Mentha pulegium*, *Poa infirma*, *Polypogon subspathaceum*, *Pulicaria vulgaris*, *Ranunculus sardous*, *Verojuncus pygmaeus*.

Structure and ecology: It groups ephemeral amphibious vegetation colonizing the temporary wetlands with soils periodically flooded by oligotrophic, mesotrophic, eutrophic or, sometimes, brackish waters [12,52,53]. These plant communities belonging to this class are characterized by hygrophilous therophytes, sometimes mixed with small hemicryptophytes and geophytes. This vegetation is linked to very peculiar environments with the surfaces affected by temporary submersion, often alternating with marked aridity. This habitat, in the absence of environmental alterations, tends not to evolve towards more mature situations, and therefore the plant communities that characterize them can be considered as permaseries of vegetation [54].

Geographical distribution: The plant communities of this class are widespread in Europe and all Mediterranean territories, including the Macaronesian islands.

*ISOËTETALIA* Br.-Bl. 1936, Bull. Soc. Et. Sci. Nat. Nimes, 47: 142 nom. cons. propos. [55].

Syn.: *Isoëtetalia* Br.-Bl. 1931, Comm. S.I.G.M.A. 9: 38, nom. nud. (art. 2b); *Isoëtetalia velatae* de Foucault 1988, Dissert. Bot. 121: 73.

Type: *Isoëtion* Br.-Bl. 1936 conserved type proposed [55].

Characteristic species: *Archidium alternifolium*, *Briza minor*, *Bulliarda vaillantii*, *Centaurium maritimum*, *Elatine macropoda*, *Isoëtes longissima*, *Isolepis cernua*, *Lotus angustissimus*, *L. hispidus*, *L. parviflorus*, *Middendorfia borysthenica*, *Molineriella minuta*, *Myosotis sicula*, *Ranunculus muricatus*, *Romulea ramiflora*, *Trifolium micranthum*.

Structure and ecology: Pioneer ephemeral vegetation with thermophilous or subthermophilous requirements linked mainly to oligotrophic soils submerged up to springtime, sometimes flooded until early summer [12,52]. Usually, it is characterized by hygrophilous microphytes having an early spring blooming.

Geographical distribution: This order shows a Mediterranean and South Atlantic-European distribution.

*ISOËTION* Br.-Bl. 1936, Bull. Soc. Et. Sci. Nat. Nimes 47: 141.

Syn.: *Isoëtion* Br.-BI. 1931, Comm. S.I.G.M.A. 9: 38. nom. nud. (art. 2b); *Antinorio agrostideae-Isoëtion velatae* de Foucault 1988 Dissert. Bot. 121: 73, p.p.; *Ophioglosso lusitanici-Isoëtion histricis* de Foucault 1988, Dissert. Bot. 121: 74; *Elatino-Damasonion alismae* de FoucauIt 1988, Dissert. Bot. 121: 86, p.p.; *Crassulo-Lythrion borysthenici* de Foucault 1988, Dissert. Bot. 121: 90 p.p.;

Lectotypus: *Isoëtetum duriei* Br.-Bl. 1936.

Characteristic species: *Elatine campylosperma*, *Isoëtes durieui*, *I. histrix*, *Lotus conimbricensis*, *Ranunculus trilobus*.

Structure and ecology: Pioneer and fleeting vegetation localized mainly in small ponds with shallow waters, rich in quillworts and microphytes, showing an early spring blooming, linked to a warm Mediterranean climate. It colonizes small surfaces represented by rocky pools with very thin silty soils that dry up very early.

Geographical distribution: This alliance has a Mediterranean distribution.

#### 2.3.1. *Isoëto histricis-Montietum amporitanae* Biondi & Bagella 2005, Fitosociologia 42 (2) Suppl. 1: 17. (Table 1)

Holotypus: rel. 1, tab. 14 [21].

**Table 1:** *Isoëto histricis-Montietum amporitanae* Biondi & Bagella 2005.

Relevè Number	1	2	3	4	5	6
Altitude (m)	-	-	-	-	-	-
Surface (m^2^)	1	2	1	2	0.5	0.5
Coverage (%)	100	100	100	100	100	100
** Char. Association**						
*Montia hallii*	5	5	5	5	1	+
** Char. *Isoëtion* and *Isoëtetalia***						
*Isoetes histrix*	1	.	+	.	4	5
*Eudianthe laeta*	.	+	3	.	.	.
** Char. *Isoëto-Nanojuncetea***						
*Lythrum hyssopifolia*	.	.	.	1	.	.
*Verojuncus pygmaeus*	.	.	.	+	.	.
*Euphorbia falcata*	.	.	.	+	.	.
*Ranunculus ophioglossifolius*	.	.	1	.	.	.

**Localities and dates of relevés**: Rel. 1–6, La Maddalena Island, Biondi and Bagella (2005), Tab. 14.

Characteristic species: *Montia hallii* (*M. fontana* subsp. *amporitana*), *Isoëtes histrix*.

Structure and ecology: The association colonizes more or less flat surfaces on granitic substrate, covered by a thin layer of soil and flooded by stagnant or slightly flowing shallow waters, as in correspondence with small streams. In the stands with deeper waters, this vegetation is dominated by *Montia hallii*, a species usually linked to plant communities of the *Montio-Cardaminetea*, while in the less humid marginal places, the predominance of *Isoëtes histrix* is observed.

Geographical distribution: It was described from La Maddalena island in North-East Sardinia [21].

#### 2.3.2. *Lythro hyssopifoliae-Silenetum laetae* Pisanu, Farris, Caria, Filigheddu, Urbani & Bagella 2014, Plant Sociology 51 (1): 34 (Table 2)

Holotypus: rel. 16, tab. 3 [24].

**Table 2:** *Lythro hyssopifoliae-Silenetum laetae* Pisanu et al., 2014.

Relevè Number	1	2	3	4	5	6	7	8	9	10
Altitude (m)	-	-	-	-	-	-	-	-	-	-
Surface (m^2^)	1	0.5	0.5	2	30	3	5	15	8	5
Coverage (%)	90	100	100	100	100	90	90	90	100	100
** Char. Association**										
*Eudianthe laeta*	1	2	1	+	2	+	4	3	4	4
** Char.** ***Isoë*** ***tion* and ** ***Isoë*** ***to-Nanojuncetea***										
*Agathryon bufonium*	1	+	+	1	1	.	+	1	2	.
*Mentha pulegium*	+	+	+	1	+	1	.	.	+	.
*Lythrum hyssopifolia*	.	+	.	.	1	1	1	1	1	1
*Ranunculus sardous*	.	.	.	2	1	+	+	+	+	1
*Isolepis setacea*	4	4	3	3	.	1	1	.	.	.
*Polypogon subspathaceus*	.	.	.	.	+	+	+	1	.	.
*Gaudinia fragilis*	.	.	.	.	2	.	+	.	.	.
*Lotus conimbricensis*	.	.	.	.	.	.	+	+	.	.
*Verojuncus pygmaeus*	1	.	.	.	.	.	.	.	+	.
*Solenopsis laurentia* subsp. *laurentia*	.	.	.	.	.	1	.	+	.	.
** Other species**										
*Anagallis latifolia*	+	+	+	.	+	.	+	1	1	+
*Carex divisa*	+	.	.	.	1	+	.	1	.	1
*Anthemis arvensis*	+	+	+	1	.	.	.	.	.	.
*Lotus ornithopodioides*	.	1	2	+	.	.	.	.	.	+
*Hordeum marinum*	.	.	.	.	2	.	+	.	2	+
*Apium nodiflorum*	.	.	.	.	.	1	1	.	2	1
*Trifolium resupinatum*	.	.	.	.	+	.	.	+	+	+
*Agathryon subulatum*	.	.	.	.	1	.	2	.	1	.
*Bromus hordeaceus*	.	.	.	.	.	.	.	1	1	1
*Trigonella sicula*	.	.	.	.	.	.	+	+	1	.
*Rumex conglomeratus*	.	.	.	.	+	+	.	.	.	+
*Paspalum distichum*	.	+	+	.	.	.	.	2	.	.
*Baldellia ranunculoides*	.	.	+	r	.	.	.	.	.	.
*Ranunculus muricatus*	+	+	.	.	.	.	.	.	.	.
*Cynodon dactylon*	.	r	.	.	2	.	.	.	.	.
*Centaurium erythraea*	.	.	.	.	+	.	.	+	.	.
*Cyperus badius*	.	.	.	.	1	.	.	.	.	+
*Samolus valerandi*	.	.	.	.	+	3	.	.	.	.
*Lolium perenne*	.	.	.	.	1	.	.	.	.	.
*Plantago lanceolata*	.	.	.	+	.	.	.	.	.	.

**Localities and dates of relevés**: Rel. 1–10, Asinara Island, Pisanu et al. (2014), Tab. 3, rel. 8–17.

Characteristic species: *Eudianthe laeta*.

Structure and ecology: This association occurs in coastal stands, where it is localized, along the edges of small ponds and rivulets [24]. It shows an early spring phenology and is characterized by the occurrence of *Eudianthe laeta*, which usually grows together with other small hygrophytes, such as *Agathyron bufonium*, *Lotus coninbricensis*, *Lythrum hyssopifolia*, *Isolepis setacea*, *Mentha pulegium*, and *Ranunculus sardoum.* Moreover, the releves (rel. 8–11, tab. 3) referred by [24] to *Junco bufonii-Isolepidetum setacei* O. Bolòs & Masalles in O. Bolòs 1979, must be instead attributed to the association at issue, due to its floristic set and ecological requirements. The arrangement of these releves within the *Junco bufonii-Isolepidetum setacei* must be rejected, since this association represents a strictly autumnal vegetation with an entirely different floristic set [56].

Geographical distribution: Island of Asinara (NW Sardinia).

#### 2.3.3. *Bulliardo vaillantii-Elatinetum campylospermae* Brullo, Sciand., Miniss., Cambria, Ilardi & Giusso 2022, Plants 11, 1214: 19 (Table 3)

Holotypus: rel 14, tab. A7 [15].

**Table 3:** *Buillardio vaillantii-Elatinetum campylospermae* Brullo et al., 2022.

Relevè Number	1	2	3	4	5	6	7	8	9	10	11	12
Altitude (m)	580	580	580	580	580	580	580	580	580	580	10	10
Surface (m^2^)	1	1	1	1	1	2	2	1	1	2	2	1
Coverage (%)	90	100	60	60	50	90	70	70	90	80	80	90
** Char. Association**												
*Elatine campylosperma*	1	2	1	1	2	3	2	2	1	1	2	2
** Char.** ***Isoë*** ***tion* and ** ***Isoë*** ***tetalia***												
*Buillardia vaillantii*	3	2	2	3	2	2	3	3	4	3	4	3
*Damasonium bourgaei*	2	2	1	1	+	+	.	.	+	+	.	.
*Isolepis cernua*	2	1	.	1	1	+	.	+	+	.	.	.
*Pilularia minuta*	.	+	+	.	1	1	.	1	.	.	.	.
*Molineriella minuta*	2	3	2	2	1	2	2	2	2	3	.	.
*Callitriche brutia*	.	+	1	+	.	2	2	1	2	+	.	.
*Solenopsis laurentia* subsp. *laurentia*	.	+	.	.	+	+	+	.	1	.	.	.
** Char.** ***Isoë*** ***to-Nanojuncetea***												
*Agathryon bufonium*	1	1	.	+	1	1	.	+	1	+	1	2
*Poa infirma*	.	+	.	1	+	1	.	+	1	+	.	.
*Lythrum hyssopifolia*	2	1	1	2	+	1	1	2	2	2	.	.
*Polypogon subspathaceus*	2	2	1	+	1	1	+	.	+	+	.	.
*Mentha pulegium*	2	3	2	1	1	2	1	1	.	+	2	2
*Pulicaria vulgaris*	.	+	+	+	.	1	+	.	.	+	.	.
*Verojuncus tingitanus*	.	.	.	.	.	.	.	.	.	.	1	2
*Poa infirma*	.	.	.	.	.	.	.	.	.	.	+	.
** Other species**												
*Plantago coronopus*	1	2	2	2	+	1	2	1	2	1	+	+
*Spergularia rubra*	.	.	.	.	.	.	.	.	.	.	1	+
*Polygonum aviculare*	.	.	.	.	.	.	.	.	.	.	1	2

**Localities and dates of relevés**: Rel. 1–10, Giara di Gesturi, Pauli Caruso, 1 May 1995; Rel. 11–12, Asinara Island, 1 June 2002.

Characteristic species: *Elatine campylosperma*.

Structure and ecology: This association colonizes small pools, occurring in the basaltic or granitic outcrops localized along the edges of wide temporary ponds. These wetlands are flooded by rainwater during the autumn-winter period, often remaining submerged until late spring. In these stands, *Bulliarda vaillantii* is very frequent, growing usually together with *Damasonium bourgaei*, *Isolepis cernua*, *Pilularia minuta*, *Molineriella minuta*, *Agathryon bufonium*, *Callitriche brutia*, *Poa infirma*, *Lythrum hyssopifolia*, *Polypogon subspathaceus*, *Mentha pulegium*, etc. Moreover, it is significant that the occurrence of a very peculiar species of *Elatine*, which according to [57] must be referred to as *E. campylosperma*, a Mediterranean-Atlantic species with a very scattered distribution. Previously, the *Bulliardo vaillantii-Elatinetum campylospermae*, was described by [15] for some localities of western Sicily.

Geographical distribution: In Sardinia, this association was detected in some “paulis” of “Giara di Gesturi” in Central Sardinia and Asinara Island, where it is quite rare.

#### 2.3.4. *Lythro hyssopifoliae-Crassuletum vaillantii* Bagella, Caria, Farris & Filigheddu 2009, Fitosociologia 46 (1): 19 (Table 4)

Holotypus: rel. 6, tab. 10 [23].

**Table 4:** *Lythro hyssopifoliae-Crassuletum vaillantii* Bagella et al., 2009.

Relevè Number	1	2	3	4	5	6	7	8	9	10	11	12	13	14	15	16	17	18	19	20	21
Altitude (m)	120	120	120	10	10	1050	1050	1050	200	200	200	200	200	200	200	200	200	200	200	-	-
Surface (m^2^)	1	3	1	2	2	1	1	1	1	2	0.5	1	1	2	1	0.5	2	1	1	2	1
Coverage (%)	70	80	70	100	100	40	40	50	80	80	70	90	70	90	70	100	80	80	60	100	90
** Char. Association**																					
*Bulliarda vaillantii*	3	3	2	4	3	3	3	3	3	4	3	5	4	4	3	3	3	3	3	5	4
** Char.** ***Isoë*** ***tion* and ** ***Isoë*** ***tetalia***																					
*Lotus hispidus*	2	1	1	.	.	+	.	1	.	.	+	.	+	.	+	.	+	+	+	.	.
*Isoëtes histrix*	.	.	.	.	.	.	.	.	+	+	+	.	.	.	.	+	1	2	2	.	.
** Char.** ***Isoë*** ***to-Nanojuncetea***																					
*Lythrum hyssopifolia*	.	1	1	2	1	+	+	2	+	+	1	+	+	1	1	1	1	+	+	.	.
*Poa infirma*	.	.	.	.	.	2	+	1	+	.	+	+	+	1	1	1	1	1	+	.	.
*Agathryon hybridum*	.	.	.	.	.	.	.	.	1	+	+	+	+	1	1	+	1	2	1	.	.
*Agathryon bufonium*	+	1	1	.	.	2	2	1	.	.	.	.	.	.	.	.	.	.	.	.	.
*Mentha pulegium*	2	2	1	3	2	.	1	1	.	.	.	.	.	.	.	.	.	.	.	.	.
*Polypogon subspathaceus*	2	1	2	2	3	.	.	.	.	.	.	.	.	.	.	.	.	.	.	.	.
*Verojuncus pygmaeus*	.	.	.	.	.	.	.	1	.	.	+	.	.	.	+	.	.	.	.	+	+
*Middendorfia borysthenica*	3	4	3	.	.	.	.	.	.	.	.	.	.	.	.	.	.	.	.	.	.
*Eudianthe laeta*	.	.	.	.	.	.	.	.	+	+	.	.	.	.	.	.	+	.	.	.	.
*Juncinella capitata*	.	2	+	.	.	.			.	.	.	.	.	.	.	.	.	.	.	.	.
*Ranunculus lateriflorus*	.	.	.	.	.	.	+	+	.	.	.	.	.	.	.	.	.	.	.	.	.
*Centaurium maritimum*	.	.	.	+	+	.	.	.	.	.	.	.	.	.	.	.	.	.	.	.	.
*Solenopsis laurentia* subsp. *laurentia*	.	.	.	2	2	.	.	.	.	.	.	.	.	.	.	.	.	.	.	.	.
*Helosciadium crassipes*	.	.	.	.	.	.	.	.	1	.	.	.	.	.	.	.	.	.	.	.	.
*Isoëtes tiguliana*	.	.	.	.	.	.	.	.	.	.	.	.	+	.	.	.	.	.	.	.	.
** Other species**																					
*Plantago coronopus*	1	+	2	2	2	.	.	.	.	+	+	+	r	r	r	2	2	+	r	.	.
*Anthemis arvensis*	.	.	.	.	.	.	.	.	+	.	+	.	.	+	+	+	1	.	1	.	.
*Sedum caeruleum*	.	.	.	.	.	.	.	.	.	.	.	.	r	+	.	r	+	1	+	.	.
*Romulea columnae*	.	.	.	.	.	.	.	.	.	+	.	.	+	.	r	r	.	+	.	.	.
*Callitriche stagnalis*	.	.	.	.	.	.	.	.	.	2	.	+	.	+	+	.	.	.	.	.	.
*Bellis annua*	.	.	.	.	.	.	.	.	.	.	+	.	.	.	.	+	+	+	.	.	.
*Cynosurus polybracteatus*	.	.	.	.	.	+	.	1	.	.	.	.	.	.	.	.	.	.	.	.	.
*Plantago weldenii*	.	.	.	.	.	.	.	.	.	.	.	.	.	.	.	.	.	.	.	.	4
*Polygonum aviculare*	.	.	.	.	.	.	.	+	.	.	.	.	.	.	.	.	.	.	.	.	.
*Aira caryophyllea*	.	.	.	.	.	.	.	.	.	.	.	.	.	.	.	.	.	.	.	.	+

**Localities and dates of relevés**: Rel. 1–3, San Pietro Island, Contrada Paradiso, 12 May 1994; Rel. 4–5, San Pietro Island, La Punta, 12 May 1994: Rel. 6–8, Badde Salighes, Catena del Margine, 3 June 2002; Rel. 9–19, Olmedo (NW Sardinia), Bagella et al., (2009), tab. 10; Rel. 20–21, Asinara Island, Pisanu et al. (2014), tab. 3, rel 1–2.

Characteristic species: *Bulliarda vaillantii*, *Lythrum hyssopifolia*.

Structure and ecology: This association is localized mainly in wet pools occurring on rocky outcrops constituted by volcanic substrata or more rarely granites. These pools are periodically submerged by rainwater during the winter–spring season but dry up completely in the late spring. This ephemeral vegetation is dominated by *Bulliarda vaillantii*, growing usually together with other microphytes, such as *Lythrum hissopifolia*, *Agathyron bufonium*, *A. hybridum*, *Lotus hispidus*, *Isoëtes hystrix*, *Poa infirma*, etc. It shows some similarities with other plant communities of the *Isoëtion* physiognomically characterized by *Bulliarda vaillantii* described from some Mediterranean territories, as *Damasonio bourgaei-Crassuletum vaillantii* O. Bolòs & Llorens in O. Bolòs 1996, *Isoëto velatae-Crassuletum vaillantii* Poiron & Barbero 1965, *Myosuro-Bulliardetum vaillantii* Br-Bl. 1935, *Lythro thymifoliae-Crassuletum vaillantii* Rivas Goday ex Ruiz & A. Valdés 1987.

Geographical distribution: Based on literature data [23,24] and several unpublished relevés, the association was recorded for various localities of Sardinia, from sea level up to over 1000 m. It was previously also surveyed in Sicily by [15].

#### 2.3.5. *Romuleo requienii-Isoëtetum histricis* Bagella, Caria, Farris & Filigheddu 2009, Fitosociologia 46 (1): 16 (Table 5 and Table 6)

Holotypus: rel. 13, tab. 7 [23].

**Table 5:** *Romuleo requienii Isoëtetum histricis* Bagella et al., 2009.

Relevè Number	1	2	3	4	5	6	7	8	9	10	11	12	13	14	15	16	17	18	19	20	21	22	23	24	25	26	27	28	29
Altitude (dam)	58	58	58	58	58	58	20	20	20	20	20	20	20	20	20	20	20	20	20	20	20	20	20	20	20	20	20	20	20
Surface (m^2^)	2	3	3	2	3	2	1	1	1	2	1	4.5	1	1	1	2	2	2	14	20	27	12	20	18	8	34	9	1	2
Coverage (%)	80	70	80	80	80	80	70	70	80	100	90	90	80	100	100	95	100	90	90	90	90	90	90	85	90	90	100	70	100
** Char. Association**																													
*Isoëtes histrix*	3	2	3	3	3	3	1	1	1	4	4	3	3	4	4	2	3	3	4	2	3	4	3	4	4	3	4	4	4
*Romulea requienii*	1	1	1	.	1	+	r	.	.	+	+	1	1	1	1	2	2	1	1	1	1	1	1	1	1	1	2	1	1
** Char.** ***Isoë*** ***tion* and ** ***Isoë*** ***to-Nanojuncetea***																													
*Lythrum hyssopifolia*	+	2	1	1	2	1	1	+	1	+	+	+	1	.	.	.	r	r	+	+	+	+	+	1	+	+	1	1	+
*Lotus hispidus*	.	.	.	.	.	.	1	.	+	.	.	+	.	.	1	1	1	1	+	+	2	1	1	+	+	1	+	+	1
*Agathryon hybridum*	.	.	.	.	.	.	.	.	.	+	2	+	+	.	+	2	2	1	1	+	+	1	+	+	+	+	+	+	.
*Verojuncus pygmaeus*	.	.	.	.	.	.	.	.	.	.	.	+	.	.	.	.	.	+	2	1	2	1	1	1	1	+	+	.	r
*Poa infirma*	+	2	1	1	2	1	r	.	+	.	.	.	.	.	.	.	.	.	.	.	.	.	.	.	.	.	.	+	.
*Trifolium micranthum*	3	2	1	2	2	2	.	.	.	.	.	.	.	.	.	.	.	.	.	.	.	.	.	.	.	.	.	.	.
*Isolepis cernua*	.	.	.	.	.	.	.	.	.	.	.	2	.	.	.	.	.	.	1	2	2	+	.	.	.	+	.	.	.
*Polypogon subspathaceus*	1	.	2	2	2	2	.	.	.	.	.	.	.	.	.	.	.	.	.	.	.	.	.	.	.	.	.	.	.
*Myosotis sicula*	+	1	2	1	.	2	.	.	.	.	.	.	.	.	.	.	.	.	.	.	.	.	.	.	.	.	.	.	.
*Isoëtes tiguliana*	.	.	.	.	.	.	.	.	.	.	+	.	1	.	.	.	.	.	.	.	r	r	r	.	.	.	.	.	.
*Euphorbia falcata*	2	+	+		1	1	.	.	.	.	.	.	.	.	.	.	.	.	.	.	.	.	.	.	.	.	.	.	.
*Damasonium bourgaei*	.	+	+		2	2	.	.	.	.	.	.	.	.	.	.	.	.	.	.	.	.	.	.	.	.	.	.	.
*Hordeum geniculatum*	.	.	.	.	.	.	.	.	.	.	.	.	.	.	2	2	2	2	.	.	.	.	.	.	.	.	.	.	.
*Helosciadium crassipes*	.	.	.	.	.	.	.	.	.	.	.	r	.	.	.	.	.	.	r	r	.	.	.	.	.	.	.	.	r
*Agathryon bufonium*	.	.	.	.	.	.	3	3	3	.	.	.	.	.	.	.	.	.	.	.	.	.	.	.	.	.	.	.	.
*Ranunculus ophioglossifolius*	.	.	.	.	.	.	.	.	.	1	.	.	.	+	.	.	.	.	.	.	.	+	.	.	.	.	.	.	.
*Juncinella capitata*	.	.	.	.	.	.	.	1	2	.	.	.	.	.	.	.	.	.	.	.	.	.	.	.	.	.	.	.	.
*Buillardia vaillantii*	.	.	.	.	.	.	.	+	.	.	.	.	.	.	.	.	.	.	.	.	.	.	.	.	.	r	.	.	.
*Middendorfia borysthenica*	.	.	.	.	.	.	+	.	.	.	+	.	.	.	.	.	.	.	.	.	.	.	.	.	.	.	.	.	.
*Trifolium michelianum*	.	.	.	.	.	.	.	.	.	1	1	.	.	.	.	.	.	.	.	.	.	.	.	.	.	.	.	.	.
*Centaurium maritimum*	.	.	.	.	.	.	r	.	.	.	.	.	.	.	.	.	.	.	.	.	.	.	.	.	.	.	.	.	.
** Trasgr. *Cicendio-Solenopsion laurentiae***																													
*Cicendia filiformis*	.	.	.	.	.	.	+	.	.	.	.	.	.	.	+	+	+	.	r	.	+	.	.	+	r	+	+	.	1
*Ophioglossum lusitanicum*	.	.	.	.	.	.	.	.	.	.	.	+	.	.	+	+	+	.	.	.	.	.	.	+	+	1	+	.	r
*Solenopsis laurentia*	.	.	.	.	.	.	.	.	.	.	.	1	.	.	.	.	.	.	1	r	1	.	.	r	+	.	+	.	.
*Eudianthe laeta*	.	.	.	.	.	.	.	.	+	.	.	r	.	.	.	.	.	.	.	.	.	+	.	+	r	.	.	.	.
*Anagallis parviflora*	.	.	+	1	.	1	.	.	.	.	.	.	.	.	.	.	.	.	.	.	.	.	.	.	.	.	.	.	.
** Other species**																													
*Anagallis foemina*	.	.	.	.	.	.	+	r	.	+	.	+	.	.	.	.	.	.	+	+	+	1	+	1	+	+	1	.	+
*Bellis annua*	.	.	.	.	.	.	1	+	+	1	+	+	2	+	.	3	1	1	+	.	.	r	.	+	r	r	r	+	+
*Anthoxanthum aristatum*	.	.	.	.	.	.	.	.	+	.	.	1	.	.	2	2	.	.	+	+	+	+	r	+	+	+	+	.	1
*Euphorbia exigua*	.	.	.	.	.	.	.	.	.	+	.	.	.	.	1	1	1	1	.	1	.	r	1	+	+	+	+	.	+
*Romulea ligustica*	.	.	.	.	.	.	.	.	.	+	.	1	.	.	+	.	.	.	+	+	+	2	1	1	+	+	+	.	.
*Trifolium subterraneum*	.	.	.	.	.	.	+	+	r	.	.	+	.	.	.	.	.	.	+	+	r	+	+	+	.	.	r	.	+
*Anthemis arvensis*	.	.	.	.	.	.	.	2	1	.	+	.	.	.	1	1	1	2	.	.	r	r	.	.	.	.	.	1	+
*Plantago weldenii*	.	.	.	.	.	.	+	1	+	.	+	.	.	.	+	+	.	r	+	.	.	.	.	.	.	+	.	r	r
*Linum bienne*	.	.	.	.	.	.	.	.	.	.	.	1	.	.	.	.	.	.	2	1	1	+	1	1	1	1	1	.	.
*Logfia gallica*	.	.	.	.	.	.	.	.	.	.	.	.	.	.	.	.	.	.	1	r	+	+	+	+	+	r	+	.	.
*Carex flacca ssp. serrulata*	.	.	.	.	.	.	.	.	.	.	.	+	.	.	.	.	.	.	.	+	+	+	r	+	+	r	+	.	.
*Oenanthe lisae*	2	1	+	1	2	1	.	.	.	.	.	.	.	.	.	.	.	.	.	.	.	.	.	.	.	.	.	.	.
*Plantago coronopus*	2	2	2	2	1	2	.	.	.	.	.	.	.	.	.	.	.	.	.	.	.	.	.	.	.	.	.	.	.
*Trifolium angustifolium*	1	+	+	1		1	.	.	.	.	.	.	.	.	.	.	.	.	.	.	.	.	.	.	.	.	.	.	.
*Alopecurus bulbosus*	1	+	2			1	.	.	.	.	.	.	.	.	.	.	.	.	.	.	.	.	.	.	.	.	.	.	.
*Crepis bellidifolia*	1		+	+		1	.	.	.	.	.	.	.	.	.	.	.	.	.	.	.	.	.	.	.	.	.	.	.
*Cynosurus polybracteatus*	.	.	.	.	.	.	.	.	.	.	.	.	.	.	.	.	.	.	.	+	.	1	r	.	.	.	.	.	.
*Ranunculus paludosus*	.	.	.	.	.	.	.	.	.	.	.	+	.	.	.	.	.	.	.	+	r	.	.	r	.	.	.	.	.
*Lotus pedunculatus*	.	.	.	.	.	.	.	.	.	+	.	.	3	2	.	.	.	.	.	.	.	.	.	.	.	.	.	.	.
*Echium vulgare*	.	.	.	.	.	.	.	.	.	+	.	.	.	.	+	.	.	.	.	.	r	.	.	.	.	.	.	.	.
*Medicago minima*	.	.	.	.	.	.	r	+	r	.	.	.	.	.	.	.	.	.	.	.	.	.	.	.	.	.	.	.	.
*Romulea columnae*	.	.	.	.	.	.	.	.	.	1	1	.	.	.	.	r	.	.	.	.	.	.	.	.	.	.	.	.	.
*Asphodelus ramosus*	.	.	.	.	.	.	.	.	.	r	.	.	.	.	.	r	.	.	.	.	.	.	.	.	.	.	.	.	.

**Localities and dates of relevés**: Rel. 1–6, Giara di Gesturi, Pauli majore, 1 May 1995; Ril. 7–29, Olmedo (NW Sardinia), Bagella et al. (2009), Tab. 7.

**Table 6:** *Romuleo requienii Isoëtetum histricis* Bagella et al., 2009.

Relevè Number	30	31	32	33	34	35	36	37	38	39	40	41	42	43	44	45	46	47	48	49	50	51	52	53	54	55	56	57	58	59
Altitude (dam)	20	20	20	20	20	20	20	20	12	12	12	12	64	64	31	31	72	72	72	22	22	7	7	7	7	7	7	7	31	31
Surface (m^2^)	3	2	3	2	2	2	4	4	1	1	1	1	1	1	1	1	1	1	1	1	1	2	2	1	6	8	10	0.5	3	2
Coverage (%)	80	80	85	90	90	90	80	95	75	80	70	50	70	80	100	90	90	100	80	90	90	80	65	75	70	70	60	50	50	90
** Char. Association**																														
*Isoëtes histrix*	+	+	+	+	+	+	+	+	1	1	2	1	3	3	5	2	3	5	2	4	3	4	3	4	3	2	4	2	1	3
*Romulea requienii*	+	+	+	+	+	+	+	+	1	2	2	r	2	1	.	.	2	2	1	2	.	1	1	+	+	+	+	.	+	1
*Isoëtes gymnocarpa*	.	.	.	.	.	.	.	.	1	1	1	1	.	.	.	.	.	.	.	.	.	.	.	.	.	.	.	.	.	.
** Char.** ***Isoë*** ***tion*** **and** ***Isoë*** ***to-Nanojuncetea***																														
*Lythrum hyssopifolia*	+	2	2	1	+	+	1	1	.	+	.	+	1	1	.	.	2	.	.	1	+	1	1	+	1	+	1	2	+	+
*Lotus hispidus*	.	+	+	1	1	.	r	.	.	.	.	r	.	.	.	.	2	2	3	2	2	+	1	+	+	+	+	+	+	+
*Agathryon bufonium*	.	.	.	.	r	r	.	.	.	.	.	1	.	.	.	.	.	.	1	.	.	1	+	r	1	+	1	1	1	+
*Centaurium maritimum*	r	r	r	r	r	+	+	+	r	.	.	.	.	.	.	.	.	.	2	.	.	.	.	.	.	.	.	.	.	.
*Agrostis pourretii*	.	.	.	.	.	+	1	+	.	.	.	.	3	3	.	.	.	.	1	.	.	1	+	+	.	.	.	.	.	.
*Juncinella capitata*	.	r	1	+	+	r	+	+	.	.	.	.	.	.	.	.	.	.	.	.	2	.	.	.	.	.	.	.	.	r
*Verojuncus pygmaeus*	.	+	.	.	.	.	.	.	.	.	.	r	.	2	.	.	.	2	1	.	.	.	+	.	.	.	+	+	.	.
*Isolepis cernua*	+	.	+	.	r	+	.	.	.	.	.	r	.	.	.	.	.	.	.	.	.	.	.	.	.	.	.	.	.	.
*Ranunculus ophioglossifolius*	.	.	.	.	.	.	.	.	1	2	2	3	.	.	.	.	.	.	.	.	.	.	.	.	.	.	.	.	.	.
*Lotus conimbricensis*	.	.	.	.	.	.	.	.	.	1	.	.	.	.	.	1	.	.	.	.	.	.	.	.	.	.	.	.	.	.
*Romulea ramiflora*	.	.	.	.	.	.	.	.	.	.	r	+	.	.	.	.	.	.	.	.	.	.	.	.	.	.	.	.	.	.
*Lotus angustissimus*	.	.	.	.	.	.	.	.	.	.	.	.	.	.	2	1	.	.	.	.	.	.	.	.	.	.	.	.	.	.
*Poa infirma*	.	.	.	.	.	.	.	.	.	.	.	.	.	.	.	.	.	.	.	.	.	.	.	.	.	+	.	.	+	.
*Verojuncus tingitanus*	.	.	.	.	.	.	.	.	.	.	.	2	.	.	.	.	.	.	.	.	.	.	.	.	.	.	.	.	.	.
*Isoëtes tiguliana*	.	.	.	.	.	.	.	.	+	.	.	.	.	.	.	.	.	.	.	.	.	.	.	.	.	.	.	.	.	.
*Helosciadium crassipes*	.	.	.	.	.	.	.	.	.	.	.	.	.	.	.	.	.	.	.	.	+	.	.	.	.	.	.	.	.	.
*Mentha pulegium*	.	.	.	.	.	.	.	.	+	.	.	.	.	.	.	.	.	.	.	.	.	.	.	.	.	.	.	.	.	.
** Trasgr. *Cicendio-Solenopsion laurentiae***																														
*Ophioglossum lusitanicum*	r	.	+	.	.	+	+	+	.	.	.	.	+	.	1	1	.	.	.	.	1	1	+	1	+	1	+	.	r	r
*Cicendia filiformis*	2	1	2	2	2	1	1	1	.	.	.	.	+	2	.	.	.	.	2	.	1	.	.	r	.	.	.	+	.	r
*Solenopsis laurentia*	+	1	1	+	1	1	1	1				2	.	.	.	.	.	.	.	.	.	.	.	.	.	.	.	.	.	.
*Eudianthe laeta*	.	.	.	r	.	.	r	.	.	.	.	.	.	+	+	+	1	1	+	.	.	.	.	.	.	.	.	.	.	.
*Illecebrum verticillatum*	.	.	.	.	.	.	.	.	+	.	.	.	.	.	.	.	.	.	.	.	.	.	.	.	.	.	.	.	.	.
** Other species**																														
*Anagallis foemina*	+	+	+	r	+	+	+	+	1	1	2	r	1	1	2	1	1	2	1	2	3	1	+	+	.	.	.	.	.	.
*Bellis annua*	.	r	+	.	.	.	.	.	.	.	.	.	2	2	3	2	3	2	2	1	2	3	1	.	r	2	+	+	+	+
*Anthoxanthum aristatum*	+	1	1	.	+	+	+	1	.	.	.	.	2	2	3	3	2	3	2	2	3	+	.	+	.	+	+	+	.	+
*Linum bienne*	1	2	2	1	2	1	1	1	.	.	.	.	.	.	.	.	.	1	+	2	2	+	+	+	+	.	r	+	r	r
*Euphorbia exigua*	.	r	.	r	+	.	+	+	.	.	.	r	1	1	2	1	1	1	2	2	2	.	.	.	.	.	.	.	.	.
*Logfia gallica*	.	1	.	+	r	+	+	+	.	+	+	.	1	1	1	1	+	.	.	2	1	.	.	+	.	.	.	.	.	r
*Chamaemulum fuscatum*	.	.	.	.	.	.	.	.	1	1	.	.	3	+	.	.	1	1	.	.	.	2	+	.	+	3	2	+	2	.
*Cynosurus polybracteatus*	+	+	1	+	+	1	1	1	+	+	.	1	.	.	.	2	2	1	2	.	.	.	.	.	.	.	.	.	.	.
*Ranunculus paludosus*	r	.	.	.	r	r	r	r	.	.	.	.	.	.	2	1	1	1	+	1	1	.	.	.	.	.	.	.	.	.
*Trifolium subterraneum*	.	.	.	.	.	.	.	.	.	.	.	.	1	1	.	.	3	5	1	1	+	.	.	r	.	+	+	.	+	.
*Trachynia distachya*	1	1	1	2	1	2	1	1	.	.	.	.	.	.	.	.	.	.	1	.	.	.	.	.	.	.	.	.	.	.
*Bromus hordeaceus*	2	1	1	1	1	1	+	+	.	.	.	.	.	.	.	.	.	.	2	.	.	.	.	.	.	.	.	.	.	.
*Romulea ligustica*	.	.	.	.	.	.	.	.	2	2	2	.	.	.	3	1	1	+	.	3	2	.	.	.	.	.	.	.	.	.
*Polypogon maritimus*	+	2	1	1	2	+	+	1	.	.	.	.	.	.	.	.	.	.	.	.	.	.	.	.	.	.	.	.	.	.
*Vulpia bromoides*	+	2	1	1	1	+	2	1	.	.	.	.	.	.	.	.	.	.	.	.	.	.	.	.	.	.	.	.	.	.
*Macrobriza maxima*	.	.	.	+	+	r	+	+	.	.	.	+	.	.	.	.	.	.	.	.	1	.	.	.	.	.	.	.	.	.
*Cynodon dactylon*	.	.	.	.	.	.	.	.	.	.	+	+	2	3	.	1	2	.	.	.	.	.	.	.	.	.	.	.	.	.
*Hypochoeris radicata*	.	.	.	.	.	.	.	.	.	.	.	.	.	.	.	1	2	1	2	1	2	.	.	.	.	.	.	.	.	.
*Plantago weldenii*	.	.	.	.	.	.	.	.	.	.	.	.	.	+	.	+	2	2	3	.	+	.	.	.	.	.	.	.	.	.
*Carex flacca* subsp. *serrulata*	+	.	r	.	.	.	.	.	.	.	.	.	.	1	2	2	.	.	.	.	.	.	.	.	1	.	.	.	.	.
*Tuberaria guttata*	.	.	.	.	.	.	.	.	.	.	.	.	.	.	2	2	.	2	2	2	2	.	.	.	.	.	.	.	.	.
*Asphodelus ramosus*	+	.	.	.	r	.	.	.	3	2	2	.	.	.	.	.	.	.	.	.	.	.	.	.	.	.	.	.	.	.
*Ornithopus compressus*	.	.	.	.	.	.	.	.	.	.	.	.	.	.	.	.	.	1	1	+	1	.	.	.	.	.	.	.	.	.
*Aira caryophyllea*	.	.	.	.	.	.	.	.	.	2	.	.	.	.	.	2	.	.	+	.	1	.	.	.	.	.	.	.	.	.
*Sagina apetala*	.	.	.	.	.	.	.	.	.	.	.	.	.	.	.	.	1	2	+	.	.	.	.	.	.	.	.	.	r	.
*Medicago minima*	.	.	.	.	.	.	.	.	.	.	.	.	.	.	1	2	.	2	3	.	.	.	.	.	.	.	.	.	.	.
*Gastridium ventricosum*	.	1	.	.	+	.	.	+	.	.	.	.	.	.	.	.	.	.	.	.	.	.	.	.	.	.	.	.	.	.
*Parentucellia latifolia*	.	.	.	.	.	.	.	.	.	.	.	.	.	.	.	.	2	2	.	.	.	.	.	.	.	.	.	+	.	.
*Pulicaria odora*	.	.	.	.	.	.	.	.	.	.	.	.	.	.	2	2	.	2	.	.	.	.	.	.	.	.	.	.	.	.
*Plantago coronopus*	.	.	.	.	.	.	.	.	.	.	.	.	.	.	.	.	.	.	.	.	.	.	.	.	.	2	+	.	+	.
*Ornithogalum corsicum*	.	.	.	.	.	.	.	.	.	.	.	.	.	.	+	+	.	1	.	.	.	.	.	.	.	.	.	.	.	.
*Tuberaria lignosa*	.	.	.	.	.	.	.	.	.	.	.	.	.	.	.	.	.	.	.	.	.	r	.	.	.	+	.	.	r	.
*Carex divisa*	.	.	.	.	.	.	.	.	+	+	.	.	.	.	.	.	.	.	.	.	+	.	.	.	.	.	.	.	.	.
*Gaudinia fragilis*	.	.	.	.	.	.	.	.	1	.	.	+	.	.	.	.	.	.	.	.	.	.	.	.	.	.	.	.	.	.
*Paronychia echinulata*	.	.	.	.	.	.	.	.	.	.	.	.	.	.	2	2	.	.	.	.	.	.	.	.	.	.	.	.	.	.
*Avena barbata*	.	.	.	.	.	.	.	.	.	.	.	.	.	1	.	1	.	.	.	.	.	.	.	.	.	.	.	.	.	.
*Linum trigynum*	.	.	.	.	.	.	.	.	.	.	.	.	.	.	1	1	.	.	.	.	.	.	.	.	.	.	.	.	.	.
*Montia hallii*	.	.	.	.	.	.	.	.	.	.	.	.	.	.	.	.	+	1	.	.	.	.	.	.	.	.	.	.	.	.
*Ophrys fusca*	.	.	.	.	.	.	.	.	.	.	.	.	.	.	1	1	.	.	.	.	.	.	.	.	.	.	.	.	.	.
*Sedum caeruleum*	.	.	.	.	.	.	.	.	.	.	.	.	.	.	+	.	.	+	.	.	.	.	.	.	.	.	.	.	.	.
*Galactites tomentosus*	.	.	.	.	.	.	.	.	.	.	.	.	.	.	.	.	.	.	.	1	1	.	.	.	.	.	.	.	.	.
*Galium verrucosum*	.	.	.	.	.	.	.	.	.	.	.	.	.	.	+	+	.	.	.	.	.	.	.	.	.	.	.	.	.	.
*Hainardia cylindrica*	.	.	.	.	.	.	.	.	.	.	.	.	1	1	.	.	.	.	.	.	.	.	.	.	.	.	.	.	.	.
*Hypochoeris glabra*	.	.	.	.	.	.	.	.	.	.	.	.	.	.	.	.	1	.	+	.	.	.	.	.	.	.	.	.	.	.
*Teesdalia coronopifolia*	.	.	.	.	.	.	.	.	.	.	.	.	.	.	.	.	.	.	.	1	+	.	.	.	.	.	.	.	.	.
*Trifolium arvense*	.	.	.	.	.	.	.	.	.	.	.	.	.	.	1	.	.	.	1	.	.	.	.	.	.	.	.	.	.	.
*Vulpia ligustica*	.	.	.	.	.	.	.	.	.	.	.	.	.	.	.	.	.	.	1	.	+	.	.	.	.	.	.	.	.	.
*Lolium rigidum*	.	.	.	.	.	.	.	.	+	.	.	r	.	.	.	.	.	.	.	.	.	.	.	.	.	.	.	.	.	.
*Taeniatherum caput-medusae*	.	.	.	.	.	.	.	.	.	+	.	r	.	.	.	.	.	.	.	.	.	.	.	.	.	.	.	.	.	.
*Phalaris coerulescens*	.	.	.	.	.	.	.	.	.	.	.	3	.	.	.	.	.	.	.	.	.	.	.	.	.	.	.	.	.	.
*Aira caryophyllea*	.	.	.	.	.	.	.	.	.	.	.	+	.	.	.	.	.	.	.	.	.	.	.	.	.	.	.	.	.	.
*Ranunculus paludosus*	.	.	.	.	.	.	.	.	.	.	+	.	.	.	.	.	.	.	.	.	.	.	.	.	.	.	.	.	.	.
*Trifolium campestre*	.	.	.	.	.	.	.	.	.	.	.	.	.	.	.	.	.	.	1	.	.	.	.	.	.	.	.	.	.	.
*Vulpia geniculate*	.	.	.	.	.	.	.	.	.	.	.	.	.	.	.	2	.	.	.	.	.	.	.	.	.	.	.	.	.	.
*Morisia monanthos*	.	.	.	.	.	.	.	.	.	.	.	.	.	.	.	.	1	.	.	.	.	.	.	.	.	.	.	.	.	.
*Moenchia erecta*	.	.	.	.	.	.	.	.	.	.	.	.	.	.	1	.	.	.	.	.	.	.	.	.	.	.	.	.	.	.
*Orchis papilionacea*	.	.	.	.	.	.	.	.	.	.	.	.	.	.	.	.	.	+	.	.	.	.	.	.	.	.	.	.	.	.
*Ornithopus pinnatus*	.	.	.	.	.	.	.	.	.	.	.	.	.	.	.	.	.	.	.	.	+	.	.	.	.	.	.	.	.	.
*Leontodon tuberosus*	.	.	.	.	.	.	.	.	.	.	.	.	.	.	2	.	.	.	.	.	.	.	.	.	.	.	.	.	.	.
*Hordeum hystrix*	.	.	.	.	.	.	.	.	.	.	.	.	.	.	.	.	.	.	+	.	.	.	.	.	.	.	.	.	.	.
*Cerastium palustre*	.	.	.	.	.	.	.	.	.	.	.	.	.	.	.	.	.	+	.	.	.	.	.	.	.	.	.	.	.	.
*Dactylis hispanica*	.	.	.	.	.	.	.	.	.	.	.	.	.	.	.	.	.	.	+	.	.	.	.	.	.	.	.	.	.	.
*Ranunculus macrophyllus*	.	.	.	.	.	.	.	.	.	.	.	.	.	.	.	+	.	.	.	.	.	.	.	.	.	.	.	.	.	.
*Callitriche stagnalis*	.	.	.	.	.	.	.	.	.	.	.	.	.	.	.	.	.	.	.	.	.	.	.	.	.	r	.	.	.	.
*Spergula arvensis*	.	.	.	.	.	.	.	.	.	.	.	.	.	.	.	.	.	.	.	.	.	.	.	.	.	.	r	.	.	.

**Localities and dates of relevés**: Rel. 30–37, Olmedo (NW Sardinia), Bagella et al. (2009), Tab. 8; Ril. 38–41 Mogoro (Alta Marmilla) Caria et al. (2021), Tab.2; Rel. 42–43, Monte Minerva 17 March 2007; Rel. 44–45 Suni 7 April 2007; Rel. 46–48 Scanu Montiferru 7 April 2007; Rel. 49–50 Paule Longa (Paulilatino 7 April 2007; Rel. 51–59, Valverde (Alghero), Rivieccio et al., (2022b), Tab.7.

Characteristic species: *Isoëtes histrix*, *Romulea requenii*.

Structure and ecology: This association occurs along the margins of the large lagoons localized in inland places on volcanic substrates, in most cases of ancient origin, where it is linked mainly to stands with shallow waters that dry up from late spring. It is characterized by *Isoëtes histrix*, which usually grows with *Romulea requienii*, a Sardinian-Corsican endemism, or more rarely with *Isoëtes gymnocarpa*. Other hygrophytes of the *Isoëto-Nanojuncetea* frequent in this association are *Lythrum hyssopifolia*, *Lotus hispidus*, *Agathryon bufonium*, *Centaurium maritimum*, *Agrostis pourretii*, *Juncinella capitata*, *Verojuncus pygmaeus*, *Isolepis cernua*, *Ranunculus ophioglossifolius*, *Ophioglossum lusitanicum*, *Cicendia filiformis*, *Solenopsis laurentia* subsp. *laurentia*, etc. The relevés carried out in North-West Sardinia by [23] referred to the *Bellidi annuae-Cicendietum filiformis* de Foucault 1988, can be considered as an impoverished aspect in characteristic species of the *Romuleo requieni-Isoëtetum histricis*. Therefore, the occurrence of the association described by [58] must be excluded from Sardinia.

Geographical distribution: The association was surveyed in North-West and Central-Western Sardinia as recorded by [23,27,28], as well as from several unpublished relevés.

*AGROSTION POURRETII* Rivas Goday 1958 Anal. Inst. Bot. Cavanil1es 14: 513 nom. mut.

Syn.: *Pre-Isoëtion* Rivas Goday 1956, Anal. Inst. Bot. Cavanilles 13: 340 nom. inval. (art. 3b); *Agrostion salmanticae* Rivas Goday 1956, Anal. Inst. Bot. Cavanilles 13: 387, nom. nud. (art. 2b); *Agrostion salmanticae* Rivas Goday 1958, Anal. Inst. Bot. Cavanilles 14: 513, nom. inept. (art. 45).

Lectotypus: As. *Pulicaria uliginosa et Agrostis salmantica* Rivas Goday in Rivas Goday et al., 1956, Anal. Inst. Bot. Cavanilles 13: 386.

Characteristic species: *Agrostis pourretii*, *Chamaemelum fuscatum*, *Cynosurus polybracteatus*, *Hordeum geniculatum*, *Trifolium michelianum*.

Structure and ecology: The alliance includes associations with a spring cycle linked to wet depressions flooded by long-persistent rain waters during the winter and spring. This vegetation occurs mainly on markedly arenaceous soils and is physiognomically characterized by the dominance of graminoid therophytes, showing its optimum towards late spring. The plant communities of this alliance can be considered with intermediate ecological requirements between the markedly hygrophilous associations of the *Menthion cervinae* and the ephemeral xerophilous meadows of the *Tuberarion guttatae*. It was initially described as *Pre-Isoëtion* all provided by [59], who also proposed, as an alternative name, *Agrostion salmanticae* nom. nud., syntaxon later validated by the same author [60]. Based on art. 45 of the ICPN, refs. [52,61] independently proposed to change the name of this alliance to *Agrostion pourretii* nom. mut. In Sardinia, this alliance is floristically differentiated mainly by the dominance of *Agrostis pourretii*, while the other species of this syntaxon are rarer or more absent than the plant communities occurring in the Iberian Peninsula. Therefore, the associations of this alliance occurring in Sardinia do not differ very much from those of the *Isoëtion*.

Geographical distribution: The alliance is surveyed in the Iberian-Atlantic and West Mediterranean territories (Spain, France, Corse, Sardinia, Sicily, and southern Italy).

#### 2.3.6. *Antoxantho aristati-Agrostietum pourretii* Biondi & Bagella 2005, Fitosociologia 42 (2) Suppl. 1: 20 (Table 7)

Holotypus: rel. 7, tab. 19 [21].

**Table 7:** *Anthoxantho aristati-Agrostietum salmanticae* Biondi & Bagella 2005.

Relevè Number	1	2	3	4	5	6	7	8	9	10	11	12	13	14	15	16	17	18	19	20	21	22	23	24	25	26	27	28	29	30	31	32	33
Altitude (dam)	1	1	1	20	20	20	20	20	20	20	20	20	20	20	20	20	20	20	-	-	-	-	-	-	-	-	-	-	-	-	-	-	-
Surface (m^2^)	3	3	4	10	10	6	6	6	4	6	4	8	4	4	4	5	4	6	50	20	3	2	3	10	2	5	5	50	70	30	40	15	20
Coverage (%)	100	100	100	100	100	100	100	100	100	100	100	100	100	100	100	100	90	100	100	100	100	100	100	100	100	100	90	100	100	100	100	100	100
** Char. Association**																																	
*Anthoxanthum aristatum*	1	2	1	+	+	.	+	1	1	+	.	1	+	.	.	1	.	.	2	+	2	.	2	2	.	1	.	3	4	+	4	4	.
*Gaudinia fragilis*	2	2	2	+	+	1	+	1	1	+	+	1	1	1	1	1	.	1	.	2	.	+	.	.	+	1	+	1	2	4	2	.	3
** Char. *Agrostion pourretii***																																	
*Agrostis pourretii*	4	3	4	3	2	4	4	3	3	2	3	4	4	4	4	4	4	4	3	5	5	5	5	5	3	4	4	5	4	1	1	3	2
*Hordeum geniculatum*	.	.	.	1	1	.	+	1	3	4	3	+	+	+	+	+	3	4	3	.	.	.	.	.	.	.	.	.	.	.	.	.	.
*Cynosurus polybracteatus*	.	.	.	.	.	r	+	1	.	.	.	.	r	.	+	.	r	.	.	.	.	.	.	.	.	.	.	.	.	.	.	.	.
*Trifolium michelianum*	.	.	.	+	.	.	.	+	+	r	.	.	.	.	.	.	.	.	.	.	.	.	.	.	.	.	.	.	.	.	.	.	.
** Char.** ***Isoë*** ***tetalia* and ** ***Isoë*** ***to-Nanojuncetea***																																	
*Lythrum hyssopifolia*	+	1	1	+	.	r	1	1	1	1	+	+	+	+	1	1	1	1	1	.	.	+	.	.	.	.	.	.	.	.	.	.	.
*Mentha pulegium*	2	1	1	.	.	.	r	.	+	.	.	.	+	1	1	+	+	.	+	.	.	.	+	.	2	.	1	.	.	.	.	.	.
*Ranunculus cordiger* ssp. *diffusus*	3	3	2	1	2	.	.	1	2	1	.	.	.	1	+	.	+	.	.	.	.	.	.	.	.	.	.	.	.	.	.	.	.
*Lotus hispidus*	1	2	+	.	.	.	.	.	.	.	.	2	1	1	4	3	+	3	.	.	.	.	.	.	.	.	.	.	.	.	.	.	.
*Eudianthe laeta*	2	2	2	.	.	.	+	.	.	.	.	.	.	.	.	.	.	.	2	.	.	.	.	.	.	.	.	.	2	.	.	.	.
*Eryngium pusillum*	.	.	.	.	.	.	r	.	.	.	.	.	.	1	1	+	r	+	.	.	.	.	.	.	.	.	.	.	.	.	.	.	.
*Agathryon bufonium*	.	.	.	.	.	.	.	2	.	.	.	1	.	.	.	r	1	1	.	.	.	.	.	+	.	.	.	.	.	.	.	.	.
*Polypogon subspathaceus*	1	2	1	.	.	.	.	.	.	.	.	.	.	.	.	.	.	.	.	.	.	.	.	.	+	+	.	.	.	.	.	.	.
*Lotus angustissimus*	.	.	.	.	.	.	.	.	.	.	.	.	.	.	.	.	.	.	.	+	.	+	.	1	.	+	+	.	.	.	.	.	.
*Verojuncus pygmaeus*	.	.	.	.	.	.	1	+	.	.	.	.	.	.	.	.	+	.	.	.	.	2	.	.	.	.	.	.	.	.	.	.	.
*Briza minor*	1	+	2	.	.	.	.	.	.	.	.	.	.	.	.	.	.	.	.	.	.	.	.	+	.	.	.	.	.	.	.	.	.
*Solenopsis laurentia*	.	.	.	+	+	.	.	.	r	1	.	.	.	.	.	.	.	.	.	.	.	.	.	.	.	.	.	.	.	.	.	.	.
*Centaurium maritimum*	.	.	.	.	.	.	.	+	r	.	.	.	.	.	.	.	.	.	.	.	.	+	.	.	.	.	.	.	.	.	.	.	.
*Illecebrum verticillatum*	.	.	.	+	+	.	.	.	.	+	.	.	.	.	.	.	.	.	.	.	.	.	.	.	.	.	.	.	.	.	.	.	.
*Isolepis cernua*	.	+	+	.	.	.	.	+	.	.	.	.	.	.	.	.	.	.	.	.	.	.	.	.	.	.	.	.	.	.	.	.	.
*Trifolium micranthum*	1	2	1	.	.	.	.	.	.	.	.	.	.	.	.	.	.	.	.	.	.	.	.	.	.	.	.	.	.	.	.	.	.
*Agathryon hybridum*	+	1	1	.	.	.	.	.	.	.	.	.	.	.	.	.	.	.	.	.	.	.	.	.	.	.	.	.	.	.	.	.	.
*Anagallis parviflora*	+	.	+	.	.	.	.	.	.	.	.	.	.	.	.	.	.	.	.	.	.	.	.	.	.	.	.	.	.	.	.	.	.
*Middendorfia borysthenica*	.	.	.	.	.	.	.	.	+	r	.	.	.	.	.	.	.	.	.	.	.	.	.	.	.	.	.	.	.	.	.	.	.
*Myosotis sicula*	.	.	.	+	.	.	.	r	.	.	.	.	.	.	.	.	.	.	.	.	.	.	.	.	.	.	.	.	.	.	.	.	.
*Ranunculus sardous*	.	.	.	.	.	.	.	.	.	.	.	.	.	.	.	.	.	.	2	.	.	+	.	.	.	.	.	.	.	.	.	.	.
*Juncinella capitata*	.	.	.	.	.	.	.	.	.	.	.	.	.	.	.	.	.	.	.	.	.	.	.	.	+	.	.	.	.	.	.	.	.
*Kickxia cirrhosa*	.	.	.	.	.	.	.	.	.	.	.	.	.	.	.	.	.	.	.	.	.	+	.	.	.	.	.	.	.	.	.	.	.
*Agathryon tenageia*	.	.	.	.	.	.	.	.	.	r	.	.	.	.	.	.	.	.	.	.	.	.	.	.	.	.	.	.	.	.	.	.	.
** Other species**																																	
*Polypogon maritimus*	.	.	.	3	1	2	2	.	1	+	.	3	.	+	+	+	2	+	.	.	.	.	.	.	.	.	.	.	.	.	.	.	.
*Trifolium resupinatum*	.	+	1	.	.	1	.	.	1	.	.	3	2	3	3	4	2	1	.	.	.	.	.	.	.	.	.	.	.	.	.	.	.
*Avena barbata*	.	.	.	.	.	.	.	.	.	.	.	.	.	.	.	.	.	.	.	1	+	.	+	+	.	+	.	1	1	2	1	1	1
*Plantago lanceolata*	.	.	.	.	.	.	.	.	.	.	.	.	.	.	.	.	.	.	+	1	1	+	1	.	.	.	.	1	1	2	+	+	.
*Echium plantagineum*	.	.	.	.	.	.	.	.	.	.	.	.	.	.	.	.	.	.	.	.	+	+	.	+	.	+	.	1	2	1	1	+	.
*Lolium multiflorum*	.	.	.	.	.	.	.	.	+	.	.	+	r	+	+	+	+	+	.	.	.	.	.	.	.	.	.	.	.	.	.	.	.
*Bromus hordeaceus*	.	.	.	.	.	+	+	.	.	.	+	1	.	.	.	.	.	.	1	.	.	.	.	+	.	.	.	.	+	.	.	.	2
*Plantago weldenii*	.	.	.	.	.	1	.	.	+	.	.	.	.	.	.	+	+	.	.	+	.	1	.	.	+	.	+	.	.	.	.	.	.
*Linum bienne*	.	.	.	.	.	.	.	.	.	+	.	.	.	.	.	+	.	.	.	.	.	.	.	1	.	.	.	+	2	+	+	3	.
*Cynosurus echinatus*	.	.	.	.	.	.	.	.	.	.	.	.	.	.	.	.	.	.	.	.	1	.	.	.	.	.	.	2	2	3	3	3	1
*Macrobriza maxima*	.	.	.	.	.	.	.	.	.	.	.	.	.	.	.	.	.	.	.	.	+	.	.	.	.	+	.	+	1	1	1	1	.
*Trifolium campestre*	.	+	.	.	.	.	.	.	.	.	.	.	.	.	.	.	.	.	.	.	+	.	.	.	.	.	.	+	.	2	2	1	+
*Medicago polymorpha*	.	.	.	.	.	.	.	.	.	.	.	.	.	.	.	.	.	.	.	.	.	.	.	.	.	.	.	2	2	1	1	1	1
*Bellardia viscosa*	.	.	.	.	.	.	.	.	.	.	.	.	.	.	.	.	.	.	.	.	+	.	.	.	.	.	.	+	1	1	1	.	+
*Lotus pedunculatus*	.	.	.	4	3	3	3	3	3	4	4	4	.	.	.	.	.	.	.	.	.	.	.	.	.	.	.	.	.	.	.	.	.
*Alopecurus bulbosus*	.	.	.	.	.	.	.	.	+	+	.	.	.	+	.	+	r	.	.	.	.	.	.	.	.	.	.	.	.	.	.	.	.
*Bellis annua*	.	.	.	+	+	.	.	.	+	+	.	.	.	r	.	.	.	.	.	.	.	.	.	.	.	.	.	.	.	.	.	.	.
*Lolium rigidum*	.	.	.	.	.	.	.	.	.	.	.	.	.	.	.	.	.	.	.	.	.	.	.	.	.	1	+	+	+	.	.	.	3
*Cynodon dactylon*	.	.	.	.	.	.	.	.	.	.	.	.	.	.	.	.	.	.	.	2	2	.	.	.	1	1	3	.	.	.	.	.	.
*Rumex obtusifolius*	.	.	.	.	.	.	.	.	.	.	.	.	.	.	.	.	.	.	.	.	.	.	.	.	.	.	.	+	.	+	+	+	2
*Anthemis arvensis*	.	.	.	.	.	+	.	.	.	.	.	r	+	.	.	.	+	.	.	.	.	.	.	.	.	.	.	.	.	.	.	.	.
*Serapias lingua*	.	.	.	.	.	+	+	r	r	.	.	.	.	.	.	.	.	.	.	.	.	.	.	.	.	.	.	.	.	.	.	.	.
*Linum strictum*	.	.	.	.	.	.	.	.	.	.	.	.	.	.	.	.	.	.	.	.	+	+	.	+	.	+	.	.	.	.	.	.	.
*Echium vulgare*	.	.	.	.	.	+	.	.	.	.	.	+	.	.	.	.	+	.	.	.	.	.	.	.	.	.	.	.	.	.	.	.	.
*Carex divisa*	.	.	.	.	.	.	.	.	.	.	.	.	.	.	+	+	.	.	1	.	.	.	.	.	.	.	.	.	.	.	.	.	.
*Trifolium nigrescens*	.	.	.	.	.	.	.	.	.	.	.	.	.	.	.	.	.	.	.	.	.	.	.	.	.	.	.	1	.	1	1	.	.
*Coleostephus myconis*	1	2	1	.	.	.	.	.	.	.	.	.	.	.	.	.	.	.	.	.	.	.	.	.	.	.	.	.	.	.	.	.	.
*Plantago coronopus*	1	1	2	.	.	.	.	.	.	.	.	.	.	.	.	.	.	.	.	.	.	.	.	.	.	.	.	.	.	.	.	.	.
*Cyperus badius*	1	1	+	.	.	.	.	.	.	.	.	.	.	.	.	.	.	.	.	.	.	.	.	.	.	.	.	.	.	.	.	.	.
*Trifolium arvense*	.	.	.	.	.	.	.	.	.	.	.	.	.	.	.	.	.	.	.	.	+	.	.	+	.	.	.	.	.	.	.	+	.
*Romulea requienii*	.	.	.	.	.	.	+	.	+	.	.	.	.	.	.	.	.	.	.	.	.	.	.	.	.	.	.	.	.	.	.	.	.
*Hedypnois cretica*	.	.	.	.	.	.	.	.	.	.	.	.	.	.	.	.	.	.	.	.	.	.	.	.	.	.	.	+	+	.	.	.	.
*Raphanus raphanistrum*	.	.	.	.	.	.	.	.	.	.	.	.	.	.	.	.	.	.	.	.	.	.	.	.	.	.	.	.	+	.	.	+	.
*Sherardia arvensis*	.	.	.	.	.	.	.	.	.	.	.	.	.	.	.	.	.	.	.	.	.	.	.	.	.	.	.	+	+	.	.	.	.
*Vicia sativa*	.	.	.	.	.	.	.	.	.	.	.	.	.	.	.	.	.	.	.	.	.	.	.	.	.	.	.	.	.	+	.	.	+
*Anagallis foemina*	.	.	.	.	.	.	.	.	.	.	.	.	.	.	.	.	.	.	.	.	+	+	.	.	.	.	.	.	.	.	.	.	.
*Cerastium glomeratum*	.	.	.	.	.	.	.	.	.	.	.	.	.	.	.	.	.	.	.	.	.	.	+	+	.	.	.	.	.	.	.	.	.
*Holcus lanatus*	.	.	.	.	.	.	.	.	.	.	.	.	.	.	.	.	.	.	.	.	.	.	.	.	.	.	.	.	.	.	+	.	2
*Hypochoeris glabra*	.	.	.	.	.	.	.	.	.	.	.	.	.	.	.	.	.	.	.	.	.	+	.	.	+	.	.	.	.	.	.	.	.
*Lagurus ovatus*	.	.	.	.	.	.	.	.	.	.	.	.	.	.	.	.	.	.	.	.	+	.	.	+	.	.	.	.	.	.	.	.	.
*Silene gallica*	.	.	.	.	.	.	.	.	.	.	.	.	.	.	.	.	.	.	.	.	.	.	.	.	+	+	.	.	.	.	.	.	.
*Vulpia myuros*	.	.	.	.	.	.	.	.	.	.	.	.	.	.	.	.	.	.	.	.	.	+	+	.	.	.	.	.	.	.	.	.	.
*Hordeum marinum*	.	.	.	.	.	.	.	.	.	.	.	.	.	.	.	.	.	.	.	.	.	.	.	.	.	.	.	.	.	.	.	.	2
*Paspalun distichum*	.	.	.	.	.	.	.	.	.	.	.	.	.	.	.	.	.	.	1	.	.	.	.	.	.	.	.	.	.	.	.	.	.
*Lolium perenne*	.	.	.	.	.	.	.	.	.	.	.	.	.	.	.	.	.	.	2	.	.	.	.	.	.	.	.	.	.	.	.	.	.

**Localities and dates of relevés**: Rel. 1–3 Baia del Sole (Olbia), 21 June 1996; Rel. 4–18, Olmedo (NW Sardinia), Bagella et al. (2009), Tab. 9; Rel.19: Asinara Island, Pisanu et al. (2014), Tab. 3. rel 3; Rel. 20–33, La Maddalena Archipelago, Biondi and Bagella (2005) Tab. 19.

Characteristic species: *Anthoxanthum aristatum*, *Gaudinia fragilis*.

Structure and ecology: This association occurs in wetlands, represented by more or less large depressions with sandy or incoherent soils. It is dominated by *Agrostis pourretii* (=*A. salmantica*), an annual caespitose grass distributed in the western Mediterranean territories. It is localized mainly in coastal or hilly environments, limitedly to habitats affected during winter–spring by long periods of submersion. In addition to the aforementioned species, numerous other hygrophytes grow, such as *Anthoxanthum aristatum*, *Gaudinia fragilis*, *Hordeum geniculatum*, *Lythrum hyssopifolia*, *Mentha pulegium*, *Ranunculus cordiger* subsp. *diffusus*, *Lotus hispidus*, etc. For its floristic set and ecology, the association is to be included in the *Agrostion salmanticae* alliance, currently known for several countries of the western Mediterranean [15]. Previously, other associations of this alliance were mentioned for Italian territory, such as *Trifolio micheliani-Agrostidetum pourretii* Cambria & Brullo in Brullo et al., 2022 from Sicily [15] and *Phalarido minoris-Agrostidetum pourretii* Tomaselli et al., 2020 from Apulia [50].

Geographical distribution: This vegetation, already recorded in North Sardinia by [21,23,24], was found in “Baia del Sole” near Olbia too.

#### 2.3.7. *Verojunco tingitani-Agrostietum pourretii* Brullo, Bacch., Giusso & Miniss. ass. nov. (Table 8)

Holotypus: rel. 1, tab. 8.

**Table 8:** *Verojunco tingitani-Agrostietum pourretii* Brullo et al. ass.nov.

Relevè Number	1 *	2	3	4	5	6	7 *	8	9	10	11
Altitude (m)	670	670	670	670	670	5	5	5	5	5	5
Surface (m^2^)	1	1	1	1	1	4	3	3	2	4	2
Coverage (%)	90	90	90	90	80	100	100	100	100	100	100
** Char. Association**											
*Verojuncus tingitanus*	3	3	4	3	2	2	2	2	2	1	2
** Char. Subassociation**											
*Kickxia cirrhosa*	.	.	.	.	.	2	3	2	2	1	2
*Anagallis parviflora*	.	.	.	.	.	2	2	1	2	1	1
*Solenopsis laurentia* subsp. *laurentia*	.	.	.	.	.	1	2	2	1	2	2
*Cicendia filiformis*	.	.	.	.	.	+	1	1	1	+	1
*Eudianthe laeta*	.	.	.	.	.	+	+	.	1	1	1
*Radiola linoides*	.	.	.	.	.	+	+	.	+	1	+
*Buillardia vaillantii*	3	2	3	3	2	.	.	.	.	.	.
** Char. *Agrostion pourretii***											
*Agrostis pourretii*	3	2	+	2	1	4	3	3	4	4	3
*Chamaemelum fuscatum*	.	.	.	.	.	1	1	1	+	.	1
*Hordeum geniculatum*	+	+	+	.	.	.	.	.	.	.	.
** Char.** ***Isoë*** ***tetalia***											
*Isoët* *es durieui*	1	2	1	+	.	3	3	4	2	3	4
*Isoët* *es histrix*	.	.	.	.	.	1	1	+	1	2	1
*Exaculum pusillum*	+	+	.	+	.	.	.	+	.	+	.
*Romulea ramiflora*	1	2	+	+	.	.	.	.	.	.	.
*Trifolium micranthum*	1	+	.	.	1	.	.	.	.	.	.
*Lotus parviflorus*	1	.	+	.	.	.	.	.	.	.	.
*Illecebrum verticillatum*	+	.	.	.	.	.	.	.	.	.	.
** Char.** ***Isoë*** ***to-Nanojuncetea***											
*Agathryon bufonium*	2	1	+	+	1	1	2	1	1	2	+
*Lythrum hyssopifolia*	1	2	1	1	1	2	1	2	2	1	2
*Polypogon subspathaceus*	2	2	1	+	+	+	+	.	+	+	.
*Lotus hispidus*	.	.	.	.	.	1	1	1	2	2	1
*Mentha pulegium*	1	1	+	1	+	.	.	.	.	.	.
*Poa infirma*	+	+	+	+	1	.	.	.	.	.	.
*Peplis portula*	2	3	2	2	+	.	.	.	.	.	.
*Isolepis cernua*	.	.	.	.	.	+	1	1	+	.	+
*Ranunculus revelieri*	1	1	+	+	.	.	.	.	.	.	.
*Corrigiola litoralis*	.	.	.	.	.	+	.	+	+	.	.
*Briza minor*	.	.	.	.	.	+	.	+	+	.	.
*Centaurium maritimum*	.	.	.	.	.	.	.	+	.	+	.
*Pulicaria vulgaris*	.	.	.	2	.	.	.	.	.	.	.
** Other species**											
*Coleostephus myconis*	.	.	.	.	.	+	2	1	1	2	1
*Bellis annua*	1	+	+	+	+	.	.	.	.	.	.
*Lotus* sp.	2	2	+	.	1	.	.	.	.	.	.
*Prospero autumnalis*	.	.	.	.	.	+	.	+	+	.	.
*Reseda luteola*	.	.	.	.	.	+	.	+	.	.	.
*Trifolium campestre*	.	.	.	.	.	+	.	.	.	.	.
*Linum bienne*	.	.	.	.	.	+	.	.	.	.	.
*Bromus hordeaceus*	.	.	.	.	.	+	.	.	.	.	.
*Spergularia rubra*	.	.	.	.	.	+	.	.	.	.	.
*Sedum glandulosum*	.	.	.	.	2	.	.	.	.	.	.
*Sedum caeruleum*	.	.	.	.	1	.	.	.	.	.	.
*Sedum andegavense*	.	.	.	.	2	.	.	.	.	.	.

**Localities and dates of relevés**: Rel. 1–5, Monte Cardiga, 26 May 2002; Rel 6–11, Baia del Sole (Olbia), 21 June 1996. The symbol (*) indicates the nomenclatural type as specified in the ICPN code.

Characteristic species: *Verojuncus tingitanus*, *Agrostis pourretii*.

Structure and ecology: Another plant community characterized by the dominance of *Agrostis pourretii* and clearly referable to *Agrostion pourretii*, which, in its typical aspect, was surveyed on the top of a limestone plateau. It differs from the *Antoxantho aristati-Agrostietum pourretii* for the occurrence of *Verojuncus tingitanus* (=*Juncus tingitanus*), a species quite rare in Sardinia, and for the absence of *Anthoxanthum aristatum* and *Gaudinia fragilis.* Ecologically, this vegetation is localized in wide depressions submerged by rainwater until late spring with damp carbonatic soil. Floristically, it is differentiated by a rich set of hygrophytes of the *Isoetetalia* and *Isoeto-Nanojuncetea* such as *Isoetes durieui*, *Exaculum pusillum*, *Romulea ramiflora*, *Trifolium micranthum*, *Agathyron bufonium*, *Lythrum hyssopifolia*, *Polypogon subspathaceus*, *Mentha pulegium*, *Poa infirma*, *Peplis portula*, etc. It is therefore described as a new association, namely *Verojunco tingitani-Agrostietum pourretii.* Based on field investigations, the association was also observed in coastal stands represented by wet depressions on sandy-loamy substrates, where, however, the floral set is enriched with microphytes of the *Cicendio-Solenopsion laurentiae*, including in particular *Anagallis parviflora*, *Cicendia filiformis*, *Solenopsis laurentia* subsp. *laurentia*, *Kickxia cirrhosa*, *Silene laeta* and *Radiola linoides*. From a syntaxonomical point of view, within this association can be recognized two different subassociations, which are proposed as *Bulliardetosum vaillantii* Brullo et al. subass. nov., as concerns that one detected in the inland stands, having *Bulliarda vaillantii* as a differential species, which must be considered as the typical subassociation, while the coastal one is proposed as *Kickxietosum cirrhosae* Brullo et al. subass. nov. which is differentiated by *Kickxia cirrhosa* (holotypus: rel. 7).

Geographical distribution: The association as regards the subass. *Bulliardetosum vaillantii*, occurs at ca. 600 m a.s.l., on the Monte Cardiga plateau near Villaputzu, while the subass. *Kickxietosum cirrhosae* was observed in some depressions of Baia del Sole near Olbia at sea level.

*MENTHION CERVINAE* Br.-Bl. ex Moor 1937, Prodr. Group. Veg. 4: 22 nom. mut.

Syn.: *Preslion* Br.-Bl. 1931, Comm. S.I.G.M.A.: 38, nom. nud. (art. 2b); Preslion *cervinae* Br.-Bl. ex Moor 1937, Prodr. Group. Veg. 4: 22, nom. inept. (art. 45); *Elatino-Damasonion alismae* de FoucauIt 1988, Dissert. Bot. 121: 86, p.p.

Holotypus: *Menthetum cervinae* Br.-Bl. ex Moor 1937 nom mut.

Characteristic species: *Antinoria insularis*, *Callitriche brutia*, *Ranunculus lateriflorus*, *R. ophioglossifolius*, *R. saniculifolius*.

Structure and ecology: These thermophilous plant communities included in this alliance occur in cupular pools, temporary marshes, and dolines with deep stagnant waters or in stands with deep-water runoff flooded for most of the spring. This vegetation is rich in creeping amphibian species interspersed with other microphytes. This alliance was originally described by Braun-Blanquet [3] as *Preslion cervinae*, but [52,61] independently proposed to change the name of this alliance to *Menthion cervinae* nom. mut., since in accordance with art. 45 of the code, it is *nomen ineptum.*

Geographical distribution: This alliance seems to have a Mediterranean range.

*APIENION CRASSIPEDIS* Bagella et al., 2009, Fitosociologia 46 (1): 24.

Holotypus: *Apio crassipedis-Isoetetum tigulianae* Biondi & Bagella 2005.

Characteristic species: *Helosciadium crassipes*, *Isoëtes tiguliana*, *Ranunculus cordiger* subsp. *diffusus*, *R. revelieri*.

Structure and ecology: This suballiance groups the plant communities linked to stands that remain submerged by relatively deep rainwater for extended periods during winter and spring. It can be considered as a local vicariant of the *Menthion cervinae*, being differentiated floristically from a set of endemic species.

Geographical distribution: According to [23], some of the species proposed as characteristic are also distributed outside the Cyrno-Sardinian territories. This syntaxon seems to have a Tyrrhenian distribution, but it probably occurs especially in Sardinia and Corsica.

#### 2.3.8. *Montio arvensis-Ranunculetum revelieri* Brullo, Bacch., Giusso & Miniss. ass. nov. (Table 9)

Holotypus: rel. 5, tab. 9.

**Table 9:** *Montio arvensis-Ranunculetum revelieri* Brullo et al. ass. nov.

Relevè Number	1	2	3	4	5 *	6	7	8	9	10	11	12	13	14 *	15	16	17	18	19
Altitude (m)	550	550	550	550	550	550	550	550	550	550	650	650	650	650	650	650	650	650	650
Surface (m^2^)	3	3	2	2	3	4	2	3	3	3	2	2	1	3	3	3	2	2	1
Coverage (%)	90	100	90	100	100	100	100	100	90	100	60	70	60	70	80	70	70	60	60
** Char. Association and *Apienion crassipedis***																			
*Ranunculus revelieri*	2	2	2	2	4	2	3	3	2	2	2	1	3	2	2	1	1	3	2
*Montia arvensis*	1	2	3	2	2	3	4	2	3	3	2	1	1	2	3	3	1	2	2
** Char.** ***Isoë*** ***tetalia***																			
*Ranunculus trilobus*	2	2	1	2	1	2	2	1	1	+	1	+	+	2	2	1	.	1	1
*Myosotis sicula*	3	4	3	4	2	3	1	2	1	1	.	1	.	2	2	2	2	1	1
*Lotus parviflorus*	.	.	.	.	.	.	+	+	.	.	1	2	1	1	1	1	1	2	1
*Trifolium micranthum*	.	.	.	.	.	.	.	.	.	1	1	2	2	1	+	1	.	1	+
*Isoët* *es durieui*	.	.	.	.	.	.	.	.	.	.	2	3	2	2	1	1	3	1	1
*Isoët* *es histrix*	.	.	.	.	.	.	.	.	.	.	.	.	.	+	1	2	1	3	2
*Isolepis cernua*	.	.	.	.	.	.	.	.	.	2	.	.	.	.	.	.	.	.	.
** Char.** ***Isoë*** ***to-Nanojuncetea***																			
*Agathryon bufonium*	2	1	+	+	1	1	2	2	1	+	+	.	1	+	2	2	1	.	2
*Mentha pulegium*	1	2	1	1	+	1	1	2	3	3	1	.	+	+	1	.	2	+	.
*Lythrum hyssopifolia*	.	.	.	.	.	.	.	.	.	.	1	1	+	1	+	1	2	+	1
*Poa infirma*	.	.	.	.	.	.	.	.	.	.	.	1	.	+	.	1	+	.	1
*Gaudinia fragilis*	.	.	.	.	.	.	.	.	.	.	.	.	.	1	+	.	.	1	1
*Corrigiola litoralis*	.	.	.	.	.	.	.	1	1	2	.	.	.	.	.	.	.	.	.
** Other species**																			
*Oenanthe lisae*	.	.	.	.	1	1	+	1	.	1	2	2	1	1	1	.	.	.	1
*Cerastium palustre*	.	+	+	.	+	.	.	.	.	.	.	.	+	1	1	1	1	1	.
*Alopecurus bulbosus*	1	3	2	2	1	1	2	1	+	+	.	.	.	.	.	.	.	.	.
*Coleostephus myconis*	.	+	1	+	.	.	1	.	.	1	.	.	.	.	.	.	.	.	.

**Localities and dates of relevés**: Rel. 1–10, Little lake between Monti and Cantoniera di Zuighe (Alà dei Sardi), 4 May 1995; Rel. 11–19, Plateau near Alà dei Sardi, 4 May 1995. The symbol (*) indicates the nomenclatural type as specified in the ICPN code.

Characteristic species: *Montia arvensis*, *Ranunculus revelieri*.

Structure and ecology: This vegetation was surveyed in wide temporary ponds flooded during winter–spring on granite substrata covered by muddy soils. It is localized in hilly and submountain places at 550–650 m a.s.l. and is differentiated by the occurrence of *Montia arvensis* and *Ranunculus revelieri*, hygrophytes colonizing stands with quite deep waters. These species are proposed as characteristics of a new association of *Apienion crassipedis*, namely *Montio arvensis-Ranunculetum revelieri.* They usually grow with a few other hygrophytes, such as *Ranunculus trilobus*, *Myosotis sicula*, *Agathryon bufonium,* and *Mentha pulegium.* Within this association, two well-distinct subassociations can be distinguished; the first is represented by *Alopecuretosum bulbosi* subass. nov. (holotypus: rel. 5), differentiated by the occurrence of *Alopecurus bulbosus* and localized on surfaces with waters deeper than 10 cm, while the second by *Isoetetosum durieui* subass. nov. (holotypus: rel. 14), differentiated by *Isoëtes durieui*, *I. histrix,* and *Trifolium micranthum*, which occurs in the stands with shallower waters.

Geographical distribution: The association is localized in some locality near Alà dei Sardi in North-East Sardinia.

#### 2.3.9. *Callitricho stagnalis-Isoëtetum longissimae* Bagella, Rivieccio & Caria ass. nov. (Table 10)

Holotypus: rel. 3, tab. 10.

**Table 10:** *Callitricho stagnalis-Isoëtetum longissimae* Bagella et al. ass. nov.

Relevè Number	1 *	2	3	4	5	6	7
Altitude (m)	72	72	72	72	309	309	309
Surface (m^2^)	8	6	4	1	6	4	8
Coverage (%)	90	80	100	80	80	90	90
** Char. Association**							
*Isoët* *es longissima*	4	3	2	3	3	1	3
** Char. *Menthion cervinae* and *Apienion crassipedis***							
*Callitriche stagnalis*	4	3	1	2	2	2	4
*Ranunculus cordiger* subsp. *diffusus*	r	.	.	.	.	.	.
** Char.** ***Isoë*** ***to-Nanojuncetea***							
*Middendorfia borysthenica*	+	+	2	2	.	1	.
*Chamaemelum fuscatum*	+	+	1	+	2	r	1
*Lythrum hyssopifolia*	+	+	1	+	1	+	+
*Agathryon bufonium*	.	.	+	+	+	.	.
*Ranunculus sardous*	.	+	+	.	.	.	.
*Eudianthe laeta*	.	.	+	.	.	.	R
*Lotus hispidus*	.	.	.	.	r	r	.
*Poa infirma*	.	.	.	.	+	.	.
*Verojuncus pygmaeus*	.	.	.	.	+	.	.
** Other species**							
*Glyceria plicata*	3	1	3	2	1	4	3
*Eleocharis palustris*	1	+	.	+	.	.	.
*Bellis annua*	.	.	.	r	+	.	+
*Linum bienne*	+	.	+	.	.	.	+
*Ranunculus aquatilis*	1	+	.	.	.	.	.
*Agathryon subulatum*	.	.	.	.	1	.	.

**Localities and dates of relevés**: Rel. 1–4, La Scaletta (Alghero), Rivieccio et al. (2022c), Tab. 4, rel. 1–4; Rel. 5–7, Scala Picada (Alghero) Rivieccio et al. (2022), Tab. 4, rel. 5–7. The symbol (*) indicates the nomenclatural type as specified in the ICPN code.

Characteristic species: *Isoëtes longissima*, *Callitriche stagnalis*.

Structure and ecology: This vegetation, dominated by *Callitriche stagnalis* and *Isoëtes longissima*, the latter sporadic in Sardinia, occurs in small ponds flooded during the winter–spring period on volcanic substrata, at an altitude of 200–300 m a.s.l. Previously, *Isoëtes longissima* was erroneously identified by [26] as *Isoëtes tiguliana*. Moreover, several species of the class *Isoëto-Nanojuncetea* and some aquatic species such as *Glyceria plicata* and *Ranunculus aquatilis* are also present. Therefore, based on its floristic and ecological peculiarities it is described as a new association, namely *Callitricho stagnalis-Isoëtetum longissimae*, referred to *Apienion crassipedis*.

Geographical distribution: The association was surveyed in North-West Sardinia near Villanova (Alghero) by [26].

#### 2.3.10. *Isoëto longissimae-Heliosciadietum crassipedis* Bagella, Rivieccio & Caria ass. nov. (Table 11)

Holotypus: rel. 10, tab. 11.

**Table 11:** *Isoëto longissimae-Helosciadetum crassipedis* Bagella et al. ass. nov.

Relevè Number	1	2	3	4	5	6	7	8	9	10 *	11	12
Altitude (m)	795	795	795	795	795	795	795	795	795	795	795	795
Surface (m^2^)	1	1	1	1	1	1	1	1	1	1	1	1
Coverage (%)	70	80	80	100	80	80	90	90	80	90	80	80
** Char. Association**												
*Isoët* *es longissima*	+	1	1	1	+	+	1	+	1	2	1	+
** Char. *Menthion cervinae* and *Apienion crassipedis***												
*Helosciadium crassipes*	2	2	2	3	2	2	2	2	2	1	+	1
*Ranunculus revelieri*	.	.	.	.	+	+	+	R	+	1	+	+
*Ranunculus cordiger* subsp. *diffusus*	.	+	.	.	.	.	.	.	.	.	.	.
** Char.** ***Isoë*** ***tetalia* and ** ***Isoë*** ***to-Nanojuncetea***												
*Agrostis pourretii*	1	1	1	2	.	.	.	1	1	2	1	+
*Mentha pulegium*	1	r	.	.	r	+	1	.	1	1	+	1
*Ranunculus sardous*	+	.	.	1	+	+	r	R	r	.	.	R
*Illecebrum verticillatum*	r	1	1	.	+	1	r	.	.	2	+	.
*Middendorfia borysthenica*	.	.	.	.	.	+	.	.	+	1	+	+
*Trifolium michelianum*	+	.	.	.	.	.	.	.	.	.	.	.
*Verojuncus pygmaeus*	.	.	.	.	.	.	.	.	.	.	.	R
** Other species**												
*Lotus pedunculatus*	1	+	1	2	r	+	1	+	1	3	1	1
*Bellis annua*	+	+	+	2	+	r	1	+	+	2	+	+
*Eleocharis palustris*	.	.	.	.	1	1	+	+	+	.	.	+
*Agathryon subulatum*	+	r	+	.	+	.	.	+	+	.	.	.
*Cynodon dactylon*	.	.	.	.	.	.	.	R	+	1	+	1
*Carex divisa*	.	.	.	.	.	.	.	+	1	.	.	.

**Localities and dates of relevés**: Rel. 1–4, Buddusò, Nuraghe Loelle, 13 March 2007; Rel. 5–7, Buddusò, Nuraghe Loelle, 20 April 2007; Rel. 8–12, Buddusò Nuraghe Loelle, 31 May 2007. The symbol (*) indicates the nomenclatural type as specified in the ICPN code.

Characteristic species: *Isoëtes longissima*.

Structure and ecology: In large ponds and ditches on granitic plateaus characterized by wooded grasslands at 700 m a.s.l., a vegetation dominated by *Helosciadium crassipes* occurs. In this stand, *Isoëtes longissima* is frequent together with other hygrophytes such as *Ranunculus revelieri* and *Illecebrum verticillatum*. This plant community, quite rare in Sardinia, is described as a new association, namely *Isoëto longissimae-Heliosciadetum crassipedis*, included in the *Apienion crassipedis*.

Geographical distribution: The association was observed near Buddusò in North Sardinia.

#### 2.3.11. *Middendorfio borysthenicae-Ranunculetum revelieri* Barbero 1965, Bull. Soc. Bot. Fr., 112: 279, nom. mut. nov. (Table 12)

**Table 12:** *Middendorfio borysthenicae-Ranunculetum revelieri* Barbero 1965, nom. mut.

Relevè Number	1	2	3	4	5	6	7	8 *	9	10
Altitude (m)	150	150	150	150	150	150	150	150	150	150
Surface (m^2^)	4	4	3	5	2	3	3	5	5	4
Coverage (%)	100	100	100	100	100	100	100	100	100	100
**Char. Association and *Apienion crassipedis***										
*Ranunculus revelieri*	2	2	1	2	1	3	4	3	4	4
*Isolepis setacea*	.	+	+	.	1	+	+	2	1	2
*Middendorfia borysthenica*	1	.	1	+	1	.	1	+	.	+
** Char. Subsssociation**										
*Illecebrum verticillatum*	1	+	1	2	1	1	2	3	2	2
** Char. *Menthion cervinae***										
*Callitriche truncata*	5	4	5	5	4	4	4	2	3	3
** Char.** ***Isoë*** ***tetalia* and ** ***Isoë*** ***to-Nanojuncetea***										
*Mentha pulegium*	1	2	1	1	2	2	1	2	1	1
*Isoët* *es tiguliana*	2	2	1	2	2	2	1	1	1	1
*Polypogon subspathaceus*	1	+	1	1	3	1	3	4	3	3
*Juncinella capitata*	+	+	.	+	.	.	+	.	.	.
*Peplis portula*	.	.	.	.	.	1	2	.	.	.
*Agathryon bufonium*	.	.	.	.	.	.	.	+	.	.
** Other species**										
*Potentilla reptans*	1	+	1	.	.	.	.	.	.	.

**Localities and dates of relevés**: Rel. 1–7, San Pietro Island 30 April 1995; Rel. 8–10, San Pietro Island 12 May 1994. The symbol (*) indicates the nomenclatural type as specified in the ICPN code.

Syn.: Association à *Peplis erecta* et *Ranunculus revelieri* Barbero 1965, Bull. Soc. Bot. Fr. 112: 279.

Lectotypus: rel. 2, tab. A [62], hoc loco.

Characteristic species: *Middendorfia borysthenica* (=*Peplis erecta*, *Lythrum borysthenicum)*, *Ranunculus revelieri*, *Isolepis setacea* (=*Scirpus setaceus*).

Structure and ecology: In the small ponds flooded during the winter–spring period, localized on volcanic substrates covered by a thin muddy layer, a vegetation characterized by the dominance of *Ranunculus revelieri* was observed. Usually, this species grows together with other hygrophytes, such as *Illecebrum verticillatum*, *Polypogon subspathaceus*, *Mentha pulegium*, *Isoëtes tiguliana*, etc. For the occurrence of *Middendorfia borysthenica* and *Isolepis setacea*, this plant community can be referred to as the *Peplido erectae-Ranunculetum revelieri* association described from the Maures massif in southern France by [62]. According to the art. 45 of the Code, the name of this syntaxon must be changed in *Middendorfio borysthenicae-Ranunculetum revelieri* nom. mut. nov. since in the most recent floras [63,64,65] the correct name for *Peplis portula* is *Middendorfia borysthenica*. In Sardinia, this association shows quite similar ecological requirements, being linked to markedly acidic substrata and a floristic set that one surveyed in France. But it differs for the constant occurrence of *Illecebrum verticillatum*, which is proposed as a differential species of a new subassociation, namely *illecebretosum verticillati* subass. nov. (holotypus rel. 8, tab. 11) to be considered a Sardinian geographic vicariant.

Nevertheless, it also hosts *Callitriche truncata*, which often has high coverage values, especially in the central part of these depressions.

Geographical distribution: The association is localized at San Pietro Island, in South-West Sardinia, at an elevation of about 150 m a.s.l.

#### 2.3.12. *Isoëto tigulianae-Callitrichetum brutiae* Bagella, Caria, Farris & Filigheddu 2009, Fitosociologia 46 (1): 15 (Table 13)

Holotypus: rel. 1, tab. 5 [23].

**Table 13:** *Isoëto tigulianae-Callitrichetum brutiae* Bagella et al., 2009.

Relevè Number	1	2	3	4	5	6	7
Altitude (m)	200	200	200	200	200	200	200
Surface (m^2^)	4	1	1	2	2	2	4
Coverage (%)	90	100	100	100	100	100	100
** Char. Association**							
*Callitriche brutia*	4	5	5	4	5	4	5
** Char. Subassociation**							
*Myriophyllum verticillatum*	.	.	.	3	3	3	2
** Char. *Menthion cervinae* and *Apienion crassipedis***							
*Helosciadium crassipes*	.	+		.	+	+	+
*Ranunculus baudotii*	+	.	.	+	.	2	1
** Char.** ***Isoë*** ***tetalia* and ** ***Isoë*** ***to-Nanojuncetea***							
*Isoët* *es tiguliana*	3	1	1	1	.	2	1
** Other species**							
*Glyceria spicata*	+	.	.	+	+	+	+

**Localities and dates of relevés**: Rel. 1–7, Olmedo (NW Sardinia), Bagella et al. (2009), Tab. 5.

Characteristic species: *Callitriche brutia*, *Isoëtes tiguliana*.

Structure and ecology: The association was surveyed in large temporary ponds on ignimbrite substrata with hydromorphic clay soils, submerged by quite deep freshwaters. It occurs at an elevation of 200 m, showing its optimum in early spring, within the thermoMediterranean bioclimatic belt. This vegetation is floristically very poor and results are dominated by *Callitriche brutia*, which shows high coverage values. It usually grows together with *Isoëtes tiguliana* and *Helosciadium crassipes*. It is an association belonging to *Apienion crassipedis*, within which two subassociations can be recognized as emphasized by [23]: subass. *Isoëtetosum tigulianae* (rel. 1–3) localized in the stands distant from the depression edge and subass *Myriophylletosum verticillati* (rel. 4–7) in the central part of the pond with deeper waters. For the dominance of *Callitriche brutia,* this association shows some relation with *Myriophyllo alterniflori-Callitrichetum brutiae* Cirujano, Pascual & Valayos 1986 described from the Iberian Peninsula, and with *Ranunculo lateriflori-Callitrichetum brutiae* Brullo & Minissale 1998 from Sicily.

Geographical distribution: The association was observed in the tableland near Olmedo in North-West Sardinia.

#### 2.3.13. *Loto conimbricensis-Ranunculetum revelieri* Brullo, Bacch., Giusso & Miniss. ass nov. (Table 14)

Holotypus: rel. 6, tab. 14.

**Table 14:** *Loto conimbricensis-Ranunculetum revelieri* Brullo et al. ass. nov.

Relevè Number	1	2	3	4	5	6 *
Altitude (m)	450	450	470	480	450	500
Surface (m^2^)	3	3	2	4	4	3
Coverage (%)	100	70	80	100	70	100
** Char. Association**						
*Lotus conimbricensis*	2	1	2	3	2	2
** Char. *Menthion cervinae* and *Apienion crassipedis***						
*Ranunculus revelieri*	2	2	3	4	2	4
*Callitriche brutia*	2	2	2	1	1	+
*Ranunculus saniculifolius*	1	1	+	2	1	+
** Char.** ***Isoë*** ***tetalia* and ** ***Isoë*** ***to-Nanojuncetea***						
*Agathryon bufonium*	1	+	+	2	1	+
*Lotus parviflorus*	2	+	1	2	.	2
*Mentha pulegium*	2	1	.	2	+	.
*Isoët* *es tiguliana*	3	3	2	1	.	.
*Juncinella capitata*	+	.	+	.	+	+
*Trifolium dubium*	1	.	+	+	+	.
*Polypogon subspathaceus*	.	.	1	1	.	1
*Damasonium bourgaei*	.	.	+	.	2	1
*Ranunculus sardous*	1	+	.	.	.	+
** Other species**						
*Glyceria spicata*	2	1	2	1	2	2
*Galium divaricatum*	.	.	1	+	.	.
*Geranium dissectum*	.	.	.	.	1	.
*Alopecurus bulbosus*	.	.	.	.	.	2

**Localities and dates of relevés**: Rel. 1–6, 2 May 1995, Road for Ardara, near Nuraghe Coloru. The symbol (*) indicates the nomenclatural type as specified in the ICPN code.

Characteristic species: *Lotus coninbricensis*, *Ranunculus revelieri*.

Structure and ecology: In the hilly stands, at an elevation of 450–500 m, limited to the ponds occurring on volcanic rocks with silty-clay soil, submerged in the winter–spring period, a peculiar amphibious vegetation physiognomically characterized by the dominance of *Ranunculus revelieri*, *Lotus conimbricensis,* and *Callitriche brutia* is localized. This plant community, proposed as *Loto conimbricensis-Ranunculetum revelieri*, is closely related to *Isoëto tigulianae-Callitrichetum brutiae*, from which it mainly differs in the occurrence of *Lotus conimbricensis*, usually showing high cover values. Moreover, several hygrophytes of the *Isoëto-Nanojuncetea* class are very frequent, such as *Agathryon bufonium*, *Lotus parviflorus*, *Mentha pulegium*, *Isoëtes tiguliana*, *Juncinella capitata*, *Trifolium dubium*, *Ranunculus saniculifolius*, etc.

Geographical distribution: The association is localized in North-West Sardinia in the Logudoro subregion, province of Sassari.

#### 2.3.14. *Exaculo pusilli-Lythretum portulae* Biondi & Bagella 2005, Fitosociologia 42 (2): 19 (Table 15)

Holotypus: rel. 1, tab. 17 [21].

**Table 15:** *Exaculo pusilli-Lythretum portulae* Biondi & Bagella 2005.

Relevè Number	1	2	3	4	5	6	7
Altitude (m)	-	-	-	-	-	-	-
Surface (m^2^)	5	4	4	3	3	4	3
Coverage (%)	70	60	70	70	70	40	100
** Char. Association and *Menthion cervinae***							
*Peplis portula*	2	2	3	2	2	3	3
*Exaculum pusillum*	2	2	1	1	1	1	.
** Char. Subassociation**							
*Baldellia ranunculoides*	+	.	.	2	2	1	3
*Paspalum distichum*	.	.	.	.	+	1	+
** Char.** ***Isoë*** ***tetalia* and ** ***Isoë*** ***to-Nanojuncetea***							
*Middendorfia borysthenica*	+	+	.	+	.	.	.
*Kickxia cirrhosa*	+	1	+	.	.	.	.
*Verojuncus pygmaeus*	2	+	+	2	+	+	.
** Other species**							
*Corrigiola telephifolia*	.	+	+	.	1	+	.

**Localities and dates of relevés**: Rel. 1–7, La Maddalena Archipelago, Biondi & Bagella (2005), Tab. 17.

Characteristic species: *Peplis portula*, *Exaculum pusillum*.

Structure and ecology: The association grows along the edges of periodically flooded artificial basins characterized by sandy siliceous soils of granitic origin. Floristically, it is a very poor plant community, showing its vegetative optimum from late spring to early summer. Although it was initially attributed to the *Cicendio-Solenopsion laurentiae* by [21], it must be more properly referred, due to its ecological requirements and the presence of *Peplis portula*, to the *Menthion cervinae* alliance represented in Sardinia by the *Apienion crassipedis*, as confirmed also by the cluster analysis. Within this association, two subassociations were recognized by [21]: *Lythretosum portulae* (rel. 1–3), localized in the marginal surfaces of these wetlands, and *Baldellietosum ranunculoides* (rel. 4–7), limited to the inner stands with more persistent waters.

Geographical distribution: Maddalena and Caprera Islands (North-East Sardinia).

#### 2.3.15. *Apio crassipedis-Isoëtetum tigulianae* Biondi & Bagella 2005, Fitosociologia 42 (2) Suppl.: 18 (Table 16, Table 17 and Table 18)

Holotypus: rel. 2, tab. 16 [21].

**Table 16:** *Apio crassipedis-Isoëtetum tigulianae* Biondi & Bagella 2005.

Relevè Number	1	2	3	4	5	6	7	8	9	10	11	12	13	14	15	16	17	18	19	20	21	22	23	24	25	26
Altitude (m)	-	-	-	-	-	-	-	-	-	-	-	-	-	-	-	-	-	-	-	-	-	-	-	-	-	-
Surface (m^2^)	2	2	2	2	2	2	4	4	1	2	2	2	2	4	4	4	4	4	1	4	2	6	8	3	4	3
Coverage (%)	100	90	100	100	100	100	100	90	90	100	100	100	100	100	100	100	100	100	100	100	100	80	80	100	100	90
** Char. Association**																										
*Isoët* *es tiguliana*	4	1	4	4	3	4	4	3	4	3	5	4	5	4	5	3	4	3	2	4	4	1	3	4	4	5
** Char. *Menthion cervinae* and *Apienion crassipedis***																										
*Helosciadium crassipes*	+	+	+	+	2	+	+	+	1	2	3	1	1	2	2	2	4	5	5	2	3	4	1	1	.	+
*Ranunculus ophioglossifolius*	1	2	1	+	3	3	3	3	2	3	2	3	1	2	2	2	1	+	+	+	1	.	.	.	.	.
*Ranunculus cordiger* ssp. *diffusus*	+	.	.	2	1	2	.	.	.	+	1	1	1	1	2	2	+	+	.	1	1	.	.	.	.	.
** Char. *Isoëtetalia* and *Isoëto-Nanojuncetea***																										
*Verojuncus pygmaeus*	+	.	.	+	+	r	.	.	.	1	.	.	+	1	+	.	.	.	.	1	1	.	.	2	1	2
*Myosotis sicula*	.	.	.	1	+	1	.	.	.	1	.	1	1	1	1	1	r	r	.	+	+	.	.	.	.	.
*Illecebrum verticillatum*	.	.	.	.	.	.	.	.	.	.	+	.	.	.	r	.	.	r	.	+	.	3	2	2	2	+
*Peplis portula*	.	.	.	.	.	.	.	.	.	.	.	.	.	.	.	.	.	.	.	.	.	.	1	1	1	.
*Isolepis cernua*	.	.	.	.	.	.	.	.	.	.	.	+	+	+	.	.	.	.	.	+	.	.	.	.	.	.
*Isoët* *es histrix*	.	.	+	.	.	.	.	.	1	.	.	.	+	+	.	.	.	.	.	.	.	.	.	.	.	.
*Lythrum hyssopifolia*	.	.	.	.	.	.	.	.	.	+	.	.	.	.	.	.	.	.	.	r	.	.	.	.	.	.
*Ophioglossum lusitanicum*	.	.	.	.	.	.	.	.	.	.	.	.	.	.	.	.	.	.	.	+	+	.	.	.	.	.
*Trifolium michelianum*	.	.	.	.	.	.	+	.	.	.	+	.	.	.	.	.	.	.	.	.	.	.	.	.	.	.
*Agathryon bufonium*	.	.	.	.	.	.	.	.	.	.	.	.	.	r	.	.	.	.	.	+	.	.	.	.	.	.
*Eudianthe laeta*	.	.	.	.	.	.	+	.	.	.	.	.	.	.	.	.	.	.	.	.	.	.	.	.	.	.
*Juncinella capitata*	.	.	.	.	.	.	.	.	.	.	.	.	+	.	.	.	.	.	.	.	.	.	.	.	.	.
*Eryngium pusillum*	.	.	.	.	.	r	.	.	.	.	.	.	.	.	.	.	.	.	.	.	.	.	.	.	.	.
*Buillardia vaillantii*	.	.	.	.	r	.	.	.	.	.	.	.	.	.	.	.	.	.	.	.	.	.	.	.	.	.
** Other species**																										
*Lotus pedunculatus*	2	+	2	+	2	2	1	2	.	+	+	+	+	1	+	.	1	1	+	2	1	.	.	.	.	.
*Bellis annua*	1	.	+	2	+	+	.	+	.	.	.	.	1	1	1	+	.	.	.	1	+	.	.	.	.	.
*Trifolium subterraneum*	.	.	.	1	.	.	.	.	.	+	.	+	+	+	+	+	.	.	.	.	+	.	.	.	.	.
*Alopecurus bulbosus*	+	.	.	.	.	+	.	.	.	1	+	+	+	+	.	.	.	.	.	.	+	.	.	.	.	.
*Eleocharis palustris*	+	.	.	.	.	.	.	.	.	.	.	.	.	.	.	.	+	r	.	.	.	1	+	2	1	.
*Carex divisa*	+	.	.	.	.	.	+	.	.	.	.	+	.	+	+	+	.	.	.	.	+	.	.	.	.	.
*Glyceria spicata*	.	.	.	.	.	.	+	.	+	.	.	.	.	.	.	.	+	1	1	.	.	.	.	.	.	.
*Ranunculus baudotii*	.	4	3	.	.	.	.	.	+	.	.	.	.	.	.	.	.	+	+	.	.	.	.	.	.	.
*Anthemis arvensis*	.	.	.	.	1	+	+	+	.	.	.	+	.	.	.	.	.	.	.	.	.	.	.	.	.	.
*Callitriche stagnalis*	.	+	+	.	.	.	.	.	.	.	.	.	.	.	.	.	+	+	.	.	.	.	.	.	.	.
*Paspalum distichum*	.	.	.	.	.	.	.	.	.	.	.	.	.	.	.	.	.	.	.	.	.	3	+	.	.	+
*Montia hallii*	.	.	.	.	.	.	.	.	.	.	.	.	+	.	+	.	.	.	.	.	+	.	.	.	.	.
*Linum bienne*	.	.	.	.	.	.	.	.	.	.	.	.	.	.	.	.	.	.	.	r	1	.	.	.	.	.
*Romulea requienii*	.	.	.	r	.	.	.	.	.	.	.	.	.	.	.	.	.	.	.	+	.	.	.	.	.	.

**Localities and dates of relevés**: Rel. 1–21 Olmedo (NW Sardinia), Bagella et al. (2009), Tab. 6; Rel. 22–26, La Maddalena Island, Biondi e Bagella (2005), Tab. 16.

**Table 17:** *Apio crassipedis-Isoëtetum tigulianae* Biondi & Bagella 2005.

Relevè Number	27	28	29	30	31	32	33	34	35	36	37	38	39	40	41	42	43	44	45	46	47	48	49	50	51	52	53	54	55
Altitude (dam)	13	13	13	7	7	7	7	31	31	31	58	58	58	58	58	58	58	58	58	58	58	58	58	58	58	58	57	57	57
Surface (m^2^)	1	1	1	8	6	4	1	6	4	8	2	2	3	4	3	2	3	3	3	2	2	2	2	3	4	3	2	2	2
Coverage (%)	90	70	60	90	80	100	80	80	90	90	90	90	100	100	100	100	100	100	100	100	100	100	100	100	100	90	100	100	100
** Char. Association**																													
*Isoëtes tiguliana*	2		2	4	3	2	3	3	1	3	2	+	1	1	+	+	1	1	+	1	1	1	1	1	2	1	+	1	1
** Char. *Menthion cervinae* and *Apienion crassipedis***																													
*Helosciadium crassipes*	4	3	3	.	.	.	.	.	.	.	2	3	3	2	2	2	1	2	1	1	2	2	1	3	4	3	2	2	2
*Ranunculus cordiger diffusus*	.	.	.	r	.	.	.	.	.	.	1	1	2	1	+	1	+	2	2	+	+	.	1	+	+	1	1	+	.
*Ranunculus revelieri*	.	.	.	.	.	.	.	.	.	.	+	2	1	2	+	3	4	3	3	3	2	1	+	1	1	2	1	2	2
*Ranunculus ophioglossifolius*	1	+	.	.	.	.	.	.	.	.	.	1	.	+	+	1	.	1	+	1	.	+	.	.	.	.	1	2	1
** Char. *Isoëtetalia* and *Isoëto-Nanojuncetea***																													
*Illecebrum verticillatum*	+	.	+	.	.	.	.	.	.	.	2	2	3	3	3	1	1	1	4	4	3	3	4	1	1	+	3	3	4
*Chamaemelum fuscatum*	.	.	.	+	+	1	+	2	r	1	2	1	1	1	2	2	.	+	1	+	1	1	2	.	.	.	.	.	.
*Antinoria insularis*	.	.	.	.	.	.	.	.	.	.	1	1	+	1	2	2	2	1	+	1	1	1	2	+	1	.	2	2	2
*Verojuncus tingitanus*	.	.	.	.	.	.	.	.	.	.	1	1	2	2	2	1	2	2	+	1	1	2	1	1	+	1	.	.	1
*Eryngium pusillum*	.	.	.	.	.	.	.	.	.	.	3	3	2	2	2	1	1	.	1	1	.	1	+	2	2	1	1	1	1
*Mentha pulegium*	.	.	.	.	.	.	.	.	.	.	+	1	1	2	1	+	.	1	1	.	+	1	2	2	1	2	3	2	2
*Peplis portula*	.	.	.	.	.	.	.	.	.	.	+	1	+	+	1	1	.	.	2	2	1	2	2	2	1	2	2	2	1
*Eudianthe laeta*	.	.	.	.	.	+	.	.	.	r	+	1	+	+	.	1	1	+	1	.	1	+	1	.	.	+	.	+	1
*Polypogon subspathaceus*	.	.	.	.	.	.	.	.	.	.	+	1	2	2	1	+	1	1	+	1	1	+	.	+	.	.	+	1	1
*Agathryon tenageia*	.	.	.	.	.	.	.	.	.	.	+	.	1	1	+	2	1	1	.	+	1	1	2	1	+	.	+	.	+
*Euphorbia falcata*	.	.	.	.	.	.	.	.	.	.	+	1	1	+	.	.	.	.	3	3	3	3	2	2	2	3	3	2	1
*Lotus hispidus*	.	.	.	.	.	.	.	r	r	.	1	+	1	1	1	.	+	1	.	.	+	+	.	.	.	.	+	1	1
*Agathryon bufonium*	.	.	.	.	.	+	+	+	.	.	+	+	1	+	+	.	.	.	.	.	.	.	.	.	.	.	1	2	1
*Poa infirma*	.	.	.	.	.	.	.	+	.	.	2	1	+	1	1	2	3	2	.	.	.	.	.	.	.	.	1	+	1
*Trifolium micranthum*	.	.	.	.	.	.	.	.	.	.	+	+	+	.	+	.	.	.	.	.	.	.	.	1	+	1	.	.	.
*Lythrum hyssopifolia*	.	.	.	+	+	1	+	1	+	+	.	.	.	.	.	.	.	.	.	.	.	.	.	.	.	.	.	.	.
*Myosotis sicula*	.	.	.	.	.	.	.	.	.	.	.	.	.	.	.	2	2	1	.	.	.	.	.	2	2	1	.	.	.
*Middendorfia borysthenica*	.	.	.	+	+	2	2	.	1	.	.	.	.	.	.	.	.	.	.	.	.	.	.	.	.	.	.	.	.
*Isolepis cernua*	.	.	.	.	.	.	.	.	.	.	.	.	.	.	.	.	.	.	.	.	.	.	.	+	1	+	.	.	.
*Sporobolus schoenoides*	.	.	.	.	.	.	.	.	.	.	1	.	.	.	1	.	.	.	.	.	.	.	.	.	.	.	.	.	.
*Ranunculus sardous*	.	.	.	.	+	+	.	.	.	.	.	.	.	.	.	.	.	.	.	.	.	.	.	.	.	.	.	.	.
*Agrostis pourretii*	.	.	3	.	.	.	.	.	.	.	.	.	.	.	.	.	.	.	.	.	.	.	.	.	.	.	.	.	.
*Juncinella capitata*	.	.	.	.	.	.	.	.	.	.	.	.	.	.	.	.	.	.	.	.	.	.	.	.	.	.	.	.	+
*Verojuncus pygmaeus*	.	.	.	.	.	.	.	+	.	.	.	.	.	.	.	.	.	.	.	.	.	.	.	.	.	.	.	.	.
** Other species**																													
*Bellis annua*	.	.	.	.	.	.	r	+	.	+	2	1	1	2	2	1	2	2	+	+	.	+	1	.	.	.	.	.	.
*Eleocharis palustris*	.	.	1	1	+	.	+	.	.	.	1	2	1	1	1	3	2	2	.	.	.	.	.	.	.	.	.	.	.
*Baldellia ranunculoides*	2	1	.	.	.	.	.	.	.	.	.	.	.	.	.	1	.	.	2	2	3	3	2	2	2	2	2	1	2
*Glyceria spicata*	.	.	1	3	1	3	2	1	4	3	.	.	.	.	.	.	.	.	.	.	.	.	.	.	.	.	.	.	.
*Alopecurus bulbosus*	2	2	.	.	.	.	.	.	.	.	1	2	2	2	1	.	.	+	.	.	.	.	.	2	1	1	.	.	.
*Trifolium resupinatum*	.	.	.	.	.	.	.	.	.	.	+	1	+	1	1	.	.	.	.	.	.	.	.	2	2	2	.	.	.
*Callitriche stagnalis*	.	.	.	4	3	1	2	2	2	4	.	.	.	.	.	.	.	.	.	.	.	.	.	.	.	.	.	.	.
*Linum bienne*	.	.	.	+	.	+	.	.	.	+	.	.	.	.	.	.	.	.	.	.	.	.	.	.	.	.	.	.	.
*Plantago coronopus*																											1	+	1
*Oenanthe fistulosa*	.	+	1	.	.	.	.	.	.	.	.	.	.	.	.	.	.	.	.	.	.	.	.	.	.	.	.	.	.
*Polypogon viridis*	1	.	+	.	.	.	.	.	.	.	.	.	.	.	.	.	.	.	.	.	.	.	.	.	.	.	.	.	.
*Ranunculus baudotii*	.	.	.	1	+	.	.	.	.	.	.	.	.	.	.	.	.	.	.	.	.	.	.	.	.	.	.	.	.
*Agathyron subulatum*	.	.	.	.	.	.	.	1	.	.	.	.	.	.	.	.	.	.	.	.	.	.	.	.	.	.	.	.	.
*Phalaris coerulescens*	.	.	*+*	.	.	.	.	.	.	.	.	.	.	.	.	.	.	.	.	.	.	.	.	.	.	.	.	.	.

Localities and dates of relevés: Rel. 27–29, Oristano, Mogoro, Rivieccio et al. (2020), Tab. 2; Rel. 30–36, Alghero-Scala Picada, -Rivieccio et al. (2022c), Tab. 4; Rel. 37–49, Giara di Gesturi, Pauli Majore, 1 May 1995; Rel. 50–52, Giara di Gesturi, 1 May 1995; Rel. 53–55, Giara di Gesturi, Pauli Aromena, 1 May 1995.

**Table 18:** *Apio crassipedis-Isoetetum tigulianae* Biondi & Bagella 2005.

Relevè Number	56	57	58	59	60	61	62	63	64	65	66	67	68	69	70	71	72	73	74	75
Altitude (m)	570	570	570	570	570	580	580	580	580	580	580	580	580	580	580	580	580	580	580	580
Surface (m^2^)	3	4	3	5	4	5	5	5	5	4	4	3	4	4	5	5	5	5	4	4
Coverage (%)	100	100	90	80	90	100	100	100	100	100	90	90	100	100	100	100	100	100	100	90
** Char. Association**																				
*Isoët* *es tiguliana*	1	2	2	1	1	3	2	2	3	2	3	1	2	2	3	2	2	3	2	3
** Char. *Menthion cervinae* and *Apienion crassipedis***																				
*Helosciadium crassipes*	1	+	1	1	1	1	2	2	1	1	2	1	1	1	1	2	2	1	1	2
*Ranunculus cordiger* subsp. *diffusus*	3	3	2	3	3	+	+	+	1	1	+	+	1	+	+	+	+	1	1	+
*Ranunculus revelieri*	.	.	.	.	.	.	.	.	.	.	.	2	2	3						
*Antinoria insularis*	.	.	.	.	.	.	.	.	.	.	.	+	+	1						
** Char. *Isoëtetalia* and *Isoëto-Nanojuncetea***																				
*Mentha pulegium*	+	1	+	.	.	+	2	1	1	+	2	1	1	+	+	2	1	1	+	2
*Eryngium pusillum*	.	.	.	.	.	3	4	3	3	4	2	1	2	2	3	4	3	3	4	2
*Polypogon subspathaceus*	.	.	.	.	.	1	2	2	1	.	+	.	.	.	1	2	2	1	.	+
*Illecebrum verticillatum*	+	1		+	1	.	.	.	.	.	.	.	.	.						
*Lotus hispidus*	.	.	.	.	.	+	+		+	.	.	+		+	+	+	.	1	.	.
*Middendorfia borysthenica*	1	2	2	1	2	1	+	+		1	1	1	1	+	1	+	+	.	1	1
*Trifolium micranthum*	.	.	.	.	.	.	+	1	1	.	2	2	3	2	.	+	1	1	.	2
*Poa infirma*	.	.	.	.	.	.	+	+	+	.	.	.	.	.	.	+	+	+	.	.
*Chamaemelum fuscatum*	.	.	.	.	.	.	.	.	.	.	.	+	1	1	.	.	.	.	.	.
*Myosotis sicula*	.	.	.	.	.	.	.	.	.	.	.	1	1	2	.	.	.	.		
** Other species**																				
*Baldellia ranunculoides*	2	3	3	2	2	2	2	2	2	2	3	1	1	2	2	2	2	2	2	3
*Alopecurus bulbosus*	2	1	1	2	1	2	1	2	2	2	1	2	1	2	2	1	2	2	2	1
*Bellis annua*	.	.	.	.	.	1	1	+	1	+		2	2	2	1	1	+	1	+	.
*Eleocharis palustris*	.	.	.	.	.	2	2	3	2	1	2	3	3	2	2	2	3	2	1	2
*Glyceria spicata*	3	3	2	2	3	2	1	2	1	2	2	1	+	1	2	1	2	1	2	2
*Oenanthe lisae*	.	.	.	.	.	2	1	2	1	2	2	2	2	1	2	1	2	1	2	2
*Trifolium angustifolium*	+	+	.	+	.	+	1	.	+	+	.	.	.	.	+	1	.	+	+	.
*Trifolium bocconei*	.	.	.	.	.	.	+	.	+	.	.	.	.	.	.	+	.	+	.	.

**Localities and dates of relevés**: Rel. 56–61, Giara di Gesturi, Pauli Aromena, 1 May 1995; Rel. 62–66, Giara di Gesturi, Pauli Majore, 1 May 1995; Rel. 67–75, Giara di Gesturi, Pauli Caruso, 1 May 1995.

Characteristic species: *Isoëtes tiguliana.*

Structure and ecology: The association is localized in temporary pools submerged during the springtime period by rainwater a few centimeters deep. It occurs mainly on siliceous substrates with muddy soils and is characterized by a dense coverage of *Isoëtes tiguliana*, an endangered species (Rossi et al., 2020) treated as a differential of the *Apienion crassipedis* and *Menthion cervinae*. Among the species of this alliance and suballiance are also present *Ranunculus cordiger* subsp. *diffusus*, *R. revelieri*, *R. ophioglossifolius,* and *Antinoria insularis*, while the *Isoëto-Nanojuncetea* class is represented by *Mentha pulegium*, *Eryngium pusillum*, *Polypogon subspathaceus*, *Illecebrum verticillatum*, *Lotus hispidus*, *Middendorfia borysthenica*, *Trifolium micranthum*, *Verojuncus tingitanus*, *Peplis portula*, and *Poa infirma.*

Geographical distribution: According to literature [21,23,25,26] and other releves, this association occurs in several localities of the northern and central Sardinia.

#### 2.3.16. *Apio crassipedis-Elatinetum macropodae* Bagella, Caria, Farris & Filigheddu 2009, Fitosociologia 46 (1): 15 (Table 19)

Holotypus: rel. 2, tab. 4 [23]

**Table 19:** *Apio crassipedis-Elatinetum macropodae* Bagella et al., 2009.

Relevè Number	1	2	3	4	5	6	7	8
Altitude (m)	200	200	200	200	642	642	642	642
Surface (m^2^)	1	1	1	1	1	1	1	1
Coverage (%)	90	90	90	90	100	90	100	90
** Char. Association**								
*Elatine macropoda*	5	4	4	4	4	3	3	2
** Char. *Menthion cervinae* and *Apienion crassipedis***								
*Helosciadium crassipes*	+	1	+	+	2	+	2	1
*Ranunculus ophioglossifolius*	+	+	1	+	.	.		
*Antinoria insularis*	.	1	+	+	.	.	2	2
*Callitriche brutia*	.	.	.	.	2	1	.	.
*Ranunculus revelieri*	.	.	.	.	.	.	.	+
** Char.** ***Isoë*** ***tetalia durieui* and ** ***Isoë*** ***to-Nanojuncetea***								
*Isoët* *e* *s tiguliana*	2	3	2	2	2	2	1	2
*Verojuncus pygmaeus*	1	1	1	1	+	1	+	1
*Middendorfia borysthenica*	+	+	1	.	2	+	2	1
*Buillardia vaillantii*	+	+	.	+			+	+
*Pilularia minuta*	.	.	.	.	+	2	2	2
*Damasonium bourgaei*	.	.	.	.	2	1	1	+
*Pulicaria vulgaris*	.	.	.	.	1	1	+	1
*Polypogon subspathaceus*	.	.	.	.	.	.	.	1
*Agrostis pourretii*	.	.	.	.	.	.	.	+
** Other species**								
*Callitriche stagnalis*	.	.	.	.	2	2	.	.
*Ranunculus baudotii*	.	.	.	.	2	1	.	.
*Eleocharis palustris*	.	.	.	.	.	2	1	1
*Glyceria spicata*	.	.	.	.	+	2	1	+
*Carex divisa*	.	.	.	.	1	.	1	.

**Localities and dates of relevés**: Rel. 1–4 Olmedo (NW Sardinia), Bagella et al. (2009), Tab. 4; Rel. 5–8 Monte Minerva, 21 April 2007.

Characteristic species: *Elatine macropoda.*

Structure and ecology: The association was surveyed in North-West Sardinia on tablelands of volcanic rocks dating back to Oligo-Miocene, at altitudes between 200 and 640 m. It colonizes small temporary pools flooded during the winter–spring period by shallow waters (10–20 cm). Physiognomically, it is characterized by *Elatine macropoda*, a species with a Mediterranean distribution [66], usually showing high coverage values, which grows with *Helosciadium crassipes* and *Isoëtes tiguliana*. This association, described by [23], for its floristic set and ecology, falls within the *Apienion crassipedis* even if the species of higher rank are sporadic and poorly represented. Among the most frequent are *Ranunculus ophioglossifolius*, *Antinoria insularis*, *Verojuncus pygmaeus*, *Middendorfia borysthenica*, *Buillardia vaillantii*, *Pilularia minuta*, etc.

Geographical distribution: According to the literature and new records, the association appears localized on Monte Rosso, Monte Miale Ispina [23], and Monte Minerva.

#### 2.3.17. *Ranunculo revelieri-Antinorietum insularis* Brullo, Bacch., Giusso & Miniss. ass. nov. (Table 20)

Holotypus: rel. 13, tab. 20.

**Table 20:** *Ranunculo revelieri-Antinorietum insularis* ass. nov.

Relevè Number	1	2	3	4	5	6	7	8	9	10	11	12	13 *	14
Altitude (m)	1050	1050	1050	1050	1050	1050	1050	570	570	570	570	580	580	580
Surface (m^2^)	3	4	4	3	4	4	4	3	4	4	4	3	3	3
Coverage (%)	90	100	100	100	100	100	100	100	100	100	100	90	100	100
** Char. Association**														
*Antinoria insularis*	+	+	1	2	2	1	2	2	2	1	2	1	2	2
** Char. *Menthion cervinae* and *Apienion crassipedis***														
*Ranunculus revelieri*	2	2	3	1	1	2	2	1	1	2	2	2	3	2
*Ranunculus cordiger* subsp. *diffusus*	+	1	+	.	1	1	.	.	1	1	.	1	2	2
*Ranunculus ophioglossifolius*	.	.	.	2	2	3	1	2	2	3	1	.	1	+
*Helosciadium crassipes*	1	1	1	.	.	.	.	.	.	.	.	1	1	1
** Char.** ***Isoë*** ***tetalia durieui* and ** ***Isoë*** ***to-Nanojuncetea***														
*Eryngium pusillum*	1	2	2	2	2	1	2	2	2	1	2	1	+	+
*Mentha pulegium*	1	1	+	+	1	2	2	+	1	2	2	2	1	1
*Myosotis sicula*	1	1	2	2	2	.	1	2	2	.	1	2	1	2
*Illecebrum verticillatum*	.	.	.	1	1	1	+	1	1	1	+	2	2	2
*Middendorfia borysthenica*	1	1	+	+	.	1	1	+	.	1	1		.	.
*Lotus hispidus*	+	.	+	+	+	.	.	+	+			+	1	1
*Poa infirma*	.	.	.	.	1	1	+		1	1	+	2	2	2
*Trifolium micranthum*	2	3	2	.	.	+	+	.	.	+	+	.	.	.
*Isoët* *e* *s tiguliana*	1	2	2	1	+	.	.	1	+			.	.	.
*Chamaemelum fuscatum*	+	1	1	.	.	.	.	.	.	.	.	1	.	1
*Euphorbia falcata*	.	.	.	.	.	.	.	+	1	2	1	.	.	.
*Verojuncus tingitanus*	.	.	.	.	.	.	.	.	.	.	.	2	1	1
*Polypogon subspathaceus*	.	.	.	.	.	.	.	.	.	.	.	2	2	1
*Peplis portula*	.	.	.	.	.	.	.	.	.	.	.	+	1	
*Eudianthe laeta*	.	.	.	.	.	.	.	.	.	.	.		1	1
*Agathryon tenageia*	.	.	.	.	.	.	.	.	.	.	.	+	1	.
** Other species**														
*Bellis annua*	2	2	2	2	1	2	1	2	1	2	1	1	1	1
*Alopecurus bulbosus*	2	1	2	2	1	2	2	2	1	2	2	1	+	.
*Baldellia ranunculoides*	1	1	2	1	1	2	1	1	1	2	1	.	+	2
*Eleocharis palustris*	3	3	2	+	1	1	.	+	1	1		2	3	3
*Oenanthe lisae*	2	2	1	4	5	4	4	4	5	4	4	.	.	.
*Glyceria spicata*	1	+	1	+	1	1	1	+	1	1	1	.	.	.
*Vulpia* sp.	.	.	.	+	+	.	+	+	+	.	+	.	.	.
*Leontodon tuberosum*	.	.	.	+	+	.	+	+	+	.	+	.	.	.
*Anagallis arvensis*	.	.	.	.	2	2	2	.	2	2	2	.	.	.

**Localities and dates of relevés**: Rel. 1–7, Badde Salighes, Catena del Marghine, 3 June 2002; Rel. 8–11, Giara di Gesturi, Pauli Aromena, 1 May 1995; Rel. 12–14, Giara di Gesturi, Pauli Maiore, 1 May 1995. The symbol (*) indicates the nomenclatural type as specified in the ICPN code.

Characteristic species: *Antinoria insularis, Ranunculus revelieri.*

Structure and ecology: On the Pliocenic basaltic plateaus of Sardinia, temporary wetlands are frequent, often covering very large surfaces; a vegetation with a late winter–spring cycle is localized. This ephemeral plant community, occurring at an altitude of 550–1050 m, is characterized by *Antinoria insularis*, often the dominant species, which has a scattered distribution in some central and eastern Mediterranean territories. Usually, it grows together with *Ranunculus revelieri*, also showing high coverage values, and several other species of *Apienion crassipedis* and *Menthion cervinae*, such as *Ranunculus cordiger* subsp. *diffusus*, *Ranunculus ophioglossifolius*, *Helosciadium crassipes.* Moreover, the species of higher rank are also well represented, among which *Eryngium pusillum*, *Mentha pulegium*, *Myosotis sicula*, *Illecebrum verticillatum*, *Middendorfia borysthenica*, *Lotus hispidus*, *Poa infirma*, *Trifolium micranthum*, *Isoetes tiguliana*, etc. For its floristical and ecological peculiarity, this plant community is proposed as a new association, namely *Ranunculo revelieri-Antinorietum insularis.* It can be considered a geographical vicariant of the *Ranunculo lateriflori-Antinorietum insularis*, association, described from Sicily by [67], where it grows approximately at 1000 m of elevation on Pliocene basalts in a wetland at the top of the Hyblean plateau. It is similarly characterized by the dominance of *Antinoria insularis*, while *Ranunculus revelieri* is replaced by *R. lateriflorus* [15].

Geographical distribution: In Sardinia, the association was surveyed in the tablelands of Giara di Gesturi and Badde Salighes.

#### 2.3.18. *Apio crassipedis-Antinorietum insularis* Brullo, Bacch., Giusso & Miniss. ass.nov. (Table 21)

Holotypus: rel. 1, tab. 21.

**Table 21:** *Helosciadio crassipedis-Antinorietum insularis* Brullo et al. ass. nov.

Relevè Number	1 *	2	3	4	5
Altitude (m)	570	570	570	570	570
Surface (m^2^)	2	2	2	2	2
Coverage (%)	100	100	100	100	100
** Char. Association**					
*Antinoria insularis*	3	2	3	3	4
** Char. *Menthion cervinae* and *Apienion crassipedis***					
*Helosciadium crassipes*	1	2	+	1	1
*Ranunculus cordiger* subsp. *diffusus*	1	.	+	+	+
** Char. *Isoëtetalia* and *Isoëto-Nanojuncetea***					
*Illecebrum verticillatum*	3	3	2	3	2
*Mentha pulegium*	2	3	2	2	3
*Poa infirma*	2	1	2	2	1
*Chamaemelum fuscatum*	1	1	2	1	1
*Euphorbia falcata*	2	2	1	2	2
*Eryngium pusillum*	1	+	1	1	+
*Polypogon subspathaceus*	2	2	+	1	+
*Trifolium micranthum*	1	+	.	2	1
*Eudianthe laeta*	1	1	2	.	+
*Juncinella capitata*	+	1	1	.	1
*Verojuncus tingitanus*	1	2	+	.	.
*Peplis portula*	.	.	+	1	+
*Lotus hispidus*	+	+	.	+	.
*Agathryon tenageia*	.	+	+	.	.
** Other species**					
*Bellis annua*	2	2	+	1	.
*Trifolium resupinatum*	.	+	1	1	1
*Plantago coronopus*	.	+	1	1	2
*Trifolium scabrum*	+	+	.	1	+
*Trifolium squarrosum*	1	.	+	+	1
*Baldellia ranunculoides*	.	+	+	+	1
*Eleocharis palustris*	.	.	.	+	1

**Localities and dates of relevés**: Rel. 1–5, Giara di Gesturi, Pauli Bartoli, 1 May 1995. The symbol (*) indicates the nomenclatural type as specified in the ICPN code.

Characteristic species: *Antinoria insularis, Helosciadium crassipes.*

Structure and ecology: In stands characterized by a shorter flooding period, represented by shallow depressions (5–10 cm deep), the *Ranunculo revelieri-Antinorietum insularis* is usually replaced by a plant community, in which *Antinoria insularis* is always dominant, but with a different floristic set. Indeed, it grows together with *Helosciadium crassipes*, while some hygrophytes linked to habitats with deeper waters are missing, such as, *Ranunculus revelieri*, *R. ophioglossifolius*, *Myosotis sicula*, and *Middendorfia borysthenica*. Therefore, this vegetation is proposed as a new association, falling always within *Apienion crassipedis*, namely *Apio crassipedis-Antinorietum insularis*.

Geographical distribution: Based on current observation, the association occurs in the Giara di Gesturi (Central Sardinia).

#### 2.3.19. *Isoëto tigulianae-Ranunculo lateriflori* Brullo, Bacch., Giusso & Miniss. ass.nov. (Table 22)

Holotypus: rel. 2, tab. 22.

**Table 22:** *Isoëto tigulianae-Ranunculo lateriflori* Brullo et al. ass.nov.

Relevè Number	1	2 *	3	4	5	6	7	8	9	10
Altitude (m)	1050	1050	1050	1050	1050	1050	1050	1050	1050	1050
Surface (m^2^)	1	1	1	1	1	3	2	1	1	3
Coverage (%)	100	100	90	80	80	80	80	90	90	90
** Char. Association**										
*Ranunculus lateriflorus*	2	4	3	4	3	4	1	2	3	2
*Isoët* *es tiguliana*	2	1	1	+	+	2	3	2	2	2
** Char. *Menthion cervinae* and *Apienion crassipedis***										
*Helosciadium crassipes*	1	1	+	.	+	+	.	.	+	.
*Antinoria insularis*	.	.	.	.	.	1	1	+	1	1
*Callitriche* cfr. *platycarpa*	.	.	.	.	.	.	2	+	1	3
** Char. *Isoëtetalia* and *Isoëto-Nanojuncetea***										
*Agathryon bufonium*	2	2	3	2	2	1	1	+	1	2
*Trifolium micranthum*	3	2	2	1	1	1	+	+	1	1
*Mentha pulegium*	2	2	2	1	1	2	2	1	2	1
*Hordeum geniculatum*	3	3	2	2	2	2	1	+	1	1
*Illecebrum verticillatum*	2	2	2	2	2	2	+	+	+	1
*Verojuncus pygmaeus*	.	2	3	2	2	3	2	+	1	2
*Lotus hispidus*	1	2	2	1	2	2	+	.	.	+
*Lotus angustissimus*	+	.	1	+	+	+	.	.	+	+
*Agrostis pourretii*	.	.	.	+	2	+	1	+	+	2
*Eryngium pusillum*	.	.	.	.	3	2	3	2	2	2
*Juncinella capitata*	1	+	+	+	+	+	.	.	.	.
*Lythrum hyssopifolia*	.	.	+	+	+	1	.	.	2	+
*Romulea ramiflora*	+	+	.	.	+	+	.	.	.	+
*Radiola linoides*	2	+	.	.	1	+	.	.	.	.
*Cynosurus polybracteatus*	+	.	1	+	1	.	.	.	.	.
*Peplis portula*	.	.	.	.	.	.	3	2	2	3
*Poa infirma*	.	.	1	.	.	+	+	.	.	.
*Myosotis sicula*	.	.	.	.	.	.	.	+	2	1
** Other species**										
*Alopecurus geniculatus*	2	2	2	2	3	3	2	2	2	2
*Bellis annua*	2	2	1	1	2	3	+	.	2	+
*Oenanthe crocata*	+	+	.	+	1	1	.	+	+	.
*Bromus hordeaceus*	1	+	.	+	1	1	.	+	.	.
*Glyceria spicata*	.	.	.	.	.	.	2	3	2	2
*Trifolium subterraneum*	.	1	1	+	+	.	.	.	.	.
*Trifolium* sp.	.	.	1	+	+	.	.	.	.	.
*Sedum glandulosum*	+	.	1	.	.	.	.	.	.	.
*Eleocharis palustris*	.	.	.	.	.	.	.	.	2	2

**Localities and dates of relevés**: Rel. 1–10, Badde Salighes, Marghine Chain, 3 June 2002. The symbol (*) indicates the nomenclatural type as specified in the ICPN code.

Characteristic species: *Ranunculus lateriflorus*, *Isoëtes tiguliana*.

Structure and ecology: In the small rocky pools of Pliocenic basaltic plateaus at about 1000 m of altitude, a very peculiar vegetation occurs, which is characterized by the dominance of *Ranunculus lateriflorus*, a hygrophyte very rare in Sardinia. These habitats, flooded for short periods, during springtime are covered by a dense vegetation in which, apart from the abovementioned species, there are also *Isoëtes tiguliana*, *Agathyron bufonium*, *Trifolium micranthum*, *Mentha pulegium*, *Hordeum geniculatum*, *Illecebrum verticillatum*, *Verojuncus pygmaeus*, *Lotus hispidus*, *Lotus angustissimus*, *Eryngium pusillum*, etc. For the occurrence of *Helosciadium crassipes*, *Antinoria insularis*, and *Callitriche* cfr. *platycarpa*, this plant community must be included in *Apienion crassipedis* and treated as a new association named *Isoëto tigulianae-Ranunculo lateriflori.*

Geographical distribution: Based on current knowledge, this association is localized at Badde Salighes in Marghine Catena (North-West Sardinia).

*CICENDIO-SOLENOPSION LAURENTIAE* Brullo & Miniss. 1998, Itinera Geobot. 11: 275.

Syn.: *Cicendion* auct. Medit. non Br.-Bl. 1967, Vegetatio 14: 28.

Holotypus: *Laurentio-Anthocerotetum dichotomi* Br.-Bl. 1936, Bull. Soc. Et. Sci. Nat. Nimes 47: 9.

Characteristic species: *Anagallis parviflora*, *Cicendia filiformis*, *Kickxia cirrhosa*, *Lysimachia minima*, *Ophioglossum lusitanicum*, *Radiola linoides*, *Solenopsis laurentia* subsp. *laurentia*.

Structure and ecology: According to [12,15,52], this alliance groups early spring plant communities with acidophilus requirements, with soils remaining humid for long periods linked to the thermoMediterranean bioclimatic belt. They are localized on waterlogged soils of large hollows with waterproof surfaces, sometimes represented by wood clearing, with soils rich in a sandy component. Aspects of this vegetation can also be surveyed in the rocky ponds with sandy soils. This alliance is varied in the Atlantic and mountain territories by the *Cicendion* (Rivas Goday in Rivas Goday & Borja 1961) Br.-Bl. 1967 (=*Radiolion linoidis* Pietsch 1973), falling into *Nanocyperetalia* order, which shows a late spring-summer vegetative optimum and is linked to Temperate bioclimate.

Geographical distribution: The alliance is well represented in the western and central Mediterranean area.

#### 2.3.20. *Junco capitati-Isoëtetum histricis* Br.-Bl. 1936, Bull. Soc. Et. Sci. Nat. Nimes 47: 148 (Table 23)

Holotypus: rel. pg. 149 [1]

**Table 23:** *Junco capitati-Isoëtetum histricis* Br.-Bl. 1936.

Relevè Number	1	2	3	4	5	6	7	8	9	10	11	12	13	14
Altitude (m)	580	580	580	580	580	580	610	610	610	434	434	78	79	92
Surface (m^2^)	5	4	4	4	5	4	2	2	2	1	1	3	4	2
Coverage (%)	100	100	100	100	100	100	100	90	90	90	90	100	90	90
** Char. Association**														
*Isoët* *es histrix*	2	2	3	2	2	1	3	+	2	3	2	2	2	2
** Char. *Cicendio-Solenopsion laurentiae***														
*Solenopsis laurentia* subsp. *laurentia*	2	3	3	2	2	2	2	3	2	+	.	.	.	+
*Cicendia filiformis*	1	2	2	3	2	2	.	+	2	1	+	.	.	1
*Agathryon tenageia*	2	2	1	+	2	1	1	2	+	.	.	.	.	.
*Anagallis parviflora*	+	+	1	1	1	2	2	2	1	.	.	.	.	.
*Eudianthe laeta*	+	1	1	2	1	+	1	+	2	1	+	.	.	.
*Exaculum pusillum*	+	1	1	+	1	2	.	.	.	.	.	.	.	.
*Radiola linoides*	+	.	+	+	.	.	.	+	1	.	.	.	.	.
*Kickxia cirrhosa*	.	.	.	.	.	.	+	.	.	.	.	.	.	.
*Isoët* *es gymnocarpa*	.	.	.	.	.	.	.	.	.	.	.	2	2	2
** Char. *Isoëtetalia* and *Isoëto-Nanojuncetea***														
*Juncinella capitata*	+	1	1	+	+	+	3	2	2	.	.	+	.	+
*Molineriella minuta*	2	1	1	+	2	1	.	.	.	.	.	.	.	
*Mentha pulegium*	1	1	2	2	2	1	2	+	+	.	.	.	.	1
*Lythrum hyssopifolia*	3	3	2	2	3	2	2	1	2	+	+	1	1	1
*Polypogon subspathaceus*	1	1	2	2	2	+	2	1	+	.	.	.	.	.
*Lotus parviflorus*	2	2	1	2	1	1	+	+	2	.	.	.	.	.
*Romulea ramiflora*	+	1	1	1	.	1	+	+	+	.	.	.	.	.
*Lotus angustissimus*	+	1	2	2	2	+	.	.	.	.	.	.	.	.
*Trifolium micranthum*	2	2	+	2	2	+	.	.	.	.	.	.	.	.
*Middendorfia borysthenica*	+	+	1	1	1	+	.	.	.	.	.	.	+	.
*Centaurium maritimum*	.	+	+	.	+	.	1	1	1	+	.	.	.	.
*Agathryon bufonium*	.	.	+	1	.	.	2	3	1	1	+	1	+	+
*Euphorbia falcata*	+	1	1	.	1	+	.	.	.	.	.	.	.	.
*Gaudinia fragilis*	+	+	1	1	+	.	.	.	.	.	.	.	.	.
*Myosotis sicula*	+	.	+	+	1	.	.	.	.	.	.	.	.	.
*Hordeum geniculatum*	+	+	.	.	+	.	.	.	.	.	.	.	.	.
*Poa infirma*	.	+	+	+	.	.	.	.	.	.	.	.	.	.
*Briza minor*	.	.	.	.	.	.	+	+	+	.	r	.	.	.
*Isolepis cernua*	.	.	.	.	.	.	+	1	1	.	.	.	.	.
*Lotus conimbricensis*	.	.	.	.	.	.	.	+	.	.	.	.	.	.
*Agrostis pourretii*	.	.	.	.	.	.	.	.	.	2	1	.	.	.
*Verojuncus pygmaeus*	.	.	.	.	.	.	.	.	.	1	2	.	.	+
*Eryngium pusillum*	.	.	.	.	.	.	.	.	.	+	.	.	1	1
*Buillardia vailantii*	.	.	.	.	.	.	.	.	.	.	.	.	+	.
** Other species**														
*Bellis annua*	1	1	1	+	1	1	2	1	+	+	+	1	1	.
*Leontodon tuberosum*	1	1	1	+	.	1	.	.	.	.	.	.	.	.
*Euphorbia exigua*	.	+	+	.	.	+	.	2	+	.	.	.	.	.
*Linum bienne*	.	.	.	.	.	.	1	+	1	.	.	+	+	+
*Serapias lingua*	.	.	.	.	.	.	+	+	+	.	.	.	.	.
*Trifolium campestre*	.	.	.	.	.	.	+	+	1	.	.	.	.	.
*Bellardia viscosa*	.	.	.	.	.	.	1	2	+	.	+	.	.	.
*Carex flacca* subsp. *serrulata*	.	.	.	.	.	.	+	1	.	+	.	.	+	+
*Vulpia myuros*	.	.	.	.	.	.	1	+	.	.	.	.	.	.
*Logfia gallica*	.	.	.	.	.	.	+	+	.	.	.	.	.	.
*Paronychia echinulate*	.	.	.	.	.	.	2	+	.	.	.	.	.	.
*Sagina* sp.	.	.	.	.	.	.	1	+	.	.	.	.	.	.
*Selaginella denticulata*	.	.	.	.	.	.	.	+	+	.	.	.	.	.
*Aira elegans*	.	.	.	.	.	.	.	+	+	.	.	.	.	.
*Trifolium leucanthum*	.	.	.	.	.	.	+	+	.	.	.	.	.	.
*Aira cupaniana*	.	.	.	.	.	.	+	.	.	.	.	.	.	.
*Dittrichia viscosa*	.	.	.	.	.	.	.	.	2	.	.	.	.	.
*Galium murale*	.	.	.	.	.	.	+	.	.	.	.	.	.	.
*Sedum glandulosum*	.	.	.	.	.	.	+	.	.	.	.	.	.	.
*Anthoxanthum ovatum*	.	.	.	.	.	.	1	.	.	.	.	.	.	.
*Tuberaria guttata*	.	.	.	.	.	.	+	.	.	.	.	.	.	.
*Triticum vagans*	.	.	.	.	.	.	+	.	.	.	.	.	.	.
*Plantago coronopus*	.	.	.	.	.	.	+	.	.				+	
*Trifolium bocconei*	.	.	.	.	.	.	.	+	.	.	.	.	.	.
*Bellardia trixago*	.	.	.	.	.	.	.	+	.	.	.	.	.	.
*Trachynia distachya*	.	.	.	.	.	.	.	+	.	.	.	.	.	.
*Allium subhirsutum*	.	.	.	.	.	.	.	.	+	.	.	.	.	.
*Bromus hordeaceus*	.	.	.	.	.	.	.	.	.	1	+	.	.	.
*Chamaemelum fuscatum*	.	.	.	.	.	.	.	.	.	1	1	1		1
*Cynosurus polybracteatus*	.	.	.	.	.	.	.	.	.	1	+	.	.	.
*Hordeum geniculatum*	.	.	.	.	.	.	.	.	.	+	+	.	.	.
*Linum usitatissimum* subsp. *angustifolium*	.	.	.	.	.	.	.	.	.	+	+	.	.	.
*Lotus parviflorus*	.	.	.	.	.	.	.	.	.	+	+	.	.	.
*Lysimachia foemina*	.	.	.	.	.	.	.	.	.	+	+	+	.	+
*Trifolium subterraneum*	.	.	.	.	.	.	.	.	.	1	+	1	1	.
*Anacamptis longicornu*	.	.	.	.	.	.	.	.	.	r	.	.	.	.
*Asphodelus ramosus*	.	.	.	.	.	.	.	.	.	+	.	+	r	r
*Carex divisa*	.	.	.	.	.	.	.	.	.	.	+	.	.	.
*Filago germanica*	.	.	.	.	.	.	.	.	.	.	+	.	.	.
*Macrobriza maxima*	.	.	.	.	.	.	.	.	.	+	.	.	.	.
*Oenanthe pimpinelloides*	.	.	.	.	.	.	.	.	.	.	+	.	.	+
*Plantago lanceolata*	.	.	.	.	.	.	.	.	.	+	.	.	.	.
*Trifolium campestre*	.	.	.	.	.	.	.	.	.	.	+	.	.	.
*Moraea sisyrinchium*	.	.	.	.	.	.	.	.	.	.	.	r	r	r
*Ranunculus sardous*	.	.	.	.	.	.	.	.	.	.	.	.	1	r
*Lotus conimbricensis*	.	.	.	.	.	.	.	.	.	.	.	.	r	r
*Alopecurus bulbosus*	.	.	.	.	.	.	.	.	.	.	.	.	.	1
*Anthoxanthum aristatum*	.	.	.	.	.	.	.	.	.	.	.	.	.	1
*Centaurium maritimum*	.	.	.	.	.	.	.	.	.	.	.	.	.	r
*Poa annua*	.	.	.	.	.	.	.	.	.	.	.	1	.	.
*Trifolium micranthum*	.	.	.	.	.	.	.	.	.	.	.	+	.	.
*Romulea ramiflora*	.	.	.	.	.	.	.	.	.	.	.	r	.	.
*Lotus hispidus*	.	.	.	.	.	.	.	.	.	.	.	.	+	.
*Montia hallii*	.	.	.	.	.	.	.	.	.	.	.	.	+	.
*Medicago minima*	.	.	.	.	.	.	.	.	.	.	.	.	r	.

**Localities and dates of relevés**: Rel. 1–9, Giara di Gesturi, 17 June 1996; Rel. 10–11 Usellus, Rivieccio et al. (2022d), Tab. 3, rel. 1–2; Rel. 12–14, Capo Frasca (Arbus), Caria and Bagella (2019), Tab. 2, rel. 1–3.

Characteristic species: *Isoëtes histrix.*

Structure and ecology: The association occurs in wetlands subject to temporary spring flooding, on basaltic or sometimes arenaceous substrates, with soils rich in sandy components. It is characterized by *Isoëtes histrix* and *Juncinella capitata* (=*Juncus capitatus*), growing together with numerous other microphytes of the *Isoëto-Nanojuncetea* class. The species of *Cicendio-Solenopsion laurentiae* is well represented here such as *Solenopsis laurentia* subsp. *laurentia*, *Cicendia filiformis*, *Agathyron tenageia*, *Anagallis parviflora*, *Eudianthe laeta*, *Exaculum pusillum*, *Radiola linoides*, etc. The *Junco capitati-Isoëtetum histricis* was described by [1] for the temporary coastal marshes of Tunisia and Algeria. Previously, it was included by this author in the *Isoëtion durieui*, but for its floristic and ecological peculiarities, it should be more correctly attributed to the *Cicendio-Solenopsion laurentiae* as highlighted by [12,68,69]. This association was included by [70] within the *Isoëtion durieui*, identifying several subassociations distributed in some localities of the western Mediterranean, without reporting it from Sardinia. More recently, several releves carried out in some Sardinian localities by [71,72] were attributed to *Isoëtion durieui*, which, although quite poor floristically, can be referred to this association.

Geographical distribution: In Sardinia, this association is localized in some places of the central west part of the island (Giara di Gesturi, Capo Frasca, Usellus). According to the literature data [1,68,69,70], it is recorded from North Africa and the Iberian Peninsula too.

#### 2.3.21. *Solenopsio laurentiae-Lythretum tribracteati* Brullo, Bacch., Giusso & Miniss. ass. nov. (Table 24)

Holotypus: rel. 2, tab. 24.

**Table 24:** *Solenopsio laurentiae-Lythretum tribracteati* Brullo et al. ass. nova.

Relevè Number	1	2 *	3	4	5	6
Altitude (m)	580	580	580	580	580	580
Surface (m^2^)	5	4	4	4	5	4
Coverage (%)	100	100	100	100	100	100
** Char. Association**						
*Lythrum tribracteatum*	3	3	2	2	3	2
** Char. *Cicendio-Solenopsion laurentiae***						
*Solenopsis laurentia* subsp. *laurentia*	2	3	3	2	2	2
*Cicendia filiformis*	1	2	2	3	2	2
*Agathryon tenageia*	2	2	1	+	2	1
*Eudianthe laeta*	+	1	1	2	1	+
*Exaculum pusillum*	+	1	1	+	1	1
*Anagallis parviflora*	+	+	1	1	1	2
** Char. *Isoëtetalia* and *Isoëto-Nanojuncetea***						
*Molineriella minuta*	2	1	1	+	2	1
*Trifolium micranthum*	2	2	+	2	2	+
*Mentha pulegium*	1	1	2	2	2	1
*Middendorfia borysthenica*	+	+	1	1	1	+
*Polypogon subspathaceus*	1	1	2	2	2	+
*Lotus angustissimus*	+	1	2	2	2	+
*Lotus hispidus*	2	2	1	2	1	1
*Romulea ramiflora*	+	1	1	1	.	1
*Gaudinia fragilis*	+	+	1	1	+	.
*Myosotis sicula*	+	.	+	+	1	.
*Poa infirma*	.	+	+	+	.	.
*Hordeum geniculatum*	+	+	.	.	+	.
** Other species**						
*Trifolium* sp.	1	1	+	+	+	1
*Bellis annua*	1	1	1	+	1	1
*Leontodon tuberosum*	1	1	1	+	.	1
*Euphorbia exigua*	.	+	+	.	.	+

**Localities and dates of relevés**: Rel. 1–6. Giara di Gesturi, 17 June 1996. The symbol (*) indicates the nomenclatural type as specified in the ICPN code.

Characteristic species: *Lythrum tribracteatum.*

Structure and ecology: The wetland affected by a quite long flooding period is characterized by an ephemeral, plant community having a late spring-early summer optimum. It is linked to silty-clay soils submerged by eutrophic waters mainly due to wild grazing. This vegetation is characterized by *Lythrum tribracteatum* hygrophyte fairly rare in Sardinia showing a late life cycle, which grows together with several elements of the *Cicendio-Solenopsion laurentiae*, such as *Solenopsis laurentia* subsp. *laurentia*, *Cicendia filiformis*, *Agathyron tenageia*, *Eudianthe laeta*, *Exaculum pusillum*, *Anagallis parviflora.* Overall, this plant community is floristically very rich for the occurrence of several higher-rank species, among them *Molineriella minuta*, *Trifolium micranthum*, *Mentha pulegium*, *Middendorfia borysthenica*, *Polypogon subspathaceus*, *Lotus angustissimus*, *Lotus hispidus*, *Romulea ramiflora*, etc. For its peculiarities, it is proposed as a new association namely *Solenopsio laurentiae-Lythretum tribracteati*, which shows some relations with other plant communities dominated by *Lythrum tribracteatum*, as *Ranunculo trilobi-Lythretum tribracteati* described from Sicily by [15].

Geographical distribution: This association is very rare in Sardinia, where it was surveyed on the basaltic tableland of Giara di Gesturi during the summertime.

#### 2.3.22. *Archidio alternifolii-Isoëtetum durieui* Brullo, Bacch., Giusso & Miniss. ass. nov. (Table 25)

Holotypus: rel. 2, tab. 25.

**Table 25:** *Archidio alternifolii-Isoëtetum durieui* Brullo et al. ass.nov.

Relevè Number	1	2 *	3	4	5	6	7	8	9 *	10	11	12	13
Altitude (m)	150	150	150	150	150	150	150	150	150	150	150	150	150
Surface (m^2^)	2	2	3	2	2	3	4	2	2	2	2	3	2
Coverage (%)	90	90	100	100	80	100	100	80	100	100	100	90	90
** Char. Association**													
*Isoët* *es durieui*	3	2	1	3	2	3	1	2	2	1	2	1	+
*Archidium alternifolium*	2	1	1	2	1	1	1	1	1	2	1	1	2
** Char. *Cicendio-Solenopsion laurentiae***													
*Solenopsis laurentia* subsp. *laurentia*	+	+	1	+	+	1	.	+	.	+	+	+	1
*Anagallis parviflora*	+	+	.	1	+	+	.	+	+	1	1	.	+
*Illecebrum verticillatum*	.	1	+	2	1	+	.	.	4	4	3	3	3
*Cicendia filiformis*	+	+	.	+	.	+	+	.	.	.	.	.	.
** Char. *Isoëtetalia* and *Isoëto-Nanojuncetea***													
*Mentha pulegium*	3	2	3	3	2	3	2	2	2	2	3	2	3
*Lythrum hyssopifolia*	2	1	1	+	2	2	1	1	2	2	2	3	2
*Polypogon subspathaceus*	2	2	2	2	+	3	2	2	3	2	3	2	2
*Juncinella capitata*	1	+	+	1	2	1	+	1	.	+	.	1	1
*Middendorfia borysthenica*	1	2	2	1	1	2	2	2	1	1	+	.	.
*Damasonium bourgaei*	1	2	2	2	1	2	3	2	.	.	.	.	.
*Isolepis cernua*	.	.	.	.	.	.	.	.	+	+	+	2	1
*Agathryon bufonium*	.	.	.	.	.	.	.	.	+	+	1	1	.
*Lotus hispidus*	+	.	+	.	.	+	.	.	.	.	.	.	.
** Other species**													
*Bellis annua*	+	.	.	+	+	.	+	1	.	.	.	.	.

**Localities and dates of relevés**: Rel. 1–8, San Pietro Island, Paradiso, 30 April 1995; Rel. 9–13, San Pietro Island, Paradiso, 12 May 1994. The symbol (*) indicates the nomenclatural type as specified in the ICPN code.

Characteristic species: *Isoëtes durieui*, *Archidium alternifolium*.

Structure and ecology: In the small ponds submerged by rain waters up to late springtime, occurring on volcanic acid Miocenic substrata with soils rich in a sandy component mixed with silt, vegetation characterized by *Isoëtes durieui*, was surveyed. For its edaphic peculiarities, this plant community results rich in elements of the *Cicendio-Solenopsion laurentiae*, such as *Solenopsis laurentia* subsp. *laurentia*, *Anagallis parviflora*, *Illecebrum verticillatum* and *Cicendia filiformis.* Moreover, *Archidium alternifolium*, a small moss, is quite frequent on the bottom of these ponds, allowing us to differentiate a new association proposed as *Archidio alternifolii-Isoëtetum durieui* and included in the *Cicendio-Solenopsion laurentiae*. Within this association, two subassociations can be distinguished, represented by the *Damasonietosum bourgaei* subass. nov. linked to muddy and shallow soils, differentiated by the occurrence of *Damasonium bourgaei* (holotypus: rel. 2) and by the *Illecebretosum verticillati* subass. nov., localized on sandy and deeper soils, differentiated by *Illecebrum verticillatum* (holotypus: rel. 9). From the literature, another association differentiated by *Archidium alternifolium* is *Archidio phascoidis-Isoëtetum velatae*, which was described from Sicily by [12] and mentioned also by [15].

Geographical distribution: This association was surveyed on the San Pietro Island in South-West Sardinia.

#### 2.3.23. *Illecebro verticillati-Corrigioletum litoralis* Brullo, Bacch., Giusso & Miniss. ass. nov. (Table 26)

Holotypus: rel. 1, tab. 26.

**Table 26:** *Illecebro verticillati-Corrigioletum litoralis* Brullo et al. ass. nova.

Relevè Number	1 *	2	3	4	5	6	7	8 *	9	10	11	12	13 *	14	15	16	17
Altitude (m)	150	150	150	150	150	150	75	75	75	75	75	75	15	15	150	150	150
Surface (m^2^)	2	3	3	2	1	1	2	3	3	4	2	3	4	2	3	3	2
Coverage (%)	90	80	80	80	50	90	100	100	100	100	100	100	90	80	90	100	80
** Char. Association**																	
*Corrigiola litoralis*	3	2	2	2	1	2	1	2	1	1	1	1	2	2	2	1	1
** Char. Subassociation**																	
*Ranunculus revelieri*	.	.	.	.	.	.	1	2	2	2	1	2	.	.	.	.	.
*Buillardia vaillantii*	.	.	.	.	.	.	.	.	.	.	.	.	4	3	3	3	2
** Char. *Cicendio-Solenopsion laurentiae***																	
*Illecebrum verticillatum*	1	2	2	3	2	2	3	3	5	4	4	5	1	1	3	4	3
*Anagallis parviflora*	2	2	+	+	+	+	1	+	1	1	+	+	+	.	+	.	+
*Solenopsis laurentia* subsp. *laurentia*	1	+	+	.	+	+	1	1	1	1	+	1	1	2	.	+	.
*Cicendia filiformis*	1	+	.	+	+	.	+	1	+	+	2	1	.	.	+	+	.
*Radiola linoides*	.	.	.	.	.	.	.	+	.	+	1	.	.	.	.	.	.
** Char. *Isoëtetalia* and *Isoëto-Nanojuncetea***																	
*Mentha pulegium*	2	1	2	2	1	2	2	2	1	2	2	2	3	2	2	2	1
*Polypogon subspathaceus*	3	3	2	2	1	2	3	2	2	3	2	2	2	3	2	1	2
*Lythrum hyssopifolia*	2	1	1	2	1	1	1	+	+	1	+	1	2	1	.	1	1
*Agathryon bufonium*	.	+	1	1	1	.	+	+	1	+	.	+	.	.	+	1	1
*Peplis portula*	1	+	2	2	2	3	3	4	3	3	3	1	.	.	.	1	2
*Centaurium maritimum*	+	1	+	.	+	.	.	.	.	.	.	.	+	+	+	+	1
*Isolepis cernua*	.	.	.	.	.	.	.	2	+	1	1	2	.	.	.	.	.
*Isoët* *es tiguliana*	.	.	.	.	.	.	.	.	.	+	+	.	.	.	.	.	.
*Verojuncus pygmaeus*	.	.	.	.	.	.	.	.	.	.	.	.	.	.	.	2	+
*Pulicaria vulgaris*	.	+	.	.	.	.	.	.	.	.	.	.	.	.	.	.	.
** Other species**																	
*Plantago coronopus*	3	3	1	+	2	.	.	+	+	.	+	.	2	2	1	+	2
*Coleostephus myconis*	1	+	+	.	+	.	.	.	.	.	.	.	.	.	.	.	.

**Localities and dates of relevés**: Rel. 1–6, San Pietro Island, Paradiso, 30.4.1995; Rel. 7–12, San Pietro Island, Bivio per Spalmatore, 12 May 1994; Rel. 13–14, San Pietro Island, La Punta, 12 May 1994; Rel. 15–17, San Pietro Island, Paradiso, 12 May 1994. The symbol (*) indicates the nomenclatural type as specified in the ICPN code.

Characteristic species: *Corrigiola litoralis.*

Structure and ecology: In the rocky pools submerged for long periods by rainwaters, on substrata represented by Miocene volcanic rocks, localized in the coastal and inland stands at an altitude not exceeding 150 m a.s.l., a peculiar vegetation occurs. It is characterized by *Corrigiola litoralis*, usually growing together with *Illecebrum verticillatum*, *Peplis portula*, and *Polypogon subspathaceus* often showing high coverage value. It is linked to markedly incoherent soils resulting from the erosion of the volcanic rocks, which justifies the occurrence of *Cicendio-Solenopsion laurentiae* elements, such as *Anagallis parviflora*, *Solenopsis laurentia* subsp. *laurentia*, *Cicendia filiformis*, *Radiola linoides*, etc. It is proposed as a new association, namely *Illecebro verticillati-Corrigioletum litoralis*, within which three subassociations can be distinguished. They are: *typicum*, occurring on less deep soils; *Ranunculetosum revelieri* subass. nov., limitedly to the deeper soils and differentiated by *Ranunculus revelieri* (holotypus: rel. 8); *Bulliardetosum vaillantii* subass. nov. growing on shallower soils with *Bulliarda vaillantii* as differential species (holotypus: rel. 13). Previously, an association characterized by *Corrigiola litoralis*, named *Coronopo squamati-Corrigioletum litoralis* was described by [15] from Sicily, where it is always localized in submountain stands.

Geographical distribution: This association was observed on the San Pietro Island in South-West Sardinia.

#### 2.3.24. *Solenopsio laurentiae-Isolepidetum cernuae* Gehu, Kaabache & Gharzuoli 1994, Coll. Phytosoc. 22: 304 (Table 27)

Holotypus: rel. 11, tab. 9 [73].

**Table 27:** *Solenopsio laurentiae-Isolepidetum cernuae* Gèhu, Kaabache & Gharzuoli 1993 corr.

Relevè Number	1	2	3	4	5	6	7	8	9	10	11	12	13	14	15	16	17	18	19	20	21	22	23	24
Altitude (m)	10	10	10	10	10	10	10	10	10	10	10	10	10	10	10	10	-	-	-	-	-	-	-	-
Surface (m^2^)	3	2	2	1	1	1	1	2	1	2	3	3	2	1	2	2	0.5	1	0.5	1	0.5	0.5	0.8	2
Coverage (%)	90	100	100	100	100	100	100	100	100	100	100	100	100	100	100	100	60	80	100	60	80	50	50	80
** Char. Association**																								
*Isolepis cernua*	2	2	3	3	2	3	3	3	2	3	2	1	2	2	1	2	2	3	4	4	4	3	2	4
** Char. *Cicendio-Solenopsion laurentiae***																								
*Exaculum pusillum*		2	1	2	1	3	1	3	2	1	2	1	1	+	1	2	.	.	.	.	.	.	.	.
*Solenopsis laurentia*	2	+	2	2	2	1	2	3	1	+	+	1	.	.	.	2	.	.	.	.	.	+	2	1
*Radiola linoides*		.	+	+	1	.	.	.	+	.	+	+	.	+	1	2	.	.	.	+	+	+	.	.
*Anagallis parviflora*	1	.	.	+	+	.	+	.	+	+	.	.	1	+	+	+	.	.	.	.	.	.	.	.
*Kickxia cirrhosa*		.	+	+	+	.	1	+	.	+	+	+	.	.	+	.	.	.	.	.	.	.	.	.
*Cicendia filiformis*		.	+	+	+	.	.	+	.	.	1	+	+	+	+	.	.	.	.	.	.	.	.	.
*Eudianthe laeta*	1	.	.	.	+	+	+	.	+	+	.	.	.	1	.	.	.	.	.	+	.	.	+	.
*Agathryon tenageia*	.	.	.	.	1	.	.	+	1	.	1	.	+	.	.	1	.	.	.	.	.	.	.	.
*Lysimachia minima*	.	.	.	.	.	.	.	.	.	.	.	.	.	.	.	.	.	.	.	.	.	.	+	.
** Char. *Isoëtetalia***																								
*Lotus angustissimus*	+	2	2	1	1	+	+	1	2	+	2	3	2	1	2	1	.	.	.	.	.	.	.	.
*Centaurium maritimum*		.	.	+	.	.	.	.	.	+	+	.	.	.	.	.	.	.	.	.	.	.	+	+
*Briza minor*	2	.	.	.	.	.	.	.	.	.	.	.	.	+	.	.	.	.	.	.	.	.	1	1
*Isoët* *es histrix*	.	.	.	.	.	.	.	.	.	.	.	.	.	.	.	.	.	.	.	+	+	+	.	.
** Char. *Isoëto-Nanojuncetea***																								
*Agathryon bufonium*	1	2	3	+	3	+	2	1	1	2	2	2	3	2	1	2	1	2	+	1	1	2	1	2
*Juncinella capitata*	.	1	+	+	3	.	1	.	1	1	+	2	.	1	2	3	.	.	.	2	2	+	+	1
*Polypogon subspathaceus*	2	2	1	2	2	2	2	1	2	3	1	1	3	2	1	2	.	.	.	.	.	+	+	1
*Verojuncus pygmaeus*	1	3	3	3	1	3	2	3	4	3	3	1	4	3	2	2	.	.	.	.	.	.	.	.
*Lythrum hyssopifolia*	2	3	3	1	2	2	3	1	3	2	1	3	1	1	2	1	.	.	.	.	.	.	.	.
*Romulea requienii*	+	.	.	.	.	.	.	.	.	.	.	.	.	.	.	.	.	.	.	+	.	.	.	.
*Mentha pulegium*	3	.	.	.	.	.	.	.	.	.	.	.	.	.	.	.	.	.	.	+	.	.	.	.
*Trifolium micranthum*	1	.	.	.	.	.	.	.	.	.	.	.	.	.	.	.	.	.	.	.	.	.	.	.
*Peplis portula*	.	.	.	.	.	.	.	.	.	.	.	.	.	.	.	.	.	.	.	.	.	+	.	.
*Ranunculus ophioglossifolius*	.	.	.	.	.	.	.	.	.	.	.	.	.	.	.	.	.	.	+	.	.	.	.	.
*Gaudinia fragilis*	2	.	.	.	.	.	.	.	.	.	.	.	.	.	.	.	.	.	.	.	.	.	.	.
** Other species**																								
*Bellis annua*	.	.	.	.	.	.	.	.	.	.	.	.	.	.	.	.	.	.	.	1	1	+	.	.
*Anagallis arvensis*	.	.	.	.	.	.	.	.	.	.	.	.	.	.	.	.	.	.	.	1	+	1	.	.
*Anagallis foemina*	.	.	.	.	.	.	.	.	.	.	.	.	.	.	.	.	.	.	.	.	.	.	+	2
*Spergularia marina*	.	.	.	.	.	.	.	.	.	.	.	.	.	.	.	.	+	+	.	.	.	.	.	.
*Plantago coronopus*	+	.	.	.	.	.	.	.	.	.	.	.	.	.	.	.	.	.	.	.	.	.	.	.
*Coleostephus myconis*	1	.	.	.	.	.	.	.	.	.	.	.	.	.	.	.	.	.	.	.	.	.	.	.
*Portulaca oleracea*	.	.	.	.	.	.	.	.	.	.	.	.	.	.	.	.	.	.	+	.	.	.	+	.

**Localities and dates of relevés**: Rel. 1. Asinara Island, 1 June 2002; Rel. 2–16, Baia del Sole, Olbia, 21 June 1996; Rel. 17–24, Caprera Island, Biondi & Bagella (2005) Tab. 18.

Characteristic species: *Isolepis cernua*.

Structure and ecology: The wet pools characterized by sandy-gravelly soils occurring in coastal habitat, submerged during the late spring-summer period, host a vegetation characterized by small microphytes, in which *Isolepis cernua* is dominant. It is a plant community rich in elements of *Cicendio-Solenopsion laurentiae*, such as *Exaculum pusillum*, *Solenopsis laurentia* subsp. *laurentia*, *Radiola linoides*, *Anagallis parviflora*, *Kickxia cirrhosa*, *Cicendia filiformis*, *Eudianthe laeta*, etc. The species of the *Isoeto-Nanojuncetea* class are also well represented, among them *Agathyron bufonium*, *Juncinella capitata*, *Polypogon subspathaceus*, *Verojuncus pygmaeus*, *Lythrum hyssopifolia*, etc. For its floristical and ecological peculiarities, this vegetation can be referred to, as proposed by [21], as *Solenopsio laurentiae-Isolepidetum cernuae*, an association described by [73] from Algeria. It had already been proposed as a nomen invalid. (art. 5) by [74] based on a single relevé carried out in the same territory.

Geographical distribution: In Sardinia, based on literature [21] and new records, the association occurs in a scattered way in some localities in the northern part of the island.

#### 2.3.25. *Kickxio cirrhosae-Exaculetum pusilli* Brullo, Bacch., Giusso & Miniss. ass nov. (Table 28)

Holotypus: rel. 3, tab. 28.

**Table 28:** *Kickxio cirrhosae-Exaculetum pusilli* Brullo et al. ass nov.

Relevè Number	1	2	3 *	4
Altitude (m)	5	5	5	5
Surface (m^2^)	2	2	3	1
Coverage (%)	100	100	100	100
** Char. Association**				
*Exaculum pusillum*	2	2	3	2
*Kickxia cirrhosa*	2	3	2	2
** Char. *Cicendio-Solenopsion laurentiae***				
*Anagallis parviflora*	2	2	1	2
*Solenopsis laurentia* subsp. *laurentia*	3	2	2	2
*Agathryon tenageia*	2	1	2	1
*Cicendia filiformis*	1	2	1	1
*Radiola linoides*	1	1	+	2
*Eudianthe laeta*	+	1	1	+
*Lysimachia minima*	.	.	+	+
** Char. *Isoëtetalia* and *Isoëto-Nanojuncetea***				
*Verojuncus pygmaeus*	2	3	2	2
*Agathryon bufonium*	2	1	2	2
*Isolepis cernua*	2	2	1	2
*Lotus angustissimus*	2	2	2	2
*Lythrum hyssopifolia*	1	2	2	1
*Polypogon subspathaceus*	+	1	1	+
*Juncinella capitata*	1	+	+	.
*Briza minor*	+	+	.	.
*Centaurium maritimum*	.	.	+	+
** Other species**				
*Sesamoides interrupta*	1	1	1	+

**Localities and dates of relevés**: Rel. 1–4, Baia del Sole, Olbia, 21 June 1996. The symbol (*) indicates the nomenclatural type as specified in the ICPN code.

Characteristic species: *Exaculum pusillum*, *Kickxia cirrhosa*.

Structure and ecology: A floristically very peculiar vegetation was observed in a stand of sandy coast, limited to small surfaces flooded for long periods with soils rich in sandy-silty components. It is characterized by the dominance of *Exaculum pusillum* and *Kickxia cirrhosa*, growing together with several other taxa of *Cicendio-Solenopsion laurentiae*, such as *Anagallis parviflora*, *Solenopsis laurentia* subsp. *laurentia*, *Agathyron tenageia*, *Cicendia filiformis*, *Radiola linoides*, *Eudianthe laeta* and *Lysimachia minima.* Several other hygrophytes of *Isoeto-Nanojuncetea* are quite frequent, especially *Verojuncus pygmaeus*, *Agathyron bufonium*, *Isolepis cernua*, *Lotus angustissimus*, *Lythrum hyssopifolia*, *Polypogon subspathaceus*, *Juncinella capitata*, etc. This plant community is proposed as a new association, namely *Kickxio cirrhosae-Exaculetum pusilli*. For the occurrence of *Exaculum pusillum*, this association shows some similarity with *Exaculo pusilli-Lythretum portulae*, an association described for Sardinia by [21] from which it differs floristically and ecologically, being included in the *Menthion cervinae*.

Geographical distribution: This association is very rare surveyed in a single locality of North-East Sardinia.

#### 2.3.26. *Romuleo requieni-Bellidetum bellidioidis* Biondi & Bagella 2005, Fitosociologia 42 (2) Suppl. 1: 16 (Table 29)

Holotypus: rel. 6, tab. 11 [21].

**Table 29:** *Romuleo requieni-Bellidetum bellidioidis* Biondi & Bagella 2005.

Relevè Number	1	2	3	4	5	6	7	8	9	10	11	12	13	14	15	16	17	18	19	20	21	22
Altitude (m)	355	355	610	610	680	975	970	-	-	-	-	-	-	-	-	-	-	-	-	-	-	-
Surface (m^2^)	0,2	0,3	2	1	2	1	1	1	2	2	2	6	2	2	2	2	1	1	10	10	6	1
Coverage (%)	80	100	100	90	100	80	80	50	85	85	50	40	70	90	80	80	80	90	60	50	80	70
** Char. Association**																						
*Bellium bellidioides*	1	1	3	2	1	2	3	3	4	4	2	3	3	4	4	4	4	2	2	1	2	.
*Romulea requienii*	.	.	+	+	1	.	.	.	.	.	1	1	+	.	.	3	.	1	2	3	+	2
*Crocus minimus*	.	.	.	.	.	.	.	.	.	.	.	.	.	+	+	.	.	.	2	1	.	1
*Colchicum verlaqueae*	.	.	.	.	.	.	.	.	.	.	.	.	.	.	1	.	.	.	3	1	3	2
** Char. *Cicendio-Solenopsion laurentiae***																						
*Solenopsis laurentia*	1	4	2	2	1	.	.	.	.	.	.	.	1	2	4	.	1	+	.	.	.	.
*Radiola linoides*	4	1	.	+	1	+	1	+	.	+	.	.	.	.	.	.	.	.	.	.	.	.
*Cicendia filiformis*	.	.	.	.	+	+	.	.	.	.	.	.	+	+	+	.	1	+	.	.	.	.
*Anagallis parviflora*	.	.	1	1	1	2	1	.	.	.	.	.	.	.	.	.	.	.	.	.	.	.
*Lysimachia minima*	.	.	2	1	.	.	.	.	.	.	.	.	.	.	.	.	+	+	.	.	.	.
*Eudianthe laeta*	.	.	+	.	1	.	.	.	+	.	.	.	.	.	.	.	.	.	.	.	.	.
*Exaculum pusillum*	.	.	.	.	.	.	.	.	.	.	.	.	1	.	.	.	+	.	.	.	.	.
*Kickxia cirrhosa*	.	.	.	+	+	.	.	.	.	.	.	.	.	.	.	.	.	.	.	.	.	.
*Agathryon tenageia*	.	.	.	.	1	.	.	.	.	.	.	.	.	.	.	.	.	.	.	.	.	.
** Char. *Isoëtetalia* and *Isoëto-Nanojuncetea***																						
*Polypogon subspathaceus*	2	3	1	2	2	1	.	1	2	1	3	3	2	2	1	2	1	+	.	1	1	.
*Agathryon bufonium*	+	2	1	1	2	2	+	1	+	.	.	.	+	.	.	.	.	.	.	.	.	.
*Centaurium maritimum*	.	.	+	1	+	+	.	.	+	.	.	.	+	1	+	.	.	.	+	+	.	.
*Mentha pulegium*	.	.	+	+	1	.	.	1	+	+	.	.	1	.	.	.	3	1	.	.	.	.
*Lythrum hyssopifolia*	+	3	+	2	2	.	.	.	.	.	.	.	.	.	.	.	+	.	.	.	.	.
*Juncinella capitata*	.	.	.	.	2	+	+	+	+	.	.	.	1	.	.	.	.	.	.	.	.	.
*Lotus angustissimus*	2	1	.	.	.	2	2	.	.	.	.	.	.	.	.	.	+	.	.	.	.	.
*Briza minor*	.	.	1	+	+	+	.	.	.	.	.	.	.	.	.	.	.	.	.	.	.	.
*Isoët* *es durieui*	.	.	+	.	3	+	+	.	.	.	.	.	.	.	.	.	.	.	.	.	.	.
*Agrostis pourretii*	.	.	.	.	+	.	.	.	.	.	.	.	.	.	.	.	+	.	.	.	.	.
*Isoët* *es histrix*	.	.	.	.	1	.	.	.	.	1	.	.	.	.	.	.	.	.	.	.	.	.
*Lotus parviflorus*	.	.	.	+	1	.	.	.	.	.	.	.	.	.	.	.	.	.	.	.	.	.
*Cyperus fuscus*	2	.	.	.	.	.	.	.	.	.	.	.	.	.	.	.	.	.	.	.	.	.
*Verojuncus pygmaeus*	.	.	.	.	1	.	.	.	.	.	.	.	.	.	.	.	.	.	.	.	.	.
*Isolepis cernua*	.	.	.	.	.	.	.	.	.	+	.	.	.	.	.	.	.	.	.	.	.	.
*Romulea ramiflora*	.	.	.	.	.	1	+	.	.	.	.	.	.	.	.	.	.	.	.	.	.	.
** Other species**																						
*Plantago weldenii*	.	.	.	.	.	.	.	+	1	1	.	.	.	1	1	1	+	+	2	1	1	1
*Logfia gallica*	.	.	.	+	+	+	.	.	.	.	.	.	.	.	.	.	.	.	1	1	1	+
*Sagina maritima*	.	.	.	.	.	.	.	.	.	.	+	+	.	1	+	.	2	4	.	.	.	.
*Anagallis foemina*	.	.	.	.	.	.	.	+	+	1	.	.	1	.	.	+	.	.	.	.	.	.
*Polycarpon tetraphyllum*	.	.	.	.	.	.	.	.	2	1	.	.	1	+	2	.	.	.	.	.	.	.
*Trifolium campestre*	.	+	2	1	+	.	+	.	.	.	.	.	.	.	.	.	.	.	.	.	.	.
*Vulpia myuros*	.	2	+	+	+	.	.	.	.	.	.	.	.	.	.	.	.	.	.	.	.	.
*Linum bienne*	1	.	+	+	+	.	.	.	.	.	.	.	.	.	.	.	.	.	.	.	.	.
*Bellis annua*	.	.	+	+	1	2	1	.	.	.	.	.	.	.	.	.	.	.	.	.	.	.
*Serapias lingua*	.	.	+	+	+	.	.	.	.	.	.	.	.	.	.	.	.	.	.	.	.	.
*Bellardia viscosa*	.	.	+	1	+	.	.	.	.	.	.	.	.	.	.	.	.	.	.	.	.	.
*Euphorbia exigua*	.	.	1	1	1	.	.	.	.	.	.	.	.	.	.	.	.	.	.	.	.	.
*Trachynia distachya*	.	.	+	+	+	.	.	.	.	.	.	.	.	.	.	.	.	.	.	.	.	.
*Tuberaria guttata*	.	.	.	.	+	.	.	.	.	.	.	.	.	.	.	.	.	.	+	+	.	.
*Anthoxanthum aristatum*	.	.	.	.	.	.	.	.	1	+	.	.	.	.	.		.	+	.	.	.	.
*Rumex bucephalophorus*	.	.	.	.	.	.	.	.	.	.	.	.	.	.	.	.	.	.	+	+	1	.
*Catapodium rigidum*	.	.	.	.	.	.	.	.	.	.	.	.	.	.	.	.	.	.	1	1	+	.
*Anagallis arvensis*	.	.	.	.	.	.	.	.	.	.	.	.	.	.	.	.	.	.	+	+	1	.
*Plantago bellardii*	.	.	.	.	.	.	.	.	.	.	2	2	.	.	.	.	.	.	.	.	.	.
*Hypericum scruglii*	.	.	+	+	.	.	.	.	.	.	.	.	.	.	.	.	.	.	.	.	.	.
*Bellardia trixago*	.	.	+	+	.	.	.	.	.	.	.	.	.	.	.	.	.	.	.	.	.	.
*Hypochoeris pinnatifida*	.	.	+	+	.	.	.	.	.	.	.	.	.	.	.	.	.	.	.	.	.	.
*Euphorbia falcata*	.	.	.	.	.	.	.	.	.	.	.	.	.	.	.	.	+	1	.	.	.	.
*Aira caryophyllea*	.	.	.	.	.	.	.	.	.	.	.	.	.	1	1	.	.	.	.	.	.	.
*Paronychia echinulate*	.	.	.	+	+	.	.	.	.	.	.	.	.	.	.	.	.	.	.	.	.	.
*Reichardia picroides*	.	.	.	.	.	.	.	.	.	.	.	.	.	.	.	.	.	.	+	+	.	.
*Lotus edulis*	.	.	.	.	.	.	.	.	.	.	.	.	.	.	.	.	.	.	+	.	+	.
*Hypochoeris achyrophorus*	.	.	.	.	.	.	.	.	.	.	.	.	.	.	.	.	.	.	+	.	+	.
*Ferula arrigonii*	.	.	.	.	.	.	.	.	.	.	.	.	.	.	.	.	.	.	+	+	.	.
*Avena barbata*	.	.	.	.	.	.	.	.	.	.	.	.	.	.	.	.	.	.	+	.	+	.
*Sagina hawaiensis*	.	.	+	.	+	.	+	.	.	.	.	.	.	.	.	.	.	.	.	.	.	.
*Montia arvensis*	.	.	.	.	.	3	2	.	.	.	.	.	.	.	.	.	.	.	.	.	.	.
*Dittrichia graveolens*	.	.	.	.	.	.	.	.	.	.	.	.	+	.	.	.	+	.	.	.	.	.
*Triglochin barrelieri*	.	.	.	.	.	.	.	.	.	.	+	.	.	.	.	+	.	.	.	.	.	.
*Carex flacca* subsp. *serrulata*	.	.	+	+	.	.	.	.	.	.	.	.	.	.	.	.	.	.	.	.	.	.
*Selaginella denticulata*	.	.	+	+	.	.	.	.	.	.	.	.	.	.	.	.	.	.	.	.	.	.
*Trifolium leucanthum*	.	.	+	+	.	.	.	.	.	.	.	.	.	.	.	.	.	.	.	.	.	.
*Aira cupaniana*	.	.	.	+	+	.	.	.	.	.	.	.	.	.	.	.	.	.	.	.	.	.
*Galium murale*	.	.	+	.	+	.	.	.	.	.	.	.	.	.	.	.	.	.	.	.	.	.
*Blackstonia perfoliate*	.	.	1	+	.	.	.	.	.	.	.	.	.	.	.	.	.	.	.	.	.	.
*Teesdalia nudicaulis*	.	.	.	.	.	+	+	.	.	.	.	.	.	.	.	.	.	.	.	.	.	.
*Sedum glandulosum*	.	.	.	.	1	.	.	.	.	.	.	.	.	.	.	.	.	.	.	.	.	.
*Anthoxanthum ovatum*	.	.	.	.	2	.	.	.	.	.	.	.	.	.	.	.	.	.	.	.	.	.
*Bromus hordeaceus*	.	.	.	.	1	.	.	.	.	.	.	.	.	.	.	.	.	.	.	.	.	.
*Allium subhirsutum*	.	.	+	.	.	.	.	.	.	.	.	.	.	.	.	.	.	.	.	.	.	.
*Leontodon tuberosum*	.	.	+	.	.	.	.	.	.	.	.	.	.	.	.	.	.	.	.	.	.	.
*Asterolinon linum-stellatum*	.	.	+	.	.	.	.	.	.	.	.	.	.	.	.	.	.	.	.	.	.	.
*Centaurium pulchellum*	.	.	.	+	.	.	.	.	.	.	.	.	.	.	.	.	.	.	.	.	.	.
*Gastridium scabrum*	+	.	.	.	.	.	.	.	.	.	.	.	.	.	.	.	.	.	.	.	.	.

**Localities and dates of relevés**: Rel. 1–2, Monte Arcosu, 6 June 1999; Rel. 3–4, Baccu Locci (CA). 26 May 2002; Rel. 5, Monte Cardiga, 26 May 2002; Rel. 6–7, Spring Sa Scovera, Gonnosfanadiga, 12 June 1998; Rel. 8–18, La Maddalena Archipelago, Biondi & Bagella (2005), Tab. 11; Rel. 19–22, La Maddalena Island, Biondi & Bagella (2005), Tab. 12.

Syn.: *Romuleo requienii-Colchicetum corsicae* Biondi & Bagella 2005, Fitosociologia 42 (2) Suppl. 1: 16

Characteristic species: *Bellium bellidioides*, *Romulea requienii*, *Crocus minimus*, *Colchicum verlaqueae* (=*C. corsicum* auct. non Baker).

Structure and ecology: The association is usually localized in small, humid pools, already drying up at the beginning of spring. It is mainly linked to siliceous substrates (granites and metamorphic rocks), physiognomically characterized by the dominance of *Bellium bellidioides*, which grows together with small geophytes endemic to Sardinia and Corsica, such as *Romulea requienii*, *Colchicum verlaqueae,* and *Crocus minimus*. For its floristic set and ecological requirements, the association must be included in the *Cicendio-Solenopsion laurentiae* rather than in the *Isoetion* alliance, as previously proposed by [21]. Moreover, the *Romuleo requienii-Colchicetum corsicae* Biondi & Bagella 2005 surveyed on deeper soils, should be included within this association, since it represents effectively a xeric variant, where *Colchicum verlaqueae* (sub *C. corsicum*) is abundant while the hygrophytes of the *Isoëto-Nanojuncetea* class are often rare.

Geographical distribution: This association was described from the Maddalena Archipelago, but according to new records, it also occurs in some localities of Southern Sardinia.

#### 2.3.27. *Romuleo requienii-Kickxietum cirrhosae* Brullo, Bacch., Giusso & Miniss. ass. nov. (Table 30)

Holotypus: rel. 2, tab. 30.

**Table 30:** *Romuleo requienii-Kickxietum cirrhosae* Brullo et al. ass. nov.

Relevè Number	1	2 *	3	4	5	6	7	8	9	10	11	12	13	14	15	16	17	18	19	20
Altitude (m)	10	120	120	120	120	120	120	120	120	60	50	40	50	50	40	40	50	50	50	50
Surface (m^2^)	1	1	1	1	1	1	1	1	1	1	1	1	1	1	1	1	1	1	1	1
Coverage (%)	90	80	90	100	80	100	90	100	100	90	80	80	80	90	80	90	90	70	90	90
** Char. Association**																				
*Romulea requienii*	2	1	2	1	1	+	1	1	1	2	1	2	2	2	2	2	2	2	1	2
*Kickxia cirrhosa*	2	2	.	.	1	1	1	+	1	1	1	2	1	1	+	+	1	+	+	.
** Char. *Cicendio-Solenopsion laurentiae***																				
*Anagallis parviflora*	2	2	3	2	2	2	3	2	2	2	2	2	3	2	1	2	1	2	2	3
*Solenopsis laurentia*	1	2	3	3	3	5	2	4	3	1	2	2	1	3	2	2	2	1	3	1
*Cicendia filiformis*	1	2	2	3	2	2	2	2	2	2	1	+	2	.	1	+	.	+	1	2
*Lysimachia minima*		1	+	.	1	+	1	1	1	.	.	3	2	2	2	2	1	2	3	3
*Radiola linoides*	3	+	+	.	.	+	1	3	2	.	1	2	2	3	2	3	4	2	3	3
*Ophioglossum lusitanicum*		.	.	.	+	+	1	.	.	.	1	.	.	.	+	.	.	+	+	.
*Illecebrum verticillatum*		+	.	.	.	.	.	.	.	.	.	.	.	.	.	.	.	.	.	.
** Char.** ***Isoë*** ***tetalia* and ** ***Isoë*** ***to-Nanojuncetea***																				
*Juncinella capitata*	1	2	1	+	+	1	2	2	2	1	1	1	+	+	+	1	2	1	1	+
*Lythrum hyssopifolia*	1	1	1	1	+	2	2	+	1	1	1	1	+	1	+	1	+	1	1	+
*Polypogon subspathaceus*	2	3	3	2	3	2	3	2	2	2	3	1	1	1	2	+	.	1	2	1
*Centaurium maritimum*	1	.	1	+	1	+	+	+	+	1	1	1	+	1	2	2	1	2	2	1
*Mentha pulegium*	1	2	2	1	1	2	3	1	2	4	2	.	+	1	1	.	1	1	.	.
*Verojuncus pygmaeus*	1	.	.	1	1	1	+	1	2	2	1	1	.	1	+	1	1	.	.	+
*Agathryon bufonium*	.	1	1	.	2	+	.	+	1	.	.	1	+	1	1	+	.	+	+	.
*Lotus parviflorus*	.	1	1	.	2	+	.	+	1	.	.	1	+	1	1	+	.	+	+	.
*Isolepis cernua*	.	.	1	1	2	+	1	1	1	.	.	.	.	.	.	.	.	.	.	.
*Gaudinia fragilis*	.	.	.	.	1	.	2	.	.	.	.	.	.	.	.	.	.	.	.	.
** Other species**																				
*Plantago coronopus*	.	.	2	1	2	+	1	1	+	2	1	1	1	+	1	1	.	1	+	+
*Bellis annua*	2	.	.	.	.	.	.	.	.	.	.	2	1	2	3	2	2	1	3	3
*Euphorbia exigua*	.	.	+	1	+	+	1	1	+	.	.	.	.	.	.	.	.	.	.	.

**Localities and dates of relevés**: Rel. 1, San Pietro Island, La Punta, 30 April 1995; Rel. 2–9, San Pietro Island, Paradiso, 12 May 1994; Rel. 10–11, San Pietro Island, Crossroad for Spalmatore, 12 May 1994; Rel. 12–20, Porto Scuso, 11 May 1994. The symbol (*) indicates the nomenclatural type as specified in the ICPN code.

Characteristic species: *Romulea requienii*, *Kickxia cirrhosa*.

Structure and ecology: In large basins submerged for long periods by rainwater, until late spring, in correspondence with acidic lava substrates dating back to the Miocene, ephemeral vegetation rich in hygrophytes of the *Isoeto-Nanojuncetea* class occurs. Among them are frequent *Juncinella capitata*, *Lythrum hyssopifolia*, *Polypogon subspathaceus*, *Centaurium maritimum*, *Mentha pulegium*, *Verojuncus pygmaeus*, *Agathyron bufonium*, *Lotus parviflorus*, *Isolepis cernua*, etc. Moreover, the species of *Cicendio-Solenopsion laurentiae* alliance are also well represented, such as *Anagallis parviflora*, *Solenopsis laurentia* subsp. *laurentia*, *Cicendia filiformis*, *Lysimachia minima*, *Radiola linoides*, *Ophioglossum lusitanicum*, etc. Very significant is the occurrence of *Kickxia cirrhosa* usually growing together with *Romulea requienii*, which allows us to differentiate a new association, proposed as *Romuleo requienii-Kickxietum cirrhosae.* It shows some similarities with *Kickxio cirrhosae-Exaculetum pusilli*, which differs in ecological requirements and floristic set. Indeed, the latter is linked to sandy soils and is characterized by the high coverage of *Exaculum pusillum*, a species with a very late life cycle. Another association quite related to the *Romuleo requienii-Kickxietum cirrhosae* is the *Kickxio cirrhosae-Solenopsietum gasparrinii* Brullo & Minissale 1998 corr., recorded from Sicily where it occurs on muddy-sandy soils within the salt marshes, floristically characterized by *Solenopsis laurentia* subsp. *gasparrinii* [15].

Geographical distribution: This association was surveyed in San Pietro Island and the Sardinian coast near Portoscuso.

#### 2.3.28. *Anagallido parviflorae-Molinerielletum minutae* Brullo, Scelsi, Siracusa & Tomaselli 1998, Boll. Acc. Gioenia Sci. Nat. Catania 29: 172 (Table 31)

Holotypus: rel. 4, tab. 1 [75]

**Table 31:** *Anagallido parviflorae-Molinerielletum minutae* Brullo et al., 1998.

Relevè Number	1	2	3
Altitude (m)	980	975	970
Surface (m^2^)	2	2	1
Coverage (%)	80	70	90
** Char. Association**			
*Molineriella minuta*	2	2	3
** Char. *Cicendio-Solenopsion laurentiae***			
*Anagallis parviflora*	3	3	2
*Cicendia filiformis*	1	+	1
*Radiola linoides*	2	2	3
** Char. *Isoëtetalia* and *Isoëto-Nanojuncetea***			
*Isoët* *es durieui*	3	2	2
*Lotus angustissimus*	2	3	2
*Agathryon bufonium*	2	2	1
*Polypogon subspathaceus*	1	+	1
*Juncinella capitata*	2	1	+
*Briza minor*	1	2	1
*Centaurium maritimum*	+	+	1
*Romulea ramiflora*	2	1	1
** Other species**			
*Bellis annua*	2	2	2
*Galium divaricatum*	1	+	1
*Logfia gallica*	+	+	
*Montia arvensis*	+		
*Trifolium campestre*			+
*Trifolium leucanthum*	+		+

**Localities and dates of relevés**: Rel. 1–3, Spring Sa Scovera, Monte Linas, 12 June 1998.

Characteristic species: *Molineriella minuta*.

Structure and ecology: A very specialized vegetation was observed on granite substrate at around 1000 m altitude, where it is localized in small humid depressions, with shallow soils, rich in sand-silt component, flooded by the waters of a nearby spring. Floristically, it differs in the occurrence and often abundance of *Molineriella minuta*, a rather rare species in Sardinia, colonizing habitats referable to the *Isoeto-Nanojuncetea*. This class is represented in these stands by *Isoetes durieui*, *Lotus angustissimus*, *Agathyron bufonium*, *Polypogon subspathaceus*, *Juncinella capitata*, *Briza minor*, *Centaurium maritimum,* and *Romulea ramiflora.* Significantly, it is also the occurrence of some species of the *Cicendio-Solenopsion laurentiae*, such as *Anagallis parviflora*, *Cicendia filiformis,* and *Radiola linoides* for its floristic peculiarities and ecological requirements; this vegetation is to be referred to the *Anagallido parviflorae-Molinellerietum minutae*, association described from the Hyblean plateau (southern Sicily) by [75] where it is localized on basaltic substrata.

Geographical distribution: This association was surveyed at Sa Scovera Spring near Gonnosfanadiga on the Linas Mount (South-West Sardinia).

#### 2.3.29. *Cynosuro polybracteati-Antoxanthetum aristati* Brullo, Bacch., Giusso & Miniss. ass. nov. (Table 32)

Holotypus: rel.1, tab. 32.

**Table 32:** *Cynosuro polybracteati-Antoxanthetum aristati* Brullo et al., ass. nov.

Relevè Number	1 *	2	3	4	5	6	7	8	9 *	10	11	12	13	14	15	16
Altitude (m)	5	5	5	5	5	5	5	1050	1050	1050	1050	1050	1050	1050	1050	950
Surface (m^2^)	1	1	1	2	2	1	2	1	1	1	1	1	1	5	3	1
Coverage (%)	100	100	100	100	100	100	100	60	80	80	60	90	100	100	90	70
** Char. Association**																
*Anthoxanthum aristatum*	4	5	4	4	3	3	2	2	2	2	2	2	2	1	2	2
*Cynosurus polybracteatus*	1	1	+	2	1	.	1	2	2	2	2	2	3	4	2	.
** Char. Subassociation**																
*Trifolium strictum*	3	2	3	3	3	2	3	.	.	.	.	.	.	.	.	.
*Agathryon hybridum*	2	2	2	3	3	3	3	.	.	.	.	.	.	.	.	.
*Gaudinia fragilis*	+	1	1	+	1	1	1	.	.	.	.	.	.	.	.	.
*Hornungia petraea*	.	.	.	.	.	.	.	2	2	1	1	2	+	1	2	1
*Sagina hawaiensis*	.	.	.	.	.	.	.	2	2	2	2	2	1	1	2	2
*Holosteum umbellatum*	.	.	.	.	.	.	.	1	2	1	2	1	1	+	2	1
*Cerastium palustre*	.	.	.	.	.	.	.	1	2	1	1	1	+	1	1	+
*Ranunculus paludosus*	.	.	.	.	.	.	.		2	1	1	1	1	+	1	+
*Sedum glandulosum*	.	.	.	.	.	.	.	+	2	1	1	1	.	2	2	+
*Veronica praecox*	.	.	.	.	.	.	.	1	+	+	+	.	.	+	+	.
** Char. *Cicendio-Solenopsion laurentiae***																
*Eudianthe laeta*	2	2	2	1	1	1	2	.	.	.	.	.	.	.	1	.
*Anagallis parviflora*	2	1	1	2	2	1	1	.	.	.	.	.	.	.	.	.
*Cicendia filiformis*	1	2	2	1	1	1	+	.	.	.	.	.	.	.	.	.
*Solenopsis laurentia* subsp. *laurentia*	1	+	+	1	+	1	.	.	.	.	.	.	.	.	.	.
*Radiola linoides*	.	.	.	.	.	.	.	.	.	.	.	1	3	+	1	1
*Kickxia cirrhosa*	.	+	.	+	+	.	1	.	.	.	.	.	.	.	.	.
*Lysimachia minima*	.	.	.	.	.	.	.	.	.	.	.	.	.	.	2	1
** Char. *Isoëtetalia* and *Isoëto-Nanojuncetea***																
*Juncinella capitata*	1	2	2	2	1	2	2	3	1	2	3	2	2	3	1	2
*Lotus angustissimus*	2	2	1	2	1	2	1	1	+	+	1	+	1	1	2	.
*Romulea ramiflora*	.	.	+	1	+	+	+	.	+	+	+	+	+	1	+	+
*Agathryon bufonium*	.	.	.	.	.	.	.	1	1	2	1	2	1	3	3	3
*Briza minor*	+	+	.	1	1	+	+	.	.	.	.	.	.	.	1	+
*Lythrum hyssopifolia*	.	.	+	.	1	.	+	.	.	.	.	.	.	.	+	.
*Centaurium pulchellum*	.	.	+	.	+	+	+	.	.	.	.	.	.	.	.	.
*Lotus parviflorus*	.	.	.	.	.	.	.	.	.	.	.	2	1	1	1	.
*Mentha pulegium*	.	.	.	.	.	.	.	.	.	.	.	.	.	1	+	.
*Agrostis pourretii*	.	.	.	.	.	.	.	.	.	.	.	.	.	+		2
*Verojuncus pygmaeus*	.	.	.	.	.	.	.	.	.	.	.	.	.	+	+	.
*Hordeum geniculatum*	.	.	.	.	.	.	.	.	.	.	.	.	.	3	.	.
*Isoët* *es iguliana*	.	.	.	.	.	.	.	.	.	.	.	.	.	+	.	.
*Helosciadium crassipes*	.	.	.	.	.	.	.	.	.	.	.	.	.	+	.	.
** Other species**																
*Bellis annua*	.	.	.	.	.	.	.	+	+	+	2	2	+	2	2	1
*Coleostephus myconis*	1	1	2	1	2	2	+	.	.	.	.	.	.	.	.	.
*Linum trigynum*	1	+	+	1	1	1	1	.	.	.	.	.	.	.	.	.
*Spergularia rubra*	.	.	.	.	.	.	.	1	+	+	+	+	.	1	+	.
*Bromus hordeaceus*	.	.	.	.	.	.	.	.	.	.	1	1	2	2	1	.
*Crocus minimus*	.	.	.	.	.	.	.		+	.	+	+	.	+	.	.
*Montia arvensis*	.	.	.	.	.	.	.	1	+	.	+	.	.	.	1	.
*Euphorbia exigua*	+	+	.	+	.	.	.	.	.	.	.	.	.	.	.	.
*Geranium molle*	.	.	.	.	.	.	.	+	.	.	+		+	.	.	.
*Draba verna*	.	.	.	.	.	.	.		+	.	.	.	.	.	1	.
*Bellium bellidioides*	.	.	.	.	.	.	.	+	.	.	.	.	.	.	.	.
*Aphanes* sp.	.	.	.	.	.	.	.	.	.	.	.	.	.	.	1	.
*Aira caryophyllea*	.	.	.	.	.	.	.	.	.	.	.	.	.	.	.	+
*Oenanthe crocata*	.	.	.	.	.	.	.	.	.	.	.	.	.	+	.	.

**Localities and dates of relevés**: Rel. 1–7, Baia del Sole, Olbia, 21 June 1996; Rel. 8–16, Badde Salighes, Marghine Chain, 3 June 2002. The symbol (*) indicates the nomenclatural type as specified in the ICPN code.

Characteristic species: *Anthoxanthum aristatum*, *Cynosurus polybracteatus*.

Structure and ecology: In the wet pools localized on rocky outcrops, with fairly incoherent sandy soil, a vegetation of microphytes was surveyed, in which two annual grasses *Anthoxanthum aristatum* and *Cynosurus polybracteatus*, have their optimum. The species of *Isoeto-Nanojuncetea* are here very frequent, among them in particular *Juncinella capitata*, *Lotus angustissimus*, *Romulea ramiflora*, *Agathyron bufonium*, *Briza minor*, *Lythrum hyssopifolia*, *Centaurium pulchellum*, *Lotus parviflorus.* Therefore, this plant community is proposed as a new association, namely *Cynosuro polybracteati-Antoxanthetum aristati*. Based on field investigations, this association was observed both along the coastal belt and in mountain stands at about 1000 m of altitude. In these two habitats this vegetation shows significant floristic, as well as ecological, differences, which allow to differentiate two well-distinct subassociations. The first one, representing the typical aspect, occurs in places near the sea and is proposed as *Trifolietosum stricti* Brullo et al. subass. nov. (holotypus: rel. 1), which is floristically characterized by *Trifolium strictum*, *Agathryoon hybridum* and *Gaudinia fragilis.* Moreover, the species of *Cicendio-Solenopsion laurentiae*, such as *Eudianthe laeta*, *Anagallis parviflora*, *Cicendia filiformis*, *Solenopsis laurentia* subsp. *laurentia* and *Kickxia cirrhosa* are well represented. The second one, localized in the mountain places, is proposed as *hornungietosum petraeae* Brullo et al. subass. nov. (holotypus: rel. 9), which is characterized by *Hornungia petraea*, *Sagina hawaiensis*, *Holosteum umbellatum*, *Cerastium palustre*, *Ranunculus paludosus*, *Sedum glandulosum*, *Veronica praecox*. Unlike the previous subassociation, the species of *Cicendio-Solenopsion laurentiae* are less frequent, probably concerning the higher altitude, which does not favor the settlement of essentially thermophilous microphytes typical of this alliance.

Geographical distribution: The association seems localized in few stands of North Sardinia.

*NANOCYPERETALIA* Klika 1935, Beih. Bot. Cent.bl. 53: 292, nom. cons. propos. [56].

Syn.: *Nanocypero-Polygonetalia* W. Kock 1926, Jb. St. Gall. Natunviss. Ges. 61:20, nom. rejic. propos.; *Cyperetalia fusci* Müller-Stoll & Pietsch in Lohmeyer et al., 1962, Melhoramento, 15:20; *Cyperetalia fusci* Pietsch 1963, Abh. u. Ber. Naturkundemus 38:3, nom. illeg. (art. 29); *Cicendietalia filiformis* Géhu 1992, Ann. Bot. (Roma) 50: 139, nom. nud. (2b); *Elatino triandrae-Cyperetalia fusci* de Foucault 1988, Dissert. Bot. 121: 78.

Holotypus: *Nanocyperion flavescentis* W. Koch 1926.

Characteristic species: *Centaurium pulchellum*, *Corrigiola litoralis*, *Cyperus fuscus*, *C. michelianus*, *Hordeum marinum*, *Laphangium luteoalbum*, *Peplis portula*, *Plantago intermedia*, *Spergularia rubra*.

Structure and ecology: This syntaxon groups ephemeral vegetation localized in wide wet hollows usually flooded until early summer, with soils mostly eutrophic or sub-eutrophic, often hypertrophic, usually well-nitrified since they are used as pastures, though occasionally oligo-mesotrophic. Floristically, it is differentiated by the occurrence of species with summer-autumn blooming, showing usually a prostrate and creeping habit. As regards nomenclatural and syntaxonomic remarks, please refer to the notes reported for this order by [15].

Geographical distribution: This order is distributed in the Atlantic and central European territories, extending to the Mediterranean ones with a Temperate bioclimate. In these areas, it can be found in coastal sites with slightly brackish soils, along the shores of artificial basins, and in mountain sites.

*VERBENION SUPINAE* Slavnić 1951, Arch. Sci. Mat. Srpska Sci. Nat. 1: 146.

Syn.: *Heleochloion* Br.-Bl. 1952, Group. Vég. Fr. Médit.: 72; *Fimbristylion dichotomae* Horvatić 1954, Vegetatio 5: 448; *Dichostylidion micheliani* Horvatić 1963, Acta Biol. 4: 37; *Heleochloo-Cyperion micheliani* Pietsch et Müller-Stoll 1968, Mitt. Flor.-Soz. Arbeitsgem. nf. 13: 28.

Lectotypus: *Heliotropio-Verbenetum supinae* Slavnić 1951.

Characteristic species: *Coronopus squamatus*, *Sporobolus aculeatus*, *S. alopecuroides*, *S. schoenoides*, *Euphorbia chamaesyce*, *Glinus lotoides*, *Gnaphalium uliginosum* var. *prostratum*, *Heliotropium supinum*, *Hordeum geniculatum*, *Paspalum distichum*, *Pulicaria sicula*, *P. vulgare*, *Schenkia spicata*, *Verbena supina*.

Structure and ecology: This alliance groups the ephemeral vegetation occurring in wide depressions, represented by lagoons, lakes, artificial basins, river banks, etc., which are subjected to long periods of submersion, usually until early summer. These habitats are often characterized by nitrified soils and flooded by eutrophic or hypertrophic water. They usually host prostrate-creeping species, often of large size, having a summer–autumnal blooming period.

Geographical distribution: The communities of this alliance are spread in Western and Central-Eastern Europe and also in the Mediterranean area.

#### 2.3.30. *Glino lotoidis-Verbenetum supinae* Rivas Goday 1964, Veg. fl. Cuenca extr. Guadiana: 187 (Table 33)

**Table 33:** *Glino lotoides-Verbenetum supinae* Rivas Goday 1964.

Relevè Number	1	2	3	4	5
Altitude (m)	350	350	350	350	350
Surface (m^2^)	10	10	10	20	10
Coverage (%)	50	40	40	50	30
** Char. Association**					
*Glinus lotoides*	3	2	3	3	2
** Char. *Verbenion supinae***					
*Verbena supina*	2	1	1	1	1
*Sporobolus aculeatus*	+	+	+	+	1
*Gnaphalium uliginosum* var. *prostratum*	1	1	+	1	1
*Euphorbia chamaesyce*	.	.	+	+	+
*Coronopus squamatus*	.	.	.	.	+
** Char. *Nanocyperetalia flavescentis***					
*Corrigiola litoralis*	2	2	2	3	2
*Spergularia rubra*	+	+	1	+	+
*Cyperus fuscus*	.	.	.	.	+
*Plantago intermedia*	.	.	+	.	.
** Char. *Isoëto-Nanojuncetea***					
*Mentha pulegium*	1	.	.	+	.
** Other species**					
*Amaranthus blitum*	2	2	1	1	+
*Medicago polymorpha*	+	2	1	1	+
*Paspalum distichum*	+	1	1	1	+
*Polygonum aviculare*	+	+	1	1	+
*Amaranthus graecizans*	+	+	1	1	.
*Oxybasis chenopodioides*	+	1	.	1	1
*Portulaca oleracea*	+	+	1	+	.
*Cyperus rotundus*	+	+	+	.	.
*Medicago arabica*	+	+	+	.	.
*Medicago truncatula*	.	+	+	+	.
*Xanthium spinosum*	1	.	+	+	.
*Solanum nigrum*	.	.	+	+	.
*Astragalus hamosus*	.	+	.	.	.
*Convolvulus arvensis*	+	.	.	.	.
*Erodium cicutarium*	.	.	+	.	.
*Lotus pedunculatus*	.	.	.	+	.
*Malva sylvestris*	.	.	+	.	.
*Echinochloa colona*	.	+	.	.	.
*Sonchus asper*	+	.	.	.	.

**Localities and dates of relevés**: Rel. 1–5, Bidighinzu Lake (Thiesi), 7 October 1997.

Syn.: *Glino-Heliotropietum supini* Brullo & Marcenò 1974 *glinetosum* Brullo & Marcenò 1974, Lav. Ist. Bot. Giard, Col. Palermo 25: 190.

Lectotypus: rel. 1, tab., pg. 188 Rivas Goday [76], designated by Silva et al. [77].

Characteristic species: *Glinus lotoides.*

Structure and ecology: This association was found along the banks of some artificial basins, especially on surfaces that are periodically submerged (from late autumn to early winter and from early to mid-summer) and that preserve wet soil until early autumn. It is differentiated by some hygro-nitrophilous species showing a creeping habit, such as *Glinus lotoides*, *Verbena supina,* and *Corrigiola litoralis*. Among the characteristics of the order and alliance are *Sporobolus aculeatus*, *Gnaphalium uliginosum* var. *prostratum*, *Euphorbia chamaesyce*, and *Spergularia rubra*. It was described by [76] from Spain and recorded by [12] from Sicily too. Previously, this plant community was described by [78] as subass. *glinetosum* of the *Glino-Heliotropietum supini*, proposed as a new association.

Geographical distribution: This association, hitherto recorded only in Spain and Sicily [12], was surveyed from North-West Sardinia, where it occurs in the artificial lake of Bidighinzu near Thiesi.

#### 2.3.31. *Sporobolo aculeati-Eriyngietum pusilli* Brullo, Bacch., Giusso & Miniss. ass. nov. (Table 34)

Holotypus: rel. 4, tab. 34.

**Table 34:** *Sporobolo aculeati-Eryngietum pusilli* Brullo et al. ass. nov.

Relevè Number	1	2	3	4 *	5	6	7	8	9 *
Altitude (m)	350	350	350	350	350	350	350	350	350
Surface (m^2^)	5	5	10	5	5	10	10	10	20
Coverage (%)	60	60	70	60	70	50	60	80	90
** Char. Association**									
*Eryngium pusillum*	4	2	3	4	4	3	2	3	5
*Sporobolus aculeatus*	+	+	1	2	1	2	2	2	2
** Char. *Verbenion supinae***									
*Gnaphalium uliginosum* var. *prostratum*	2	1	2	2	1	1	+	1	+
*Verbena supina*	2	+	2	1	.	1	1	2	1
*Glinus lotoides*	1	1	1	+	+	.	.	.	.
*Coronopus squamatus*	.	.	.	.	.	2	1	2	1
*Hordeum geniculatum*	.	.	.	.	.	1	1	+	+
*Euphorbia chamaesyce*	.	.	.	.	.	.	+	+	.
** Char. *Nanocyperetalia flavescentis***									
*Corrigiola litoralis*	3	2	2	2	2	2	3	2	1
*Spergularia rubra*	1	+	+	1	+	2	2	1	.
** Char. *Isoëto-Nanojuncetea***									
*Mentha pulegium*	2	1	3	2	2	1	2	2	2
*Pulicaria vulgaris*	.	.	.	.	.	+	.	.	+
** Other species**									
*Polygonum aviculare*	+	1	+	1	1	2	1	1	1
*Paspalum distichum*	1	2	2	2	1	2	2	2	+
*Lotus pedunculatus*	+	+	+	+	+	1	+	+	.
*Oxybasis chenopodioides*	+	1	+	+	+	.	+	+	.
*Potentilla reptans*	1	1	1	1	1	.	.	1	1
*Trifolium striatum*	+	2	+	+	1	+	.	+	.
*Xanthium spinosum*	1	2	.	.	+	1	1	1	+
*Medicago polymorpha*	+	1	.	+	.	+	+	1	.
*Portulaca oleracea*	1	+	.	.	.	+	+	+	+
*Amaranthus blitum*	1	1	.	+	.	.	.	1	.
*Convolvulus arvensis*	.	+	.	.	.	.	.	+	+
*Sonchus asper*	.	.	.	.	.	+	.	+	+
*Cynodon dactylon*	.	.	.	.	.	+	.	1	.
*Medicago truncatula*	.	.	.	.	.	.	.	+	+
*Symphyotrichum squamatum*	+	.	.	.	.	.	.	.	.

**Localities and dates of relevés**: Rel. 1–5, Bidighinzu Lake (Thiesi), 6 October 1997; Rel. 6–9, Bidighinzu Lake (Thiesi), 7 October 1997. The symbol (*) indicates the nomenclatural type as specified in the ICPN code.

Characteristic species: *Eryngium pusillum*, *Sporobolus aculeatus*.

Structure and ecology: On the surfaces along the banks of artificial lakes subject to more prolonged periods of submersion, with markedly humid and muddy soils, a vegetation with more hygrophilous requirements than *Glino lotoidis-Verbenetum supinae* occurs. It is floristically differentiated by *Eryngium pusillum*, *Sporobolus aculeatus* species showing a fairly late flowering period, which grow together with other species of the alliance and higher rank, such as *Gnaphalium uliginosum* var. *prostratum*, *Paspalum distichum*, *Verbena supina*, *Hordeum geniculatum*, *Euphorbia chamaesyce*, *Corrigiola litoralis*, *Spergularia rubra*, *Mentha pulegium*, etc. It is a plant community floristically well distinct from the other associations known of the *Verbenion supinae*, which is described as a new association, namely *Sporobolo aculeati-Eriyngietum pusilli.* Two subassociations can be distinguished within this association, represented by: (a) *Glinetosum lotoidis* subass. nov. (holotypus: rel. 4) differentiated by *Glinus lotoides* and (b) *Coronopetosum squamati* subass. nov. (holotypus: rel. 9) differentiated by *Coronopus squamatus*, which shows more nitrophilous requirements than the previous one.

Geographical distribution: The association was surveyed in the artificial lake Bidighinzu in North-West Sardinia.

#### 2.3.32. *Veronico beccabungae-Cyperetum fusci* Brullo, Bacch., Giusso & Miniss. ass. nov. (Table 35)

Holotypus: rel. 3, tab. 35.

**Table 35:** *Veronico beccabungae-Cyperetum fusci* Brullo et al. ass. nov.

Relevè Number	1	2	3 *	4	5	6	7	8	9	10
Altitude (m)	350	350	350	350	350	350	350	350	350	350
Surface (m^2^)	5	10	10	10	5	10	10	5	5	10
Coverage (%)	90	80	70	60	90	80	30	50	60	60
** Char. Association**										
*Veronica beccabunga*	2	2	1	2	2	1	1	2	2	2
** Char. *Verbenion supinae***										
*Gnaphalium uliginosum* var. *prostratum*	+	2	1	1	+	1	2	2	3	2
*Glinus lotoides*	.	+	+	+	.	.	+	1	1	.
*Sporobolus aculeatus*	.	+	+	.	.	.	+	.	.	.
*Sporobolus schoenoides*	.	.	.	.	.	.	.	2	2	2
*Coronopus squamatus*	.	+	.	.	.	.	.	.	.	.
** Char. *Nanocyperetalia flavescentis***										
*Cyperus fuscus*	3	3	3	3	4	4	2	2	2	3
*Corrigiola litoralis*	.	+	1	1	+	1	1	+	1	+
*Plantago intermedia*	.	+	+	+	1	1	+	+	1	+
*Peplis portula*	.	.	.	.	.	+	+	+	+	+
*Spergularia rubra*	.	.	+	+	.	.	.	.	+	+
** Char. *Isoëto-Nanojuncetea***										
*Lotus angustissimus*	.	.	.	.	.	+	+	.	+	+
*Agathryon bufonium*	.	.	.	.	+	.	.	.	2	1
** Other species**										
*Apium nodiflorum*	3	3	2	2	3	3	1	1	1	2
*Oxybasis chenopodioides*	1	2	1	2	1	1	1	+	1	1
*Paspalum distichum*	+	1	1	1	+	1	1	1	1	+
*Medicago polymorpha*	+	+	1	+	+	+	.	+	.	+
*Portulaca oleracea*	.	+	+	+	.	+	.	+	+	.
*Persicaria lapathifolia*	+	1	+	.	+	+	+	.	.	.
*Nasturtium officinalis*	.	.	.	+	+	+	.	1	+	+
*Cyperus rotundus*	.	.	+	+	+	.	1	1	.	+
*Samolus valerandi*	.	+	+	+	+	+	.	.	.	.
*Lotus pedunculatus*	.	+	+	.	.	+	.	.	+	+
*Callitriche* sp.	.	+	+	+	+	+	.	.	.	.
*Verbena officinalis*	.	.	.	+	.	.	.	+	+	+
*Sonchus asper*	.	+	.	.	.	.	.	.	.	.
*Solanum nigrum*	.	.	+	.	.	.	.	.	.	.
*Rumex sanguineus*	.	.	+	.	.	.	.	.	.	.
*Ranunculus cordiger* subsp. *diffusus*	.	.	.	.	.	.	.	.	+	.

**Localities and dates of relevés**: Rel. 1–10, Bidighinzu Lake (Thiesi), 7 October 1997. The symbol (*) indicates the nomenclatural type as specified in the ICPN code.

Characteristic species: *Veronica beccabunga*, *Cyperus fuscus*.

Structure and ecology: In stands with surfaces flooded even during the summer-autumn period usually by shallow waters and characterized by muddy soils, an amphibious vegetation, physiognomically differentiated by *Cyperus fuscus* and *Veronica beccabunga*, growing with *Apium nodiflorum* and *Oxybasis chenopodioides* occurs. It is a community with marked hygrophilous requirements belonging to *Verbenion supinae*, as confirmed by the occurrence of *Gnaphalium uliginosum* var. *prostratum*, *Glinus lotoides*, *Sporobolus aculeatus*, *Sporobolus schoenoides*, etc. Based on these peculiarities, it is described as a new association named *Veronico beccabungae-Cyperetum fusci*, which does not seem to have affinity with other associations of this alliance known in the literature.

Geographical distribution: The association was surveyed in the artificial lake Bidighinzu in North-West Sardinia.

#### 2.3.33. *Pulicario vulgaris-Menthetum pulegium* Slavnić 1951, Arch. Sei. Matica Srpska, Ser. Scí. Nat. 1: 147. (Table 36)

Lectotypus: rel. 4, tab. 27 [79], hoc loco.

**Table 36:** *Pulicario vulgaris-Menthetum pulegium* Slavnić 1951.

Relevè Number	1	2	3	4	5	6	7
Altitude (m)	580	580	580	580	580	580	580
Surface (m^2^)	5	5	10	10	5	10	10
Coverage (%)	60	70	80	70	80	80	80
** Char. Association**							
*Pulicaria vulgaris*	2	1	2	2	1	2	2
** Char. *Verbenion supinae***							
*Heliotropium supinum*	+	1	2	2	1	2	3
*Sporobolus schoenoides*	+	+	1	.	+	+	.
*Pulicaria sicula*	.	+	.	+	.	.	.
** Char. *Nanocyperetalia flavescentis***							
*Corrigiola litoralis*	.	.	.	+	2	1	.
*Peplis portula*	.	.	.	.	+	+	.
** Char. *Isoëto-Nanojuncetea***							
*Mentha pulegium*	3	4	4	3	4	3	4
*Eryngium pusillum*	2	2	3	2	2	3	1
*Lythrum hyssopifolia*	+	.	+	.	+	+	+
*Agathryon bufonium*	.	.	.	+	+	.	+
** Other species**							
*Plantago lanceolata*	1	+	1	+	+	1	+
*Baldellia ranunculoides*	.	+	+	.	1	1	+
*Erodium* sp.	.	.	.	+	+	.	.
*Malva* sp.	.	.	.	+	.	+	.

**Localities and dates of relevés**: Rel. 1–7, Giara di Gesturi, 5 October 1997.

Characteristic species: *Pulicaria vulgaris Mentha pulegium*.

Structure and ecology: The association was described by [79] from Serbia and included in the *Verbenion supinae* alliance. It is linked to large wetlands flooded until early summer, with loamy soils, humid for a long time, and more or less rich in nitrogen components, due to grazing. Physiognomically, it is differentiated by the dominance of *Mentha pulegium*, which usually grows together with *Pulicaria vulgaris*, a therophyte with a prostrate habit. Several hygro-nitrophilous species of the alliance and order are quite frequent, such as *Heliotropium supinum*, *Sporobolus schoenoides*, *Pulicaria sicula*, *Corrigiola litoralis*, *Peplis portula*, etc., while the class is represented apart from *Mentha pulegium*, by *Eryngium pusillum*, *Lythrum hyssopifolia,* and *Agathryon bufonium.*

Geographical distribution: According to the literature data [79,80,81,82], this association is spread in Eastern Europe, such as Serbia, Macedonia, the Czech Republic, Romania, and Ukraine. As concerns the Italian territory, it is recorded for the first time, where it occurs Sardinia at the Giara di Gesturi.

#### 2.3.34. *Sporobolo aculeati-Pulicarietum siculae* Brullo, Bacch., Giusso & Miniss. ass. nov. (Table 37)

Holotypus: rel. 6, tab. 37.

**Table 37:** *Sporobolo aculeati-Pulicarietum siculae* Brullo et al. ass. nov.

Relevè Number	1	2	3	4	5	6 *	7	8	9	10	11
Altitude (m)	5	5	5	5	5	5	5	5	5	5	5
Surface (m^2^)	2	2	3	1	2	5	2	2	3	2	3
Coverage (%)	70	90	50	90	90	80	90	90	30	90	90
** Char. Association**											
*Pulicaria sicula*	3	4	3	3	4	4	2	4	1	2	2
** Char. *Verbenion supinae* and *Nanocyperetlia flavescentis***											
*Sporobolus aculeatus*	2	2	1	2	2	2	3	2	+	3	4
*Schenkia spicata*	1	2	1	1	1	1	1	2	1	2	1
*Hordeum geniculatum*	.	.	+	1	.	1	1	1	.	.	.
** Char. *Isoëto-Nanojuncetea***											
*Lythrum hyssopifolia*	3	2	2	4	2	1	3	3	1	2	2
*Isolepis cernua*	.	.	.	.	.	.	.	.	.	3	3
** Other species**											
*Centarium tenuiflorum*	+	.	+	+	.	.	1	.	.	.	.
*Triglochin barrelieri*	+	+	+	1	1	+	1	.	.	.	.
*Atriplex prostrata*	.	.	.	.	.	.	1	1	3	1	.

**Localities and dates of relevés**: Rel. 1–11, Salt marshes of Stintino, 26 September 1992. The symbol (*) indicates the nomenclatural type as specified in the ICPN code.

Characteristic species: *Pulicaria sicula*, *Sporobolus aculeatus* (=*Crypsis aculeata*).

Structure and ecology: In the coastal salt marshes, limited to the surfaces with sandy-loamy soils flooded until the end of spring and quite humid even in the summertime, there is an ephemeral vegetation, linked to a moderate edaphic salinity, in which the dominance of two therophytes with summer–autumnal life cycle, is observed. They are *Pulicaria sicula*, with a scapose-erect habit, and *Sporobolus aculeatus*, a creeping microphyte. Among the *Verbenion supinae* and related class occur *Schenkia spicata*, *Hordeum geniculatum*, *Lythrum hyssopifolia,* and *Isolepis cernua.* For its floristic and ecological peculiarities, this plant community is proposed as a new association named *Sporobolo aculeati-Pulicarietum siculae*. This association shows some similarity for the occurrence of *Pulicaria sicula*, with *Mentho pulegii-Pulicarietum siculae* described by [83] from Corsica and referred to the *Nanocyperetalia* order. Other plant communities characterized by *Sporobolus aculeatus* and referred to as *Lythrion tribracteati* are represented by *Damasonio bourgaei-Crypsietum aculeatae* Rivas-Martínez & Costa in Rivas-Martínez et al., 1980 corr. recorded from Sicily, Malta, Spain, and Tunisia [15,84]. Moreover, several authors [85,86,87] surveyed, from some salt marshes of the Italian Peninsula, the occurrence of *Crypsietum aculeatae* Wenzl 1934, an association included in the class *Crypsietea aculeatae* Vicherek 1973 [88].

Geographical distribution: The association was observed exclusively in the Stintino Peninsula (North-West Sardinia), where it is very rare.

#### 2.3.35. *Cresso creticae-Sporoboletum aculeati* Brullo, Bacch., Giusso & Miniss. ass. nov. (Table 38)

Holotypus: rel. 1, tab. 38.

**Table 38:** *Cresso creticae-Sporoboletum aculeati* Brullo et al. ass. nov.

Relevè Number	1 *	2	3	4	5	6	7
Altitude (m)	5	5	5	5	5	5	5
Surface (m^2^)	1	2	2	1	1	1	1
Coverage (%)	90	80	70	60	80	60	90
** Char. Association**							
*Cressa cretica*	2	4	4	3	3	1	2
** Char. *Verbenion supinae* and *Nanocyperetalia flavescentis***							
*Sporobolus aculeatus*	4	2	2	2	3	3	4
*Pulicaria sicula*	1	.	.	.	.	.	.
*Schenkia spicata*	.	.	.	.	.	+	.
** Char. *Isoëto-Nanojuncetea***							
*Lythrum hyssopifolia*	3	2	1	1	2	1	1
** Other species**							
*Atriplex prostrata*	1	1	+	1	+	3	3
*Frankenia pulverulenta*	.	.	.	.	.	1	1

**Localities and dates of relevés**: Rel. 1–7, Salt marshes of Stintino, 26 September 1992. The symbol (*) indicates the nomenclatural type as specified in the ICPN code.

Characteristic species: *Cressa cretica*, *Sporobolus aculeatus*.

Structure and ecology: The *Sporobolo aculeati-Pulicarietum siculae* is replaced in the salt marshes limited to stands with soil having higher chloride concentrations by a plant community always dominated by *Sporobolus aculeatus*, but with the occurrence of *Cressa cretica*, while *Pulicaria sicula* is almost completely missing. Therefore, it is proposed as a new association, namely *Cresso creticae-Sporoboletum aculeati.* Among the species of *Isoeto-Nanojuncetea* only *Lythrum hyssopifolia* shows a constant frequency.

Geographical distribution: The association was observed exclusively in the Stintino Peninsula (North-West Sardinia), where it is very rare.

## 3. Materials and Methods

### 3.1. Study Area

**Figure 3:**
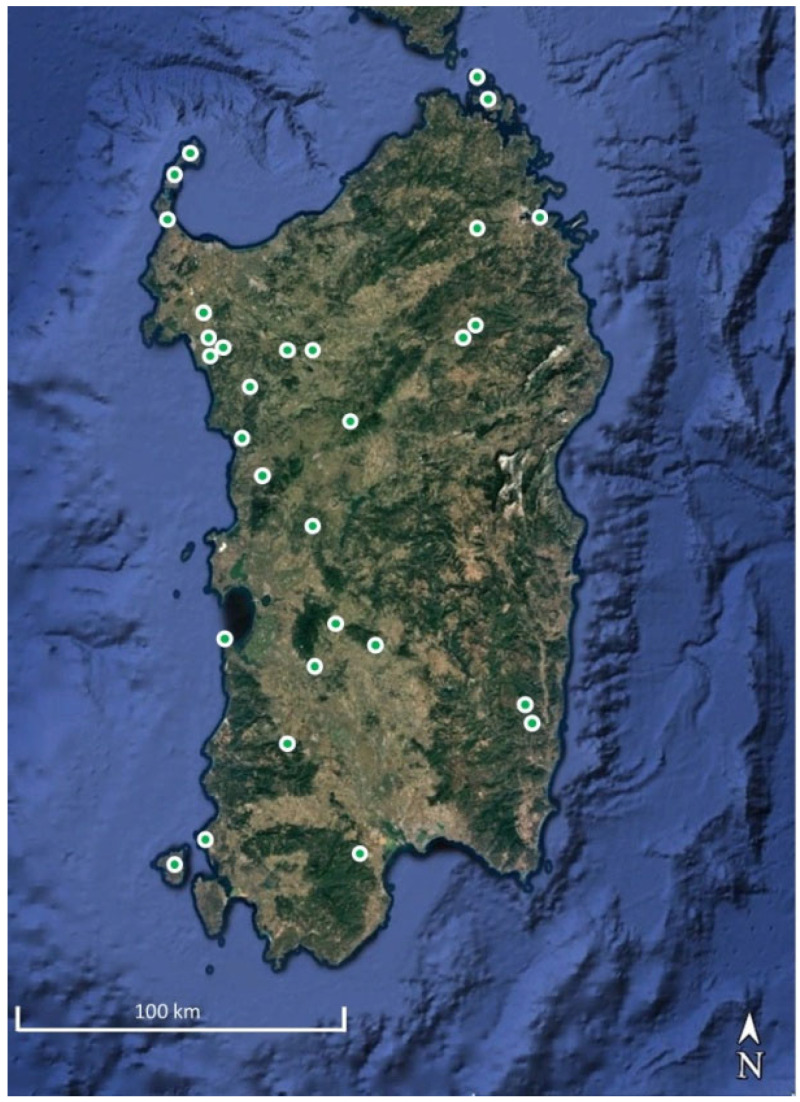
Map of Sardinia with the localities where the relevés were carried out (circles), from Google Earth (modified).

The research covers the whole of Sardinia, an island located in the western Mediterranean (Figure 3) which is the second largest one among those present in this area with a surface of 24,090 km^2^ (including the smaller islands) and with a coastline 1897 km long. Sardinia is separated to the north from Corsica by the Strait of Bonifacio, to the east is surrounded by the Tyrrhenian Sea, to west by the Sardinian Sea, and to the south by the Sardinian Channel.

The Sardinian environment is characterized by hilly complexes alternating with mountain ones, within which there are extensive massifs, such as the Gennargentu (Punta La Marmora, 1834 m a.s.l.) and Limbara (Punta Balistréri, 1362 m). Plains and plateaus are also frequent, while along the coasts occur dune systems, low reefs, and cliffs. Geologically, the island is constituted by siliceous, carbonate, marly, and clayey rocks, dating back from the Paleozoic to the Miocene, as well as Quaternary deposits represented by calcarenites, tuffs, conglomerates, dunes, etc. The Tertiary and Quaternary volcanic rocks are quite well represented, especially those of effusive type [89].

According to the Rivas-Martinez approach [90,91] in Sardinia, two main macrobioclimates can be recognized [92]: Mediterranean Pluviseasonal-Oceanic and Temperate oceanic especially in the sub-Mediterranean variant. In particular, the first macrobioclimate is the most widespread on the island, ranging from sea level to about 1200–1300 m of altitude, while five thermotypic horizons (from lower Thermomediterranean to lower Supramediterranean) and five ombrothermic horizons (from lower dry to lower humid) can be recognized. As concerns the second macrobioclimate, it is circumscribed to the top of Gennargentu and Limbara with a hint also on Mt. Rasu (Goceano Range); within it, three thermotypic horizons can be identified (from upper Mesotemperate to upper Supratemperate) with three ombrothermic horizons (from lower humid to lower hyperhumid).

Based on the distribution of endemic plants in Sardinia and its geological and geomorphological features, six main biogeographical sectors were recognized, with a further subdivision into 22 sub-sectors [93]. These sectors were arranged within the Sardinian subprovince, included in the Cyrno-Sardinian and Tuscan Archipelago province [94], part of the Italo-Tyrrhenian superprovince, which extends over the western coast of the Italian Peninsula from Liguria to Calabria.

The environments having a great relevance for the investigations concerning this study, are the wetlands that in Sardinia are represented rarely by natural lakes (Baratz Lake near Porto Ferro and Piscina Morta near Fluminimaggiore), but above all by artificial basins (Omodeo, Oschiri, Mulargia, Monteleone, Bidighinzu, etc.) and watercourses (Cedrino, Coghinas, Flumendosa, Liscia, Posada, Temo, Tirso, etc.). Much more significant for our research are the temporary ponds, locally called “pauli”, which are widespread throughout the whole island and also on some nearby islets. The most important and floristically relevant temporary ponds occur at the Giara di Gesturi, Paulilatino, Altopiano di Campeda, Buddusò, Badde Salighes, Alà dei Sardi, Stintino, Monte Rosso, Monte Miale Ispina, and in the islands of Asinara, Maddalena, Caprera, and San Pietro.

**Figure 4:**
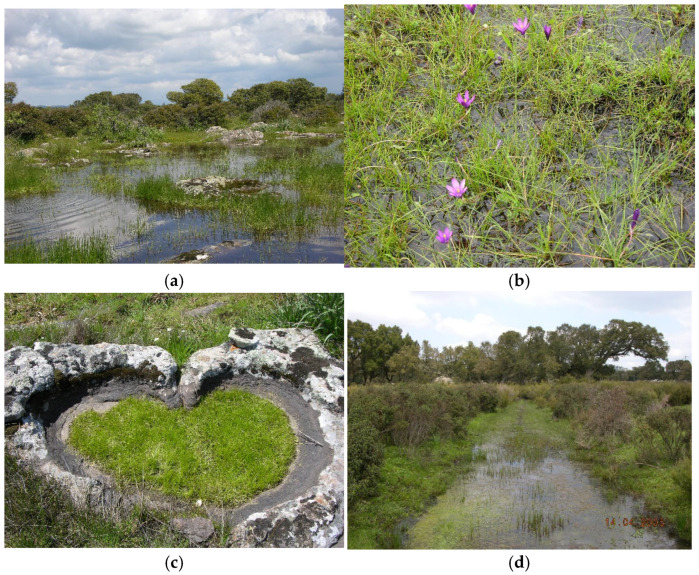
Different typologies of Mediterranean temporary ponds: (**a**) Paule, (**b**) waterlogged soil, (**c**) rock pool, (**d**) ditch.

In Sardinia, temporary ponds are frequent from the coastal belt to the mountain one, up to about 1000 m. The most floristically relevant communities occur on granitic and volcanic substrates in the plateaus, where wooded grasslands dominate a landscape related to the millennia of pastoral activities. Hydromorphic soils with clay texture and slow drainage favored flooding during the winter–spring period, gradually drying up in the summer. The main typologies of Mediterranean temporary ponds in Sardinia are large depressions named “pauli”, (Figure 4a), small depressions or waterlogged soils (Figure 4b) in patchwork with shrub vegetation dominated by *Myrtus communis,* and rocky pools (Figure 4c) on rocky outcrops [23]. They can also appear in the shoreline of artificial basins, ditches (Figure 4d), and temporary watercourses.

### 3.2. Floristic Considerations

The Sardinian wetlands, colonized by the plant communities belonging to the *Isoëto-Nanojuncetea* class, are characterized by a well-specialized flora, very rich in hygrophilous species usually showing a remarkable taxonomical and phytogeographical significance (Appendix A). In these habitats, there are several therophytes and cryptophytes (geophytes and hemicryptophytes), usually having a small size [95]. In particular, some rare Sardinian endemics are worth mentioning, such as *Colchicum verlaqueae*, *Ranunculus cordiger* subsp. *diffusus*, *Crocus minimus*, *Romulea requienii* having usually a sporadic or scattered occurrence [96].

Instead, many other specialized Mediterranean species are quite frequent, such as *Agrostis pourretii*, *Anthoxanthum aristatum*, *Cynosurus polybracteatus*, *Elatine campylosperma*, *Gnaphalium uliginosum* var. *prostratum*, *Helosciadium crassipes*, *Isoëtes durieui*, *I. gymnocarpa*, *I. histrix*, *I. longissima*, *I. tiguliana*, *Lotus conimbricensis*, *Lythrum tribracteatum*, *Molineriella minuta*, *Myosotis sicula*, *Pilularia minuta*, *Ranunculus revelieri*, *Trifolium michelianum*, etc. Among the species having a wider Mediterranean, Mediterranean-Atlantic or Euro-Mediterranean distribution can be observed in these habitats also *Anagallis parviflora*, *Antinoria insularis*, *Bulliarda vaillantii*, *Centaurium maritimum*, *Cicendia filiformis*, *Corrigiola litoralis*, *Damasonium bourgaei*, *Elatine macropoda*, *Eryngium pusillum*, *Eudianthe laeta*, *Euphorbia falcata*, *Exaculum pusillum*, *Gaudinia fragilis*, *Illecebrum verticillatum*, *Juncinella capitata*, *Kickxia cirrhosa*, *Lotus angustissimus*, *L. hispidus*, *L. parviflorus*, *Mentha pulegium*, *Middendorfia borysthenica*, *Polypogon subspathaceus*, *Pulicaria sicula*, *Ranunculus ophioglossifolius*, *R. saniculifolius*, *R. trilobus*, *Romulea ramiflora*, *Solenopsis laurentia* subsp. *laurentia*, etc. Finally, it is possible to find many other species with a large range, chiefly represented by paleotemperate, circumboreal, paleotropical, subcosmopolitan and cosmopolitan elements, e.g., *Briza minor*, *Coronopus squamatus*, *Cyperus fuscus*, *Glinus lotoides*, *Heliotropium supinum*, *Isolepis cernua*, *Agathyron bufonium*, *A. tenageia*, *Laphangium luteo-album*, *Lysimachia minima*, *Lythrum hyssopifolia*, *Mentha pulegium*, *Ophioglossum lusitanicum*, *Peplis portula*, *Plantago intermedia*, *Poa infirma*, *Pulicaria vulgaris*, *Radiola linoides*, *Ranunculus lateriflorus*, *Sporobolus aculeatus*, *S. schoenoides*, *Trifolium micranthum*, *Verbena supina*, etc.

### 3.3. Syntaxonomical Considerations on Isoëto-Nanojuncetea Class

As regards the syntaxonomic treatment of the plant communities referable to the *Isoëto-Nanojuncetea* class, it must be emphasized that, on the basis of the vast literature concerning this vegetation [15], there are often quite conflicting opinions which have led the various phytosociologists to propose frameworks over time often very divergent from each other. Previously, a very detailed chronicle on this topic was addressed by [15], who examined the various schemes proposed in the literature, clarifying from a nomenclatural, floristic, ecological, phenological, and chorological profile, the meaning of the higher-rank syntaxa known so far. Based on these investigations, they proposed for the Italian territory the following syntaxonomic scheme that provides a realistic overview of this vegetation.
 ISOËTO-NANOJUNCETEA Br.-Bl. & R. Tx. ex Westhoff, Dijk & Passchier 1946  ISOËTETALIA Br.-Bl. 1936 nom. conserv. propos.   *ISOËTION* Br.-Bl. 1936   *MENTHION CERVINAE* Br.-Bl. ex Moor 1937 nom. mut.   *APIENION CRASSIPEDIS* Bagella et al., 2009   *CICENDIO-SOLENOPSION LAURENTIAE* Brullo & Miniss. 1998   *AGROSTION POURRETII* Rivas Goday 1958 nom. mut.  NANOCYPERETALIA Klika 1935 nom. cons. propos.   *NANOCYPERION FLAVESCENTIS* Koch 1926   *ELOCHARITION SOLONIENSIS* Philippi 1968   *CICENDION* Rivas Goday in Rivas Goday & Borja (1961)   *VERBENION SUPINAE* Slavnić 1951   *LYTHRION TRIBRACTEATI* Rivas Goday & Rivas-Mart. ex Rivas Goday 1970

### 3.4. Dataset

The study spans approximately 30 years and is based on 520 phytosociological relevés carried out using the Braun-Blanquet method [97], with 192 sourced from literature data and 328 unpublished. The relevés were carried out in several Sardinian natural areas, such as La Maddalena Archipelago, Asinara Island, Stintino Peninsula, San Pietro Island, Giara di Gesturi, Olmedo, Badde Salighes, Monte Minerva, Suni, Mogoro, Scanu, Paulilatino, Valverde, Olbia, Monte Cardiga, Alà dei Sardi, Scala Picada, Buddusò, Ardara, Usellus, Capo Frasca, Monte Arcosu, Baccu Locci, Gonnosfanadiga, Portoscuso, Monte Linas, Bidighinzu Lake (Figure 3). The nomenclature of the surveyed syntaxa follows the 4th edition of the International Code of Phytosociological Nomenclature (ICPN) [98], while the syntaxonomical arrangement follows partially [12,15,52,53,61,99].

### 3.5. Floristic Nomenclature

As concerns the floristic nomenclature and life form, we have followed [63,65]. For the genus *Juncus,* we followed a recent revision [100]. The checklist of the species occurring in the phytosociological relevés is reported in Appendix A. Chorological types follow [101].

### 3.6. Data Analysis

In order to verify the syntaxonomical relations among the surveyed plant communities, some relevés (up to five based on availability) were selected for each vegetation type choosing the most typical and floristically rich ones, and the cover-abundance values following the scale of [97], which were transformed according to the method proposed by [102]. Thus, a matrix of 174 relevés × 176 species was selected from the original dataset and subjected to multivariate analysis, after removing species with a frequency lower than 1% limited to the “other species” group of each relevés, as they are mostly accidental. Hierarchical clustering on the final matrix was performed by using flexible beta linkage, with the Bray-Curtis coefficient. Beta was set at −0.25, so flexible beta clustering became a space-conserving method [103]. To determine the optimal number of clusters, we have used the “Optimclass 1” method (*p* < 10^−6^) [59], applying the function “Crispness of Classification” to each data set partition. Hierarchical clustering was run by PCOrd version 6.08. Optimclass was performed by software JUICE [104].

## 4. Conservation Remarks and Conclusions

Our survey allowed us to highlight the great diversity of the plant communities belonging to the *Isoeto-Nanojuncetea* class occurring in Sardinia in different wetlands, such as small rocky pools, large ponds, or banks of artificial basins. In Sardinia, they are spread across several substrata, represented by limestones, dolomites, vulcanites, granites, schists, marls, clays, etc. Although we do not have a detailed mapping, on the basis of our expert-based we can affirm that most of the detected plant communities fall into protected areas such as regional parks, nature reserves and Natura 2000 sites, but this is not enough to guarantee their real protection, because their existence depends above all on good land management and often on a delicate balance between grazing and agro-silvopastoral activities [105]. All the plant communities of the *Isoëto-Nanojuncetea* treated here, due to their attribution to Mediterranean temporary ponds, can be referred to the habitats of community interest codified as 3170* and 3120 according to the Annex II of the Habitat Directive (92/43/EEC), whereby they require rigorous protection by the European Union member states [16]. The criticality of this type of habitat, being temporary ponds, is above all linked to the fact that they are often limited to small surfaces, apart from having a very fragmented distribution. All this tends to make them less visible and not give them sufficient importance; therefore, they are usually neglected. Overall, they are quite vulnerable, even to involuntary destruction [106,107] or to changes in land use, which together can contribute to the disappearance or alteration of these relevant micro-habitats [108]. Another problem with the protection and management of these habitats is that, in many cases, covering very small surfaces, they escape the cartographic surveys of vegetation on a regional scale [109,110,111]. Even in the inventories of wetlands on a regional or national scale, Mediterranean temporary ponds are largely under-represented, thus limiting the possibilities of protection and correct management [112]. It is therefore understood that there is a need to intensify the regional field surveys to understand the real distribution of this habitat better. We hope that in the future, these results will stimulate adequate research and management policies on these habitats and their conservation. Unfortunately, this cannot be separated from effective and coordinated governance at the national level, based on multiple spatial scales ranging from land use policies to the management of protected areas, agricultural areas, and so on [113].

## Data Availability

The original contributions presented in this study are included in the article. Further inquiries can be directed to the corresponding author.

## Appendix A

Checklist of the taxa occurring in the phytosociological relevés with their life forms and chorotypes.

**Taxon**	**Life Form**	**Chorotype**
*Agathryon bufonium* (L.) Záveská Drábková et Proćków	T scap	Cosmop.
*Agathryon hybridum* (Brot.) Záveská Drábková et Proćków	T scap	Medit.-Atl.
*Agathryon subulatum* (Forssk.) Záveská Drábková et Proćków	G rhiz	S Medit.
*Agathryon tenageia* (Ehrh. ex L.f.) Záveská Drábková et Proćków	T caesp	Paleotemp.
*Agrostis pourretii* Willd.	T scap	W Medit.
*Aira caryophyllea* L.	T scap	Subtrop.
*Aira cupaniana* Guss.	T scap	W Medit.
*Aira elegans* Willd.	T scap	Euro-Medit.
*Allium subhirsutum* L.	G bulb	Medit.
*Alopecurus bulbosus* Gouan	H caesp	Euro-Medit.
*Alopecurus geniculatus* L.	H caesp	Subcosmop.
*Amaranthus blitum* L.	T scap	Cosmop.
*Amaranthus graecizans* L.	T scap	Paleotemp.
*Anacamptis longicornu* (Poir.) R.M.Bateman et al.	G bulb	W Medit.
*Anagallis arvensis* L.	T scap	Paleotemp.
*Anagallis foemina* Mill.	T rept	Medit.
*Anagallis latifolia* L.	T rept	Euro-Medit.
*Anagallis parviflora* Hoffmanns. et Link.	T scap	W Medit.
*Anthemis arvensis* L.	T scap	Euro-Medit.
*Anthoxanthum aristatum* Boiss.	T scap	W Medit.
*Anthoxanthum ovatum* Lag.	T scap	W Medit.
*Antinoria insularis* Parl.	T scap	W Medit.
*Archidium alternifolium* (Hedw.) Mitt.	Bryo.	Europ.
*Asphodelus ramosus* L.	G rhiz	Medit.
*Asterolinon linum-stellatum* (L.) Duby	T scap	Medit.
*Astragalus hamosus* L.	T scap	Medit-Iran-Turan.
*Atriplex prostrata* Boucher ex DC.	T scap	Circumbor.
*Avena barbata* Pott ex Link	T scap	Medit-Iran-Turan.
*Baldellia ranunculoides* (L.) Parl.	I rad	Medit.-Atl.
*Bellardia trixago* (L.) All.	T scap	Euro-Medit.
*Bellardia viscosa* (L.) Fisch. et C.A.Mey.	T scap	Medit.-Atl.
*Bellis annua* L.	T scap	Medit.
*Bellium bellidioides* L.	H ros	W Medit.
*Blackstonia perfoliata* (L.) Huds.	T scap	Euro-Medit.
*Briza minor* L.	T scap	Subcosmop.
*Bromus hordeaceus* L.	T scap	Subcosmop.
*Bulliarda vaillantii* (Willd.) DC.	T scap	Euro-Medit.
*Callitriche brutia* Petagna	I rad	Medit.-Atl.
*Callitriche* cfr. *platycarpa* Kütz.	I rad	Euro-Medit.
*Callitriche stagnalis* Scop.	I rad	Euro.-Asiat.
*Callitriche truncata* Guss.	I rad	Medit.-Atl.
*Carex divisa* Huds.	H caesp	Paleotemp.
*Carex serrulata* Biv. ex Spreng.	H caesp	Europ.
*Catapodium rigidum* (L.) C.E. Hubb.	T scap	Medit.-Atl.
*Centaurium maritimum* (L.) Fritsch.	T scap	Medit.
*Centaurium tenuiflorum* (Hoffmanns. et Link) Fritsch	T scap	Paleotemp.
*Cerastium glomeratum* Thuill.	T scap	Paleotemp.
*Cerastium palustre* Moris	T scap	End. Sard.
*Chamaemelum fuscatum* (Brot.) Vasc.	T scap	W Medit.
*Cicendia filiformis* (L.) Delarbre	T scap	Medit.-Atl.
*Colchicum verlaqueae* Fridl.	G bulb	End. Sard.
*Coleostephus myconis* (L.) Cass. ex Rchb. f.	T scap	Medit.
*Convolvulus arvensis* L.	G rhiz	Paleotemp.
*Coronopus squamatus* (Forssk.) Asch.	T rept	Euro-Medit.
*Corrigiola litoralis* L.	T rept	Medit.-Atl.
*Corrigiola telephiifolia* Pourr.	H ros	W Medit.
*Cressa cretica* L.	T scap	Subcosmop.
*Crocus minimus* DC.	G bulb	End. Sard.-Cors.
*Cynodon dactylon* (L.) Pers.	H rept	Cosmop.
*Cynosurus echinatus* L.	T scap	Euro-Medit.
*Cynosurus polybracteatus* Poir.	T scap	W Medit.
*Cyperus badius* Desf.	G rhiz	Paleotemp.
*Cyperus fuscus* L.	T caesp	Paleotemp.
*Cyperus rotundus* L.	G rhiz	Subcosmop.
*Damasonium bourgaei* Coss.	I rad	Medit.-Atl.
*Dittrichia graveolens* (L.) Greuter	T scap	Medit-Iran-Turan.
*Dittrichia viscosa* (L.) Greuter	H scap	W Medit.
*Draba verna* L.	T caesp	Circumbor.
*Echinochloa colona* (L.) Link	T scap	Subtrop.
*Echium plantagineum* L.	H bienn	Euro-Medit.
*Echium vulgare* L.	H bienn	Medit.
*Elatine campylosperma*Seub.	I rad	Medit.-Atl.
*Elatine macropoda* Guss.	I rad	Medit.
*Eleocharis palustris* (L.) Roem. et Schult.	G rhiz	Subcosmop.
*Erodium cicutarium* (L.) L’Hér.	T caesp	Subcosmop.
*Eryngium pusillum* L.	H bienn	S Medit.
*Eudianthe laeta* (Aiton) Fenzl	T scap	W Medit.
*Euphorbia chamaesyce* L.	T rept	Euro-Medit.
*Euphorbia exigua* L.	T scap	Euro-Medit.
*Euphorbia falcata* L.	T scap	Medit-Iran-Turan.
*Exaculum pusillum* (Lam.) Caruel	T scap	W Medit.
*Ferula arrigonii* Bocchieri	H scap	End. Sard.-Cors.
*Filago germanica* (L.) Huds.	T scap	Medit.
*Frankenia pulverulenta* L.	T scap	Medit.
*Galactites tomentosus* Moench	T scap	Medit.
*Galium divaricatum* Lam.	T scap	Medit.
*Galium murale* (L.) All.	T scap	Medit.
*Gastridium scabrum* C.Presl	T scap	Medit.
*Gaudinia fragilis* (L.) P. Beauv.	T scap	Euro-Medit.
*Geranium dissectum* L.	T scap	Euro-Medit.
*Geranium molle* L.	T scap	Euro-Asiat.
*Glinus lotoides* L.	T scap	Paleotrop.
*Glyceria spicata* Guss.	I rad	W Medit.
*Gnaphalium uliginosum* L. var. *prostratum* Huet	T scap	End. Ital.
*Hedypnois cretica* (L.) Dum. Cours.	T scap	Medit.
*Heliotropium supinium* L.	T scap	Paleotrop.
*Helosciadium crassipes* W.D.J.Koch ex Rchb.	I rad	Medit.
*Helosciadium nodiflorum* (L.) W.D.J.Koch	I rad	Euro-Medit.
*Holcus lanatus* L.	H caesp	Circumbor.
*Holosteum umbellatum* L.	T scap	Paleotemp.
*Hordeum geniculatum* All.	T scap	Euro-Medit.
*Hordeum marinum* Huds.	T scap	Medit.-Atl.
*Hornungia petraea* (L.) Rchb.	T scap	Medit.
*Hypericum scruglii* Bacch., Brullo et Salmeri	H scap	Endem. Sard.
*Hypochoeris achyrophorus* L.	T scap	Medit.
*Hypochoeris glabra* L.	T scap	Euro-Medit.
*Hypochoeris pinnatifida* Ten.	H scap	Medit.
*Illecebrum verticillatum* L.	T scap	Medit.-Atl.
*Isoëtes durieui* Bory	G bulb	W Medit.
*Isoëtes gymnocarpa* (Gennari) A.Braun	G bulb	S Medit.
*Isoëtes histrix* Bory	G bulb	Medit.-Atl.
*Isoëtes longissima* Bory	G bulb	W Medit.
*Isoëtes tiguliana* Gennari	G bulb	Endem. Sard.
*Isolepis cernua* (Vahl) Roem. et Schult.	T scap	Subcosmop.
*Isolepis setacea* (L.) R.Br.	T scap	Paleotemp.
*Juncinella capitata* (Weigel) Záveská Drábková et Proćków	T scap	Euro-Medit.
*Kickxia cirrhosa* (L.) Fritsch.	T scap	Medit.
*Laphangium luteoalbum* (L.) Tzvelev	T scap	Subcosmop.
*Leontodon tuberosum* L	H ros	Medit.
*Linum bienne* Mill.	T scap	Medit.-Atl.
*Linum strictum* L.	T scap	Medit.
*Linum trigynum* L.	T scap	Euro-Medit.
*Linum usitatissimum* L. subsp. *angustifolium* (Huds.) Thell.	H bienn	Euro-Medit.
*Logfia gallica* (L.) Cosson et Germ.	T scap	Euro-Medit.
*Lolium multiflorum* Lam.	T scap	Euro-Medit.
*Lolium perenne* L.	H caesp	Euro-Medit.
*Lolium rigidum* Gaudin	T scap	Subtrop.
*Lotus angustissimus* L.	T scap	Euro-Medit.
*Lotus conimbricensis* Brot.	T scap	Medit.
*Lotus edulis* L.	T scap	Medit.
*Lotus hispidus* DC.	T scap	Medit.-Atl.
*Lotus ornithopodioides* L.	T scap	Medit.
*Lotus parviflorus* Desf.	T scap	Medit.
*Lotus pedunculatus* Cav.	H scap	Paleotemp.
*Lysimachia minima* (L.) U.Manns et Anderb.	T scap	Euro.-Asiat.
*Lythrum hyssopifolia* L.	T scap	Subcosmop.
*Lythrum tribracteatum* Spreng.	T scap	Euro-Medit.
*Macrobriza maxima* (L.) Tzvelev	T scap	Subtrop.
*Malva sylvestris* L.	T scap	Euro-Medit.
*Medicago arabica* (L.) Huds.	T scap	Medit.
*Medicago minima* (L.) L.	T scap	Euro-Medit.
*Medicago polymorpha* L.	T scap	Medit-Iran-Turan.
*Medicago truncatula* Gaertn.	T scap	Medit.
*Mentha pulegium* L.	H scap	Euro-Medit.
*Middendorfia borysthenica* (Schrank) Trautv.	T scap	Euro-Medit.
*Molineriella minuta* (L.) Rouy	T scap	Medit.
*Montia arvensis* Wallr.	I rad	Medit.-Atl.
*Montia hallii* (A.Gray) Greene	I rad	Euro-Medit.
*Montia variabilis* (Walters) Landolt	I rad	Medit.-Atl.
*Moraea sisyrinchium* (L.) Ker Gawl.	G bulb	Medit-Iran-Turan.
*Myosotis sicula* Guss.	T scap	N Medit.
*Myriophyllum verticillatum* L.	I rad	Circumbor.
*Nasturtium officinale* W.T.Aiton	H scap	Cosmop.
*Oenanthe fistulosa* L.	H scap	Euro.-Asiat.
*Oenanthe lisae* Moris	H scap	Endem. Sard.
*Oenanthe pimpinelloides* L.	H scap	Medit.-Atl.
*Ophioglossum lusitanicum* L.	T scap	Paleotemp.
*Oxybasis chenopodioides* (L.) S.Fuentes, Uotila et Borsch	T scap	Subcosmop.
*Paronychia echinulata* Chater	T scap	Medit.
*Paspalum distichum* L.	G rhiz	Subcosmop.
*Peplis portula* L.	T rept	Euro.-Asiat.
*Persicaria lapathifolia* (L.) Delarbre	T scap	Circumbor.
*Petrorhagia dubia* (Raf.) G.López et Romo	T scap	S Medit.
*Phalaris coerulescens* Desf.	H caesp	Medit.
*Pilularia minuta* Durieu	I rad	Medit.
*Plantago bellardii* All.	T scap	Medit.
*Plantago coronopus* L.	T scap	Euro-Medit.
*Plantago intermedia* Gilib.	H ros	Euro-Medit.
*Plantago lanceolata* L.	T scap	Euro.-Asiat.
*Plantago weldenii* Rchb.	H ros	Euro-Medit.
*Poa annua* L.	T scap	Cosmop.
*Poa infirma* Kunth	T scap	Euro-Medit.
*Polycarpon tetraphyllum* (L.) L.	T scap	Euro-Medit.
*Polygonum aviculare* L.	T rept	Cosmop.
*Polypogon maritimus* Willd.	T scap	Medit-Iran-Turan.
*Polypogon subspathaceus* Req.	T scap	Medit.
*Polypogon viridis* (Gouan) Breistr.	H caesp	Paleotrop.
*Portulaca oleracea* L.	T scap	Subcosmop.
*Potentilla reptans* L.	H rept	Paleotemp.
*Prospero autumnale* (L.) Speta	G bulb	Euro-Medit.
*Pulicaria odora* (L.) Rchb.	H scap	Euro-Medit.
*Pulicaria sicula* (L.) Moris	T scap	Medit.
*Pulicaria vulgaris* Gaertn.	T scap	Paleotemp.
*Radiola linoides* Roth.	T scap	Paleotemp.
*Ranunculus baudotii* Godr.	I rad	Medit.-Atl.
*Ranunculus cordiger* Viv. subsp. *diffusus* (Moris) Arrigoni	H scap	Endem. Sard.
*Ranunculus lateriflorus* DC.	T scap	Paleotrop.
*Ranunculus muricatus* L.	T scap	Euro-Medit.
*Ranunculus ophioglossifolius* Vill.	T scap	Euro-Medit.
*Ranunculus paludosus* Poir.	H scap	Medit-Iran-Turan.
*Ranunculus revelieri* Boreau	T scap	W Medit.
*Ranunculus saniculifolius* Viv.	I rad	Medit.
*Ranunculus sardous* Crantz	T scap	Euro-Medit.
*Ranunculus trilobus* Desf.	T scap	Medit.
*Raphanus raphanistrum* L.	T scap	Circumbor.
*Reichardia picroides* (L.) Roth	H scap	Medit.
*Reseda luteola* L.	H bienn	Circumbor.
*Romulea columnae* Sebast. & Mauri	G bulb	Medit.
*Romulea ramiflora* Ten.	G bulb	Medit.
*Romulea requienii* Parl.	G bulb	Endem. Sard.-Cors.
*Rumex bucephalophorus* L.	T scap	Medit.
*Rumex obtusifolius* L.	H scap	Cosmop.
*Rumex sanguineus* L.	H scap	Euro-Medit.
*Sagina hawaiensis* Pax	H caesp	Euro-Medit.
*Sagina maritima* Don	T scap	Medit.-Atl.
*Samolus valerandi* L.	H scap	Subcosmop.
*Schenkia spicata* (L.) G.Mans.	T scap	Euro-Medit.
*Sedum andegavense* (DC.) Desv.	T scap	W Medit.
*Sedum caeruleum* L.	T succ	SW Medit.
*Sedum glandulosum* Moris	H succ	W Medit.
*Selaginella denticulata* (L.) Spring	H rept	Euro-Medit.
*Serapias lingua* L.	G bulb	Medit.
*Sesamoides interrupta* (Boreau) G.López	H scap	W Medit.
*Sherardia arvensis* L.	T scap	Euro-Medit.
*Silene gallica* L.	T scap	Euro-Medit.
*Solanum nigrum* L.	T scap	Euro.-Asiat.
*Solenopsis laurentia* (L.) C.Presl subsp. *laurentia*	T scap	Medit.
*Sonchus asper* (L.) Hill	T scap	Euro.-Asiat.
*Spergularia marina* (L.) Besser	T scap	Subcosmop.
*Spergularia rubra* (L.) J.Presl et C.Presl.	T scap	Subcosmop.
*Sporobolus aculeatus* (L.) P.M.Peterson	T scap	Paleotemp.
*Sporobolus schoenoides* (L.) P.M.Peterson	T scap	Paleotemp.
*Symphyotrichum squamatum* (Spreng.) G.L.Nesom	H scap	Avv.
*Teesdalia nudicaulis* (L.) W.T.Aiton	T scap	Medit.-Atl.
*Tolpis umbellata* Bertol.	T scap	Medit.
*Trachynia distachya* (L.) Link	T scap	Medit-Iran-Turan.
*Trifolium angustifolium* L.	T scap	Euro-Medit.
*Trifolium arvense* L.	T scap	Paleotemp.
*Trifolium bocconei* Savi	T scap	Medit.
*Trifolium campestre* Schreb.	T scap	Paleotemp.
*Trifolium dubium* Sibth.	T scap	Euro-Medit.
*Trifolium leucanthum* M.Bieb.	T scap	E Medit.
*Trifolium michelianum* Savi	T scap	W Medit.
*Trifolium micranthum* Viv.	T scap	Paleotemp.
*Trifolium nigrescens* Viv.	T scap	Euro-Medit.
*Trifolium resupinatum* L.	T scap	Paleotemp.
*Trifolium scabrum* L.	T scap	Euro-Medit.
*Trifolium squarrosum* L.	T scap	Medit.
*Trifolium striatum* L.	T scap	Paleotemp.
*Triglochin barrelieri* Loisel.	G bulb	Medit.
*Triticum vagans* (Jord. et Fourr.) Greuter	T scap	Medit-Iran-Turan.
*Tuberaria guttata* (L.) Fourr.	T scap	Euro-Medit.
*Verbena officinalis* L.	H scap	Subcosmop.
*Verbena supina* L.	T scap	Euro-Medit.
*Verojuncus pygmaeus* (Rich. ex Thuill.) Záveská Drábková et Proćków	T scap	Medit.-Atl.
*Verojuncus tingitanus* (Maire et Weiller) Záveská Drábková et Proćków	T caesp	W Medit.
*Veronica beccabunga* L.	H rept	Euro-Asiat.
*Veronica praecox* All.	T scap	Euro-Medit.
*Vicia sativa* L.	T scap	Medit.
*Vulpia myuros* (L.) C.C.Gmel.	T scap	Subcosmop.
*Xanthium spinosum* L.	T scap	Avv.

## Appendix B

Phytosociological tables quoted in the text.
